# A Systematic Review and User Reference of Phenotypic and Molecular Characteristics of Dexamethasone‐Mediated C2C12 Muscle Atrophy

**DOI:** 10.1002/jcsm.70127

**Published:** 2026-03-13

**Authors:** Alexa J. Klein, Roger A. Vaughan

**Affiliations:** ^1^ Department of Health and Human Performance High Point University High Point North Carolina USA

**Keywords:** Atrogin‐1, atrophy, C2C12 myotubes, dexamethasone, MuRF1, sarcopenia

## Abstract

**Background:**

Skeletal muscle is a vital part of human physiology and is responsible for numerous essential functions. Not surprisingly, the loss of skeletal muscle mass and function is common in several pathologies including atrophy and sarcopenia, which profoundly impact quality of life of those afflicted. Thus, numerous investigations of potential therapies for mitigating or reversing such pathologies are available. Within these studies, experimental cell culture models such as the murine C2C12 myoblasts are commonly used. Over 100 publications have utilized dexamethasone‐treated C2C12 myotubes to investigate various aspects of muscle atrophy. The purpose of this systematic review is to describe the experimental conditions common to these experiments, as well as phenotypical myotube presentation, and gene and protein expression of targets that regulate muscle mass, function, and metabolism.

**Methods:**

A systematic review of literature was conducted until 3 January 2025 using PUBMED. Articles were included if (1) C2C12 myotubes were used, (2) the article included a dexamethasone‐only group along with appropriate vehicle or true control and (3) the article assessed at least one of the related phenotypical or molecular outcomes of importance to the scope of the review.

**Results:**

A total of 182 articles were included after screening for relevance and inclusion criteria, which were assessed for outcomes (raw data reported when available or using ratio‐metric estimates of relative differences between dexamethasone treatment and control). In 24 of 26 unique experiments that utilized 10 μM dexamethasone and 37 of 39 unique experiments that utilized 100 μM dexamethasone, a decrease in myotube diameter was reported (pooled experimental average estimates from 24‐h time points 69.8% ± 7.5% and 66.9% ± 14.7% for 10 and 100 μM, respectively, vs. control). All six studies that utilized 10 μM dexamethasone and all nine that treated myotubes with 100 μM dexamethasone reported reduced fusion index (pooled experimental average estimates from 24‐h time points: 67.6% ± 5.3% and 68.4% ± 8.4% for 10 and 100 μM, respectively, vs. control). Dexamethasone‐treated myotubes also consistently expressed increased atrophic‐related molecular targets including Atrogin‐1 and muscle atrophy X box 1 (MuRF1), as well as reductions in anabolic signalling (specifically, mTORC and Akt activation) and mitochondrial function.

**Conclusions:**

The striking consistency of these findings suggests dexamethasone treatment of C2C12 myotubes is a reliable method of mimicking many features common to skeletal muscle pathology. This review provides insight into the use and expected outcomes of the dexamethasone‐mediated model of atrophy in C2C12 myotubes and may serve as a helpful reference for future experiments utilizing this model.

**Abbreviations**
4EBP1: eukaryotic translation initiation factor 4E‐binding protein 1
AMPK: AMP‐activated protein kinase (protein)
ATP: adenosine triphosphate (metabolite)
Foxo1: forkhead box protein O1 (mRNA)
FOXO1: forkhead box protein O1 (protein)
Foxo3: forkhead box protein O3 (mRNA)
FOXO3: forkhead box protein O3 (protein)
GR: glucocorticoid receptor (protein)
GRE: glucocorticoid response element (DNA binding region)
HSP90: heat shock protein 90 (protein)
IGF1: insulin‐like growth factor 1 (protein)
IGF1R: insulin‐like growth factor 1 receptor (protein)
IRS1: insulin receptor substrate 1 (protein)
IR: insulin receptor (protein)
Mafbx and/or Fbxo32: muscle atrophy X box (mRNA of Atrogin‐1)
MHC: myosin heavy chain (protein from varied isoforms)
MURF1: muscle RING‐finger protein‐1 (protein)
Mstn: myostatin (mRNA)
MSTN: myostatin (protein)
MSTNR: myostatin receptor (protein)
mTOR: mechanistic/mammalian target of rapamycin (protein)
mTORC1: mechanistic/mammalian target of rapamycin complex 1 (protein)
mTORC2: mechanistic/mammalian target of rapamycin complex 2 (protein)
Myh: myosin heavy chain (mRNA from varied transcripts)
Myod: myoblast determination protein 1 (mRNA)
MYOD: myoblast determination protein 1 (protein)
Nrf1: nuclear respiratory factor 1 (mRNA)
NRF1: nuclear respiratory factor 1 (protein)
PI3K: phosphatidylinositol 3‐kinase (protein)
Ppargc1a: peroxisome proliferator‐activated receptor gamma coactivator 1‐alpha (mRNA)
PGC‐1α: peroxisome proliferator‐activated receptor gamma coactivator 1‐alpha (protein)
P70s6k: Ribosomal protein S6 kinase (protein)
Sirt1: sirtuin 1 (mRNA)
SIRT1: sirtuin 1 (protein)
Tfam: mitochondrial transcription factor A (mRNA)
TFAM: mitochondrial transcription factor A (protein)
Trim63 and/or Murf1: tripartite motif containing 63 (mRNA of Murf1)
TRP: tetratricopeptide repeat protein (protein)
YY1: Yin Yang 1 (protein)

## 1 Introduction

Skeletal muscle is a vital part of human physiology and is responsible for numerous essential functions. Skeletal muscle dysfunction and/or loss of skeletal muscle mass have been implicated in several pathologies that profoundly affect the quality of life of the afflicted. Thus, exploring the mechanisms of disease in skeletal muscle offers the possibility of treatments that may improve or resolve these muscle‐related disorders. One such approach for studying muscle physiology is the use of in vitro systems such as the myoblast cell culture line, C2C12. This cell line was originally isolated from mouse skeletal muscle and is highly culturable under standard conditions [1]. The cell line is also capable of differentiating into multinucleated myotubes, which makes it a convenient and effective model for studying several attributes of muscle physiology during health and disease [1]. The loss of muscle mass during atrophy and/or sarcopenia is among the most researched topics using the C2C12 myotube model. While there are several stimuli known to induce atrophy in cultured myotubes, treatment with dexamethasone is among the most common. In fact, over 100 peer‐reviewed primary research articles have employed this technique, most of which have explored various methods of treating or resolving the condition. Given the increasingly prevalent use of the C2C12 myotube model as well as the ubiquitous use of dexamethasone to induce muscle damage in this model, there is a need to summarize the common features of dexamethasone‐mediated myopathy. Therefore, this review was undertaken to provide a summary of common features of dexamethasone‐mediated muscle damage reported in C2C12 myotubes for future experimentalists and consumers of muscle pathology‐related research.

### 1.1 Mechanistic Overview of Dexamethasone‐Mediated Induction of Atrophy

**FIGURE 1:**
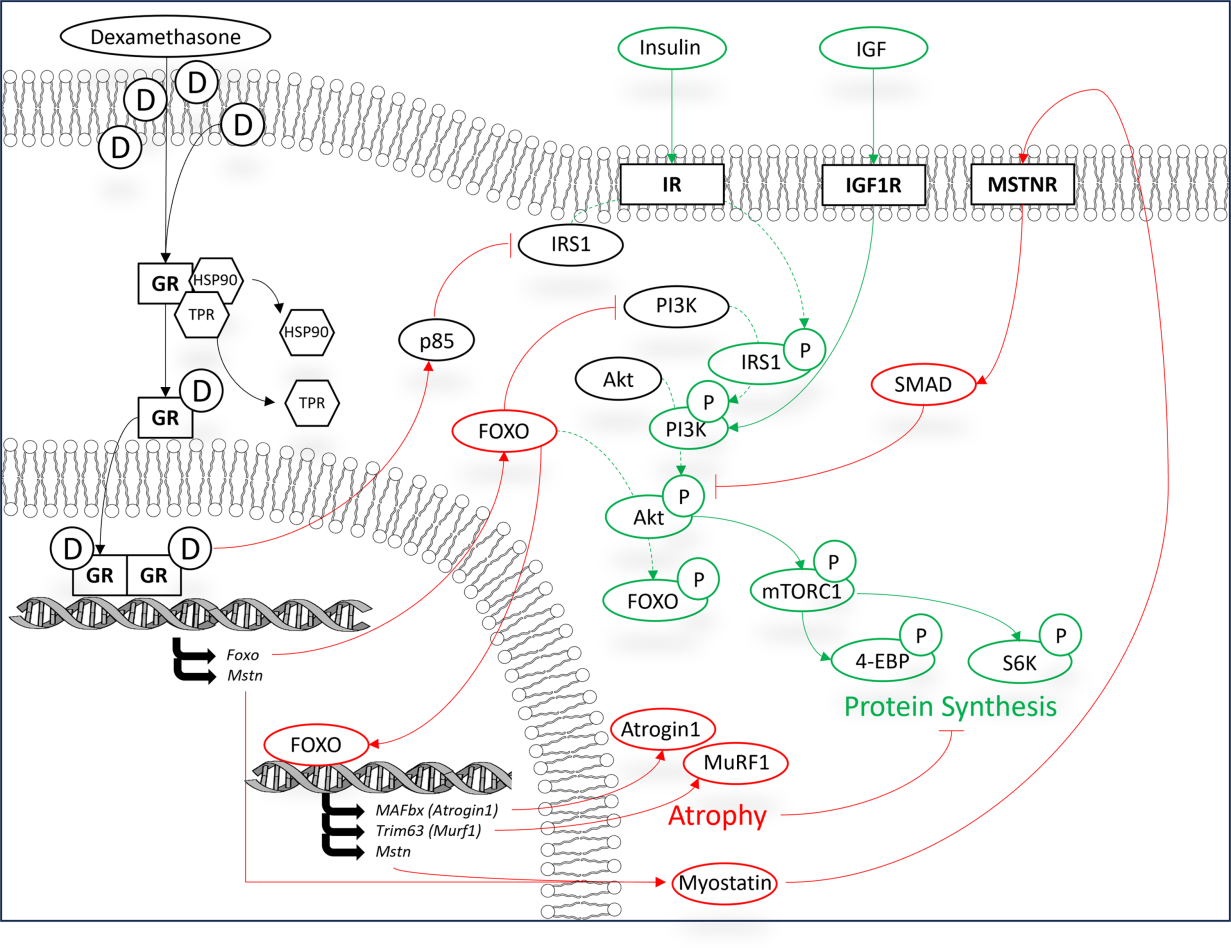
Dexamethasone‐mediated muscle atrophy and related signalling cascades. *Note:* Solid black arrows denote signalling associated with dexamethasone signalling. Green arrows denote activation of anabolic signalling and protein synthesis, which oppose atrophy‐related response. Dashed green arrows that travel behind a molecular target indicate that the molecular target over the dashed arrow phosphorylates the target from which dashed arrow originates and promotes anabolic signalling. Red arrows denote activation of atrophy related response and protein breakdown, which oppose anabolic signalling and protein synthesis. Abbreviations: dexamethasone (D); forkhead box protein O1 (Foxo1); glucocorticoid receptor (GR); heat shock protein 90 (HSP90); glucocorticoid response elements (GRE); insulin receptor substrate 1 (IRS1); insulin receptor (IR); muscle atrophy X box (Mafbx or Fbxo32); muscle RING‐finger protein‐1 (MuRF1); phosphatidylinositol 3‐kinase (PI3K); mammalian target of rapamycin complex 1 (mTORC1); myostatin (Mstn); tetratricopeptide repeat protein (TPR); tripartite motif containing 63 (Trim63 or Murf1).

In general, several aspects of dexamethasone‐mediated signalling are understood and reviewed in several articles discussed below. Proximally, dexamethasone binds its receptor (predominantly located within the cytosol when inactive), the glucocorticoid receptor (GR) [2]. Next, the dexamethasone‐bound (ligand‐bound) receptor dissociates from its negative regulatory chaperone proteins, heat shock protein 90 (HSP90) and tetratricopeptide repeat (TPR) protein [2]. The ligand‐bound receptor complex can then translocate to the nucleus through nuclear pores, form a homodimer [3] and, through multiple mechanisms [3], activate or suppress transcription of several targets by binding the glucocorticoid response elements (GRE) [2, 3] or other response elements [3]. One of the primary targets of GR is the forkhead box protein O1 (FOXO1 protein), which is responsible for coordinating and upregulating the expression of atrophy‐related genes [4], including muscle atrophy X box (*Mafbx* or *Fbxo32*) and tripartite motif containing 63 (*Trim63* or *Murf1*), which code for Atrogin‐1 and muscle RING‐finger protein‐1 (MuRF1), respectively. Dexamethasone has also been shown to upregulate p85α (but not p85β) [5], leading to reduced insulin receptor substrate 1 (IRS1) and phosphatidylinositol 3‐kinase (PI3K) activity [5, 6]. Such inhibition of IRS‐1/PI3K signalling by dexamethasone could reduce the activation of downstream targets including Akt. Furthermore, reduced Akt activation reduces the activation of the mammalian target of rapamycin complex 1 (mTORC1), a master regulator of protein synthesis [7]. Additionally, ligand‐bound GR also regulates the expression of myostatin (*Mstn*), a transcript that codes for the myostatin protein (MSTN), which is a known negative regulator of muscle protein synthesis and hypertrophy [7]. Specifically, ligand‐bound GR increases *Mstn* expression both directly via enhanced transcription of the *Mstn* gene [8] and through increased FOXO stimulation [9]. Further downstream, myostatin is known to bind its receptor, which promotes SMAD activity, which impedes activation of Akt [7]. Collectively, dexamethasone increases the expression of targets associated with muscle protein breakdown while simultaneously reducing the activity of targets that positively regulate muscle protein synthesis (Figure 1). Additionally, mitochondrial dysfunction also appears to be a consistent feature of atrophy [10]; however, the exact mechanism(s) for this association remain unclear (though the link between mitochondrial function/dysfunction and skeletal muscle size and function has previously been discussed in detail [11]). It has previously been shown that reductions in mitochondrial function and ATP content may precede atrophic signalling [12]. Possible mechanisms could be that dexamethasone treatment increases mitochondrial oxidative stress [13] or reduces mitochondrial membrane potential [14], leading to reduced mitochondrial function. There is also limited data that dexamethasone reduces Yin Yang 1 (YY1) [15], which is known to work with mTORC1 to regulate mitochondrial content and function [16]. However, the proposed mechanisms of how dexamethasone reduces mitochondrial content and function are speculative and require additional experimentation.

Collectively, dexamethasone treatment has several effects on various molecular targets, which together coordinate an atrophy‐promoting environment. Here, we summarized the effects of dexamethasone treatment on various aspects of C2C12 myotubes (commonly used for mechanistic studies that may have implications for muscle‐wasting diseases). The topics are described in Table S1 (per PRISMA guidelines) and organized as follows: cell viability (Table 1), myotube physiology such as diameter and fusion (Table 2), myotube differentiation factors (Table S2), atrophy‐related signalling including Atrogin‐1, Murf1 and myostatin (Table S3), FOXO1/3 signalling (Table S4) and effect on protein synthetic signalling (Table S5). Additionally, because atrophy‐related diseases are often associated with impaired energetics, we also summarize the effects of dexamethasone treatment on the regulation of mitochondrial biogenesis signalling (Table S6) and mitochondrial function and content (Table S7). In general, the findings from our investigation show clear and consistent evidence that dexamethasone reduces myotube size, formation, and protein synthesis, along with concurrent upregulation of atrophy‐related signalling and protein degradation. These findings also illustrate a consistent suppression of mitochondrial function and content in dexamethasone‐treated myotubes.

**TABLE 1:** Effect of dexamethasone on cultured myotube viability.

Concentration	Duration	Viability	Reference
0.01 μM	48 h	↔ (≈ 95.3% ± 5%)	Lee et al. [17]
0.025 μM	48 h	↓ (≈ 87.2% ± 5%)	Lee et al. [17]
0.05 μM	48 h	↓ (≈ 83.2% ± 5%)	Lee et al. [17]
0.075 μM	48 h	↓ (≈ 77.9% ± 5%)	Lee et al. [17]
0.1 μM	24 h	↔ (≈ 100% ± 5%)	Wang et al. [18]
0.1 μM	48 h	↓ (≈ 75.5% ± 5%)	Lee et al. [17]
0.2 μM	48 h	↓ (≈ 69.7% ± 5%)	Lee et al. [17]
0.4 μM	48 h	↓ (≈ 69.7% ± 5%)	Lee et al. [17]
0.6 μM	48 h	↓ (≈ 67.4% ± 5%)	Lee et al. [17]
1 μM	24 h	↔ (≈ 96.9% ± 5%)	Wang et al. [18]
1 μM	48 h	↔ (92.3% ± 0.6%)	Micheli et al. [19]
5 μM	24 h	↓ ≈ (78.6% ± 5%)	An et al. [20]
5 μM	24 h	↓ ≈ (54.8% ± 5%)	Lee et al. [21]
5 μM	24 h	↓ (≈ 77.2% ± 5%)	Kim et al. [22]
10 μM	12 h	↔ (≈ 96.9% ± < 5% SEM)	Ma et al. [23]
10 μM	24 h	↓ (≈ 64.5% ± < 5%)	Amarasiri et al. [24]
10 μM	24 h	↓ (≈ 77.0% ± < 5%)	Kurera et al. [25]
10 μM	24 h	↓ (≈ 63.4% ± < 5%)	Son et al. [26]
10 μM	24 h	↓ (≈ 61.9% ± < 5%)	Salucci et al. [27]
10 μM	24 h	↓ (≈ 81.5% ± < 5%)	Wang et al. [18]
10 μM	24 h	↓ (≈ 81.3% ± 5%)	Kim et al. [13]
10 μM	24 h	↓ (≈ 73.5% ± 5%)	Shen et al. [28]
10 μM	24 h	↔ (≈ 97.5% ± 5%)	Hur er al. [29]
10 μM	24 h	↓ (≈ 72.5% ± 5%)	Ma et al. [30]
10 μM	24 h	↔ (≈ 98.4% ± 5% SEM)	Ma et al. [23]
10 μM	48 h	↔ (≈ 96.8% ± 5% SEM)	Ma et al. [23]
10 μM	48 h	↓ (≈ 71.1% ± 5%)	Chen et al. [31]
25 μM	24 h	↓ (≈ 81.4% ± < 5%) SEM	Kim et al. [32]
25 μM	24 h	↔ (≈ 94.5% ± 10%)	Men et al. [33]
25 μM	24 h	↔ (≈ 89.2% ± 5%)	Wang et al. [34]
25 μM	48 h	↔ (≈ 83.0% ± < 5%)	Wang et al. [34]
25 μM	24 h	↔ (≈ 94.7% ± 5%)	Park et al. [35]
30 μM	24 h	↓ (≈ 67.7% ± 5%)	Ma et al. [30]
50 μM	24 h	↓ (≈ 64.5% ± 5%)	Ma et al. [30]
50 μM	24 h	↓ (≈ 78.5% ± < 5%) SEM	Kim et al. [32]
50 μM	24 h	↓ (≈ 90.2% ± 10%)	Men et al. [33]
50 μM	24 h	↔ (≈ 100% ± 5%)	Park et al. [35]
50 μM	24 h	↔ (≈ 89.2% ± < 5%)	Wang et al. [34]
50 μM	24 h	↓ (≈ 60.4% ± 5%) SEM	Sun et a. [36]
50 μM	24 h	↓ (≈ 83.1% ± 5%)	Hur er al. [29]
50 μM	48 h	↔ (≈ 83.0% ± < 5%)	Wang et al. [34]
100 μM	24 h	↓ (≈ 62.9% ± 5%)	Ma et al. [30]
100 μM	24 h	↔ (≈ 100.0% ± 10%)	Lee et al. [37]
100 μM	24 h	↔ (≈ 96.2% ± 10%)	Lee et al. [38]
100 μM	24 h	↔ (≈ 97.3% ± 5%)	Park et al. [35]
100 μM	24 h	↔ (≈ 76.9% ± < 5%)	Wang et al. [34]
100 μM	24 h	↔ (≈ 92.7% ± 10%)	Kim et al. [39]
100 μM	24 h	↓ (≈ 81.5% ± 10%)	Men et al. [33]
100 μM	24 h	↓ (≈ 75.7% ± < 5%) SEM	Kim et al. [32]
100 μM	24 h	↓ (≈ 77.1% ± 5%)	Hur er al. [29]
100 μM	24 h	↓ (≈ 67.6% ± < 5%) SEM	Nguyen et al. [40]
100 μM	24 h	↔ (≈ 101.4% ± 5%) SEM	Han et al. [41]
100 μM	24 h	↓ (≈ 63.2% ± 5%)	Jo et al. [42]
100 μM	24 h	↓ (≈ 77.4% ± < 5%) SEM	Choi et al. [43]
100 μM	24 h	↓ (≈ 73.1% ± < 5%) SEM	Ko et al. [44]
100 μM	48 h	↔ (≈ 76.9% ± < 5%)	Wang et al. [34]
100 μM	48 h	↓ (≈ 70.9% ± < 5%)	Jang et al. [45]
100 μM	72 h	↔ (≈ 93.7% ± 5%)	Lee et al. [46]
125 μM	4 h	↓ (≈ 79.0% ± 5%)	Chen et al. [47]
200 μM	24 h	↓ (≈ 75.0% ± 5%)	Park et al. [35]
200 μM	24 h	↓ (≈ 58.4% ± < 5%)	Wang et al. [34]
200 μM	24 h	↓ (≈ 75.0% ± 10%)	Men et al. [33]
200 μM	24 h	↓ (≈ 76.5% ± 5%)	Men et al. [48]
200 μM	24 h	↓ (≈ 68.2% ± 5%)	Jiang et al. [49]
200 μM	24 h	↓ (≈ 73.1% ± < 5%) SEM	Kim et al. [32]
200 μM	24 h	↓ (≈ 82.7% ± 10%)	Kim et al. [50]
200 μM	48 h	↓ (≈ 52.3% ± < 5%)	Wang et al. [34]
250 μM	4 h	↓ (≈ 60.4% ± 5%)	Chen et al. [47]
400 μM	24 h	↓ (≈ 63.1% ± 5%)	Park et al. [35]
500 μM	4 h	↓ (≈ 53.0% ± 5%)	Chen et al. [47]
1000 μM	4 h	↓ (≈ 50.6% ± 5%)	Chen et al. [47]

*Note:* Columns are reported as raw values (if available) or as estimates (indicated by ≈) of treatment group expressed as a per cent of control ± the variability for the treated group. Variability is listed as SD unless noted with another reporting value (such as SEM).

**TABLE 2:** Effect of dexamethasone on myotube diameter and fusion.

Concentration	Duration	Diameter/size	Fusion index	Reference
0.001 μM	24 h	↔ (≈ 107.0% ± > 10%)		Tsuchida et al. [51]
0.01 μM	12 h	↓ (≈ 78.5% ± 5%) SEM		Jia et al. [52]
0.01 μM	24 h	↔ (≈ 97.1% ± > 10%)		Tsuchida et al. [51]
0.01 μM	72 h	↔ (≈ 102.3% ± > 10%)		Sultan et al. [53]
0.01 μM	192 h	↓ (≈ 60.7% ± 5%) SEM	↓ (≈ 47.8% ± 5%) SEM	Kim et al. [54]
0.05 μM	48 h	↓ (≈ 61.3% ± > 10%)		Lee et al. [17]
0.05 μM	48 h	↓ (≈ 51.8% ± > 10%)		Li et al. [55]
0.1 μM	24 h	↔ (≈ 90.1% ± > 10%)		Tsuchida et al. [51]
0.1 μM	48 h	↓ (≈ 58.8% ± > 10%)		Sun et al. [56]
0.1 μM	72 h	↔ (≈ 115.2% ± > 10%)		Sultan et al. [53]
0.1 μM	192 h	↓ (≈ 21.5% ± 5%) SEM	↓ (≈ 23.9% ± 5%) SEM	Kim et al. [54]
1 μM	5 h		↓ (≈ 64.4% ± < 5%)	Kim et al. [57]
1 μM	24 h	↓ (≈ 80.0% ± > 10%)		Son et al. [58]
1 μM	24 h	↓ (≈ 83.0% ± > 10%)		Tsuchida et al. [51]
1 μM	24 h	↔ (≈ 91.6% ± 5%)		Menconi et al. [59]
1 μM	24 h	↓ (≈ 72.7% ± > 10%)		Lu et al. [60]
1 μM	24 h	↓ (≈ 60.9% ± 5%)		Cid‐Diaz et al. [61]
1 μM	24 h	↓ (≈ 89.4% ± > 10%)		Katsuki et al. [62]
1 μM	24 h	↓ (≈ 54.2% ± 5%)		Le Bacquer et al. [63]
1 μM	24 h	↓ (≈ 43.5% ± 5%)		Sawano et al. [64]
1 μM	24 h	↓ (≈ 53.0% ± 5%)		Habibian et al. [65]
1 μM	24 h	↓ (≈ 54.1% ± > 10%)		Yoshioka et al. [66]
1 μM	24 h	↓ (≈ 68.0% ± 5%)		Morano et al. [67]
1 μM	24 h	↓ (≈ 45.0% ± > 10%)		Hsieh et al. [68]
1 μM	48 h	↔ (≈ 101.6% ± 5%) SEM		Han et al. [69]
1 μM	48 h	↓ (≈ 30.1% ± 5%) SEM		Singh et al. [70]
1 μM	48 h	↓ (≈ 72.6% ± 5%)		Menconi et al. [59]
1 μM	48 h		↓ (varied)	Han et al. [71]
1 μM	48 h	↓ (≈ 62.5% ± > 10%)		Hah et al. [72]
1 μM	48 h	↓ (≈ 62.5% ± > 10%)	↓ (≈ 41.9% ± > 10%)	Hah et al. [73]
1 μM	48 h	↓ (≈ 60.2% ± > 10%)	↓ (≈ 52.3% ± > 10%)	Micheli et al. [19]
1 μM	48 h	↓ (≈ 65.0% ± 5%)	↓ (≈ 73.8% ± 5%)	Di Cesare Mannelli et al. [74]
1 μM	48 h	↓ (≈ 57.7% ± > 10%)	↓ (≈ 70.0% ± > 10%)	Hah et al. [75]
1 μM	72 h	↓ (≈ 81.3% ± > 10%)		Archer‐Lahlou et al. [76]
1 μM	192 h	↓ (≈ 9.8% ± 5%) SEM	↓ (≈ 10.8% ± 5%) SEM	Kim et al. [54]
5 μM	24 h	↓ (≈ 42.6% ± 5%) SEM		An et al. [20]
5 μM	24 h	↓ (≈ 58.0% ± > 10%)		Choi et al. [77]
5 μM	24 h	↓ (≈ 55.0% ± > 10%)		Lee et al. [21]
5 μM	24 h	↓ (≈ 83.3% ± > 10%)		Kim et al. [78]
5 μM	24 h	↓ (≈ 64.1% ± > 10%)		Kim et al. [22]
5 μM	24 h	↓ (≈ 76.2% ± > 10%)	↓ (≈ 79.5% ± > 10%)	Kim et al. [79]
5 μM	24 h		↓ (≈ 51.2% ± > 10%)	Lee et al. [80]
5 μM	24 h	↓ (≈ 45.7% ± 5%)		Son et al. [81]
5 μM	24 h	↓ (≈ 76.3% ± 5%)		Eo et al. [82]
5 μM	48 h	↓ (≈ 67.1% ±?%)		Gan et al. [83]
10 μM	12 h	↔ (≈ 101.4% ± > 10%)		Tsuchida et al. [51]
10 μM	24 h	↓ (≈ 63.9% ± > 10%)		Lee et al. [84]
10 μM	24 h	↓ (≈ 63.6% ± 5%) SEM		McClung et al. [85]
10 μM	24 h	↓ (≈ 73.6% ± 5%) SEM		Ma et al. [30]
10 μM	24 h	↓ (≈ 77.4% ± > 10%)		Tsuchida et al. [51]
10 μM	24 h	↓ (≈ 77.9% ± > 10%)		Bowen et al. [86]
10 μM	24 h	↓ (≈ 72.6% ± > 10%)		Ulla et al. [87]
10 μM	24 h	↓ (≈ 77.7% ± > 10%)	↓ (≈ 70.0% ± > 10%)	Lee et al. [88]
10 μM	24 h	↓ (≈ 66.6% ± > 10%)		Kimira et al. [89]
10 μM	24 h	↓ (≈ 70.4% ± 5%)		Amarasiri et al. [24]
10 μM	24 h	↓ (≈ 57.1% ± 5%)		Kurera et al. [25]
10 μM	24 h	↓ (≈ 76.9% ± < 5%)	↓ (≈ 61.5% ± < 5%)	Son et al. [26]
10 μM	24 h	↓ (≈ 77.9% ± > 10%)		Rahman et al. [90]
10 μM	24 h	↓ (≈ 62.8% ± > 10%)		Salucci et al. [27]
10 μM	24 h	↓ (≈ 77.7% ± > 10%)		Kim et al. [14]
10 μM	24 h	↓ (≈ 64.4% ± > 10%)		Kim et al. [91]
10 μM	24 h	↓ (≈ 55.1% ± > 10%)		Zhiyin et al. [92]
10 μM	24 h	↓ (varied)		Wang et al. [18]
10 μM	24 h	↓ (≈ 71.4% ± 5%)	↓ (≈ 71.4% ± 5%)	Kim et al. [13]
10 μM	48 h	↓ (≈ 69.7% ± 5%) SEM	↓ (≈ 65.7% ± 5%) SEM	Yang et al. [93]
10 μM	48 h	↓ (≈ 80.9% ± 5%)		Kim et al. [94]
10 μM	48 h		↓ (≈ 411% ± 5%)	Pansters et al. [95]
10 μM	48 h	↔ (≈ 95.0% ± 5%) SEM		Han et al. [69]
10 μM	48 h	↓ (≈ 50.0% ± 5%)		Chen et al. [31]
10 μM	48 h	↓ (≈ 82.2% ± 5%)		Gurjar et al. [96]
10 μM	48 h	↓ (≈ 49.0% ± > 10%)		Park et al. [97]
10 μM	48 h	↓ (≈ 45.0% ± > 10%)		Hyun et al. [98]
10 μM	96 h		↓ (≈ 24.6% ± 5%)	Ma et al. [23]
40 μM	24 h	↓ (≈ 79.3–83.6% ± > 10%)		Aguilar‐Agon et al. [99]
50 μM	24 h	↓ (≈ 89.2% ± 5%)		Nguyen et al. [100]
50 μM	24 h	↓ (≈ 61.3% ± > 10%)		Li et al. [101]
50 μM	24 h	↓ (≈ 43.2% ± 5%)		Yoo et al. [102]
50 μM	24 h	↓ (≈ 28.5% ± 5%)		Li et al. [103]
50 μM	24 h	↓ (≈ 59.4% ± 5%) SEM		Li et al. [15]
50 μM	36 h	↓ (≈ 68.5% ± 5%) SEM		Li et al. [104]
50 μM (reported as 50 mM)	48 h	↓ (≈ 47.9% ± 5%)		Liu et al. [105]
50 μM	48 h	↓ (≈ 71.1% ± 10%)		Kim et al. [106]
50 μM	48 h	↓ (≈ 74.0% ± 10%)		Jeon et al. [107]
50 μM	48 h	↓ (≈ 76.1% ± > 10%)	↓ (≈ 60.8% ± > 10%)	Liang et al. [108]
100 μM	24 h	↔ (≈ 87.5% ± > 10%)		Oelkrug et al. [109]
100 μM	24 h	↔ (≈ 76.2% ± > 10%)		Maier et al. [110]
100 μM	24 h	↓ (≈ 75.6% ± > 10%)		You et al. [111]
100 μM	24 h	↓ (≈ 58.6% ± > 10%) SEM		Sinam et al. [112]
100 μM	24 h	↓ (≈ 66.0% ± > 10%)		Lee et al. [38]
100 μM	24 h	↓ (≈ 77.4% ± > 10%)		Tsuchida et al. [51]
100 μM	24 h	↓ (≈ 43.8% ± 5%)		Uozumi et al. [113]
100 μM	24 h	↓ (≈ 72.0% ± > 10%)		Van Pelt et al. [114]
100 μM	24 h	↓ (≈ 65.2% ± 5%)		Qui et al. [115]
100 μM	24 h	↓ (VC)		Reinoso‐Sánchez et al. [116]
100 μM	24 h	↓ (≈ 73.2% ± 5%)		Ozaki et al. [117]
100 μM	24 h	↓ (≈ 76.8% ± 5%)	↓ (≈ 69.4% ± 5%)	Lee et al. [118]
100 μM	24 h	↓ (≈ 47.4% ± > 10%)	↓ (≈ 61.2% ± > 10%)	Hur er al. [29]
100 μM	24 h	↓ (≈ 69.6% ± 5%)		Kim et al. [119]
100 μM	24 h	↓ (≈ 75.3% ± 5%)		Nguyen et al. [40]
100 μM	24 h	↓ (≈ 81.4% ± < 5%)		Han et al. [41]
100 μM	24 h	↓ (≈ 83.4% ± 5%)		Koo et al. [120]
100 μM	24 h	↓ (≈ 58.9% ± 5%)		Edwards et al. [S1]
100 μM	24 h	↓ (≈ 53.3% ± 5%)		Choi et al. [43]
100 μM	24 h	↓ (median ≈ 58.1% ±?%)		Kim et al. [S2]
100 μM	24 h	↓ (≈ 58.6% ± > 10%)	↓ (≈ 72.9% ± > 10%)	Kim et al. [39]
100 μM	24 h	↓ (≈ 88.8% ± < 5%)		Ko et al. [44]
100 μM	24 h	↓ (≈ 73.8% ± > 10%)	↓ (≈ 82.4% ± 5%)	Wang et al. [S3]
100 μM	24 h	↓ (≈ 23.3% ± > 10%)	↓ (≈ 64.5% ± 5%)	Kang et al. [S4]
100 μM	24 h	↓ (≈ 65.0% ± > 10%)		Zhou et al. [S5]
100 μM	24 h		↓ (≈ 60.0% ± > 10%)	Go et al. [S6]
100 μM	24 h	↓ (≈ 63.8% ± > 10%)		Lee et al. [S7]
100 μM	48 h	↓ (≈ 16.6% ± 5%)	↓ (≈ 14.2% ± 5%)	Cheon et al. [S8]
100 μM	48 h	↓ (≈ 46.2% ± 5%) SEM		Rossi et al. [S9]
100 μM	48 h	↓ (≈ 63.3% ± 5%) SEM		Han et al. [69]
100 μM	48 h	↓ (≈ 63.2% ± > 10%)		Chang et al. [S10]
100 μM	48 h	↓ (≈ 46.3% ± 5%)		Qui et al. [115]
100 μM	48 h	↓ (≈ 27.3% ± < 5%)		Li et al. [S11]
100 μM	48 h	↓ (≈ 59.3% ± > 10%)		Huang et al. [S12]
100 μM	48 h	↓ (≈ 74.1% ± > 10%)	↓ (≈ 61.4% ± > 10%)	Jang et al. [45]
100 μM	48 h	↓ (≈ 69.3% ± 5%)		Jeong et al. [S13]
100 μM	48 h	↓ (≈ 64.2% ± > 10%)	↓ (≈ 66.6% ± > 10%)	Chen et al. [S14]
100 μM	72 h	↓ (≈ 78.9% ± 5%)		Yoon et al. [S15]
100 μM	72 h	↓ (≈ 51.1% ± 5%)		Nakagawara et al. [S16]
100 μM	72 h	↓ (≈ 60.2% ± > 10%)		Lee et al. [46]
100 μM	96 h		↓ (≈ 66.6% ± > 10%)	Wang et al. [S17]
150 μM	6 h	↔ (≈ 98.7% ± 5%)		Gwag et al. [S18]
150 μM	12 h	↓ (≈ 83.9% ± 5%)		Gwag et al. [S18]
150 μM	18 h	↓ (≈ 77.7% ± 5%)		Gwag et al. [S18]
150 μM	24 h	↓ (≈ 74.0% ± 5%)		Gwag et al. [S18]
150 μM	24 h	↓ (≈ 77.7% ± 5%)	↓ (≈ 74.6% ± > 10%)	Bae et al. [S19]
200 μM	24 h	↓ (≈ 67.7% ± 5%)	↓ (≈ 73.6% ± > 10%)	Kwak et al. [S20]
200 μM	24 h	↓ (≈ 27.2% ± 5%)	↓ (≈ 42.6% ± > 10%)	Men et al. [33]
200 μM	24 h	↓ (≈ 34.5% ± > 10%)	↓ (≈ 34.9% ± > 10%)	Men et al. [48]
200 μM	24 h	↓ (≈ 43.0% ± 5%)	↓ (≈ 77.4% ± 5%)	Kim et al. [32]
200 μM	24 h	↓ (≈ 63.0% ± > 10%)		Wang et al. [S21]
200 μM	24 h	↓ (≈ 58.5% ± > 10%)		Jiang et al. [49]

*Note:* Columns are reported as raw values (if available) or as estimates (indicated by ≈) of treatment group expressed as a per cent of control ± the variability for the treated group. Variability is listed as SD unless noted with another reporting value (such as SEM). ? indicates the type of variability presented was unclear. ‘VC’ indicates visual confirmation was used to describe the effect of dexamethasone.

### 1.2 Aims and Scope of Review

Given the increasing prevalence of atrophy, sarcopenia and other muscle‐wasting related pathologies such as cachexia, proof‐of‐concept experiments investigating foundational levels of potential therapies for mechanistic insight are of high importance. Because dexamethasone‐induced myotube atrophy is a commonly used model, this work aims to (1) highlight the concentrations and durations commonly used in dexamethasone‐induced atrophy specifically used in C2C12 myotubes; (2) describe the effects of dexamethasone‐induced atrophy on myotube viability, phenotype and size, differentiation, atrophic related gene and protein expression, anabolic/protein synthetic signalling and metabolic response (specifically, related to mitochondrial content and function). As a clarification, though it is acknowledged that multiple other in vitro models of atrophy are used, dexamethasone‐treated C2C12 myotubes were the focus of the present report because they are among the most used models of mimicking skeletal muscle atrophy in vitro.

## 2 Methods

### 2.1 General Article Search and Inclusion Procedure

Primary literature was identified by authors first by searching PubMed using individual ‘dexamethasone AND atrophy AND C2C12’. Literature searches were repeated with specific atrophy‐related gene and/or protein names as related to each of the topics outlined above (and are listed as study outcomes in Table S1 and in each of the subsequent tables). Articles were also identified from reference lists of primary and review articles identified during the initial search based on relevance. Articles were included and summarized if (1) C2C12 myotubes were used as the indicated cell culture model of atrophy, (2) the article included a dexamethasone‐only group along with appropriate vehicle or true control and (3) the article assessed at least one of the related phenotypical or molecular outcomes of importance to the scope of the review. All references were screened for eligibility by a single reviewer (RAV) with curation completed using publication date of 3 January 2025. In addition to outlined search criteria, eight additional relevant articles were known by the authors prior to the initial search and were also included. As a cautionary note, it is acknowledged that given the increasingly prevalent use of the C2C12 model, it is likely this review will not be completely exhaustive. Included articles were then assessed for outcomes and raw data reported when available, or estimates of relative differences generated using ratio‐metric measurements of dexamethasone treatment versus relevant control. Importantly, although quality control and risk of bias are essential to guaranteeing the scientific veracity of each individual study, standards of reporting for in vitro experiments have changed substantially in the past two decades. Given the variability across reporting and the limited risk of bias assessment tools validated for in vitro findings, a bias assessment was not performed for each report. In‐text citations are provided when fewer than 20 references are appropriately citable. For other instances, please see supporting tables. Additional references exceeding 120 are located within the Supporting Information and referred to as S1, S2 and so forth. The in‐text table summaries focus on dexamethasone concentrations of 10 and 100 μM, which were the most frequently tested concentrations overall. To provide a general estimate of the effect of each concentration, average relative values of dexamethasone treatments versus control (control = 100) from all experiments are also included within the main text for dexamethasone concentrations of 10 and 100 μM for 24 h (presented as average ± SD). Importantly, these estimated average effects do not include data for protein expression only quantified by visual confirmation. Additionally, within some individual studies, multiple distinct experiments were conducted involving differing treatment conditions, such as varying treatment duration. For this reason, the number of studies at these concentrations, as well as individual experiments, is summarized in‐text. In the tables, these are recorded as separate entries, reflecting their unique experimental conditions.

## 3 Results

### 3.1 Search Results

**FIGURE 2:**
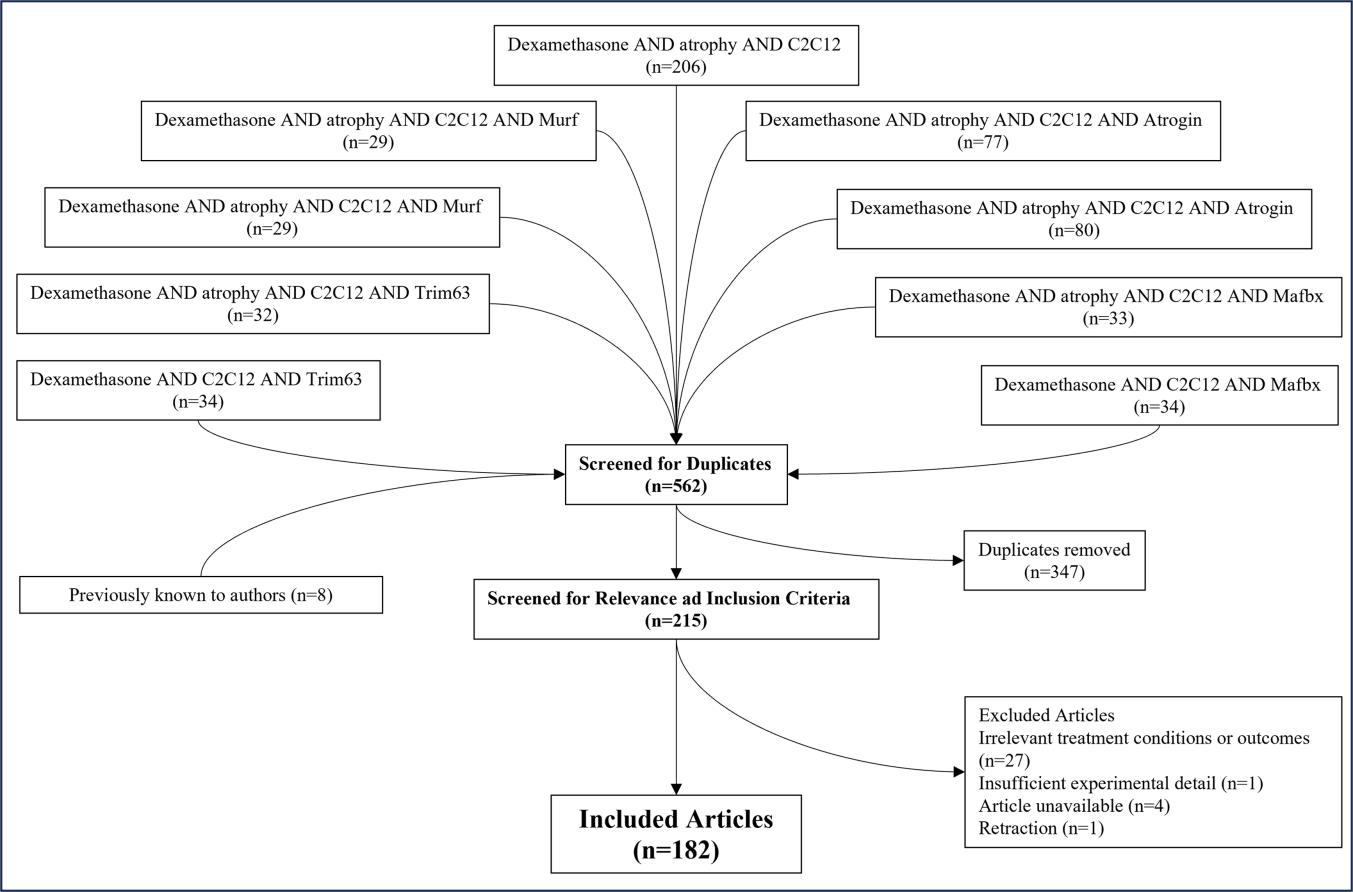
Flowchart of search strategy and results from PUBMED search for dexamethasone‐mediated atrophy using ‘dexamethasone AND atrophy AND C2C12’ with and without targets related to muscle atrophy as outlined in Section 2.

An initial historical literature search was performed up to the date of 3 January 2025 as described above, and a total of 562 results were identified. Following the removal of duplicate articles, 215 results were identified as of interest for inclusion. After the removal of articles that did not include relevant outcome measures, treatment conditions or sufficient experimental details, or were unavailable, a total of 182 articles were included. Details of the search results and article inclusion/exclusion are displayed in Figure 2.

### 3.2 Effect of Dexamethasone on Cultured Myotube Viability

We began by examining the effect of various dexamethasone concentrations on cultured myotube viability. In general, the majority of experiments defined ‘viability’ as relative value versus control for tetrazolium‐based assays (i.e., MTT, WST and cell counting kit (CCK) assay). Among the 11 studies identified that used 10 μM dexamethasone treatment [13, 18, 23, 24, 25, 26, 27, 28, 29, 30, 31], 9 out of the 13 unique experiments within these studies reported a reduction in myotube viability (total experimental average 77.2% ± 13.0% vs. control for 24‐h treatment). Likewise, in the 16 studies that utilized a dexamethasone treatment concentration of 100 μM [29, 30, 32, 33, 34, 35, 37, 38, 39, 40, 41, 42, 43, 44, 45, 46], 9 out of the 17 distinct experiments showed decreased myotube viability, and the remaining 8 experiments at this concentration found no change (total experimental average 81.6% ± 13.4% vs. control for 24‐h treatment). Please see Table 1 for the experimental conditions and outcomes for dexamethasone treatment on myotube viability.

### 3.3 Effect of Dexamethasone on Myotube Diameter and Fusion

Next, we examined the effect of various dexamethasone concentrations on myotube diameter/size and fusion index (generally defined as the ratio of nuclei within visible myotubes (with ≥ 3 nuclei) divided by total nuclei within a field of view). Among the 25 studies identified that utilized a 10 μM dexamethasone (Table 2), 24 of 26 unique experiments showed a decrease in myotube diameter/size (total experimental average 69.8% ± 7.5% vs. control for 24‐h treatment). Similarly, of the 37 studies that utilized a dexamethasone concentration of 100 μM, 37 of 39 unique experiments reported a decrease in myotube diameter/size (total experimental average 66.9% ± 14.7% vs. control for 24‐h treatment). Importantly, all six studies that treated with 10 μM dexamethasone [13, 23, 26, 88, 93, 95] (total experimental average 67.6% ± 5.3% vs. control for 24‐h treatment) and all nine that treated with 100 μM dexamethasone [29, 39, 45, 118] [S3, S4, S6, S8, S14] reported a reduction in fusion index (total experimental average 68.4% ± 8.4% vs. control for 24‐h treatment). Moreover, reduced myotube size was observed as early as 5 h following dexamethasone treatments and as late as following 6 days of dexamethasone exposure. Table 2 summarizes the effect of dexamethasone treatment across various experimental concentrations and treatment durations on myotube diameter and fusion.

### 3.4 Effect of Dexamethasone on Myotube Differentiation

We continued by examining the effect of dexamethasone at various concentrations on myotube differentiation, analysing both gene expression (mRNA) and protein levels. At a 10 μM dexamethasone concentration, 17 of 18 experiments from 16 identified studies observed a decrease (total experimental average 50.0% ± 17.2% vs. control for 24‐h treatment) in the corresponding myosin heavy chain (MHC) protein expression [13, 14, 23, 24, 25, 26, 31, 51, 87, 90, 92, 93, 94, 95, 98, 112]. At a higher concentration of 100 μM dexamethasone, three of five experiments from four identified studies showed decreased *Myh* gene expression, and 14 of 15 experiments from 14 studies reported a reduction (total experimental average 64.0% ± 15.1% vs. control for 24‐h treatment) in MHC protein [23, 45, 46, 61, 69, 111, 112] [S3, S6, S8, S22–S25]. In the case of myogenin, all three studies measuring mRNA expression reported a reduction, and all nine experiments from seven studies measuring MYOG protein level showed a decreased level following dexamethasone treatment at 10 μM (total experimental average 47.3% ± 21.8% vs. control for 24‐h treatment) in MYOG protein [14, 23, 24, 25, 31, 84, 89, 98, 112]. Similarly, at 100 μM dexamethasone, two of three experiments found a reduction in myogenin mRNA expression, and all five experiments that examined MYOG protein levels also reported a decrease (total experimental average 32.9% ± 28.2% vs. control for 24‐h treatment) [23, 38, 40, 45, 61, 112] [S8, S23]. Additionally, following 10 μM dexamethasone treatment, all three experiments that assessed mRNA expression of myoblast determination protein (*Myod*) reported a decrease, as did all six experiments from four studies that measured protein expression, which also showed a reduction (total experimental average 61.8% ± 5.3% vs. control in MYOD protein for 24‐h treatment) [23, 25, 58, 84, 89, 96]. At 100 μM dexamethasone, three of five experiments on *Myod* mRNA levels reported a decrease, and three of four experiments on MYOD protein expression also showed a decline (total experimental average 68.4% ± N/A% vs. control for 24‐h treatment) in MYOD protein [40, 44, 45, 46, 58, 118] [S4, S8]. Please see Table S2 for experimental conditions and outcomes for dexamethasone treatment on myotube differentiation.

### 3.5 Effect of Dexamethasone on Atrophic Signalling

Next, we investigated the effect of dexamethasone on atrophic signalling in myotubes (Table S3). Myostatin (*Mstn*) expression consistently increased (total experimental average 411.1% ± 117.0% vs. control for 24‐h treatment), as reported in all eight studies that utilized 10 μM dexamethasone treatment [26, 69, 84, 93, 96] [S26–S28]. Likewise, four of five studies reported an upregulation (total experimental average 282.1% ± 220.8% vs. control for 24‐h treatment) of *Mstn* following 100 μM treatment [42, 43, 44, 69] [S29], and all five experiments that utilized 100 μM dexamethasone showed an increase (total experimental average 158.2% ± 7.5% vs. control for 24‐h treatment) in MSTN protein [42] [S3, S6, S29, S30]. For muscle RING‐finger protein‐1 (*Murf1* or *Trim63*), among the 19 studies that reported on gene expression following 10 μM dexamethasone, 21 of 30 distinct studies reported an increase (total experimental average 304.7% ± 191.1% vs. control for 24‐h treatment), while the remaining reported no change (Table S3). At a higher concentration of 100 μM, 29 distinct experiments from 26 studies reported an increase (total experimental average 334.7% ± 167.5% vs. control for 24‐h treatment) in *Murf1/Trim63* gene expression (Table S3). The associated MURF1 protein showed a similar trend with elevated levels observed in 16 of 17 experiments at 10 μM (total experimental average 224.2% ± 154.7% vs. control for 24‐h treatment) and in 30 of 31 experiments at 100 μM across 29 studies (total experimental average 276.7% ± 186.0% vs. control for 24‐h treatment) (Table S3). Furthermore, the expression of the muscle atrophy F‐box (*Mafbx* or *Fbx32*) was upregulated (total experimental average 458.2% ± 349.9% vs. control for 24‐h treatment) in 24 of 27 experiments from 18 studies at 10 μM [18, 25, 26, 30, 51, 69, 84, 87, 89, 90, 91, 96] [S26, S28, S31–S34] and in 31 of 35 experiments from 30 studies at 100 μM (total experimental average 495.9% ± 321.7% vs. control for 24‐h treatment for 24‐h treatment) (Table S3). At the protein level, Atrogin‐1 expression also showed increased (total experimental average 269.1% ± 136.6% vs. control for 24‐h treatment) in all 19 experiments at 10 μM and in 33 of 34 experiments at 100 μM (total experimental average 351.7% ± 268.4% vs. control for 24‐h treatment) (Table S3). In addition, we found treatment with dexamethasone at 1 μM for 24 h was also an exceedingly common concentration used by several experiments that assessed indicators of atrophic signalling [12, 58, 59, 61, 62, 63, 64, 66, 68] [S7, S35–S42]. Like experiments that used 10 and 100 μM, these studies also demonstrated consistent elevations in *Murf1* mRNA (total experimental average 268.7% ± 147.4% vs. control for 24‐h treatment) and MURF1 protein (total experimental average 222.3% ± 121.9% vs. control for 24‐h treatment), as well as increases in *Mafbx* mRNA expression (total experimental average 314.3% ± 86.8% vs. control for 24‐h treatment) and Atrogin‐1 protein expression (total experimental average 238.7% ± 115.4% vs. control for 24‐h treatment) (Table S3). Collectively, these findings demonstrate the consistency of dexamethasone‐mediated atrophic signalling.

### 3.6 Effect of Dexamethasone on Foxo1/3 Signalling

We next examined the effect of exposing myotubes to various dexamethasone concentrations on forkhead box protein O1 (FOXO1) and/or forkhead box protein O3 (FOXO3) signalling. For *Foxo1*, gene expression and relative amount of FOXO1 protein were inconsistent across the studies that assessed total FOXO1 (Table S4). However, both experiments that assessed phosphorylated FOXO1 (inactive) following 10 μM dexamethasone treatment reported a decrease, indicating increased activation of active FOXO1 [30, 31]. For experiments that utilized 100 μM dexamethasone treatment, four of five experiments from five studies reported a reduction in phosphorylated FOXO1 (total experimental average 46.3% ± 6.6% vs. control for 24‐h treatment) [38] [S23, S30, S43, S44]. In the case of Foxo3, gene expression was consistently upregulated (total experimental average 161.0% ± 39.0% vs. control for 24‐h treatment) in all seven experiments from three studies at 10 μM [25, 30, 51], as were protein levels of FOXO3 in all 11 experiments that utilized 10 μM (total experimental average 260.0% ± 173.4% vs. control for 24‐h treatment) [13, 14, 18, 25, 30, 51, 87, 90, 91, 92, 93]. Similar observations were made at 100 μM, with four of five experiments showing increased (total experimental average 246.7% ± 214.4% vs. control for 24‐h treatment) Foxo3 gene expression [30, 35, 43] [S13, S24] and all five revealing increased protein expression (total experimental average 256.9% ± 158.4% vs. control for 24‐h treatment) [30, 37, 38, 42, 61]. Moreover, phosphorylated FOXO3 (inactive) levels were reported to decrease (total experimental average 38.6% ± 17.7% vs. control for 24‐h treatment) in all 11 studies at 10 μM [18, 28, 31, 51, 87, 89, 90, 91, 92, 93] [S36] and in 10 of 14 experiments at 100 μM (total experimental average 93.3% ± 68.7% vs. control for 24‐h treatment) [37, 38, 40, 43, 44, 45, 61] [S3, S10, S13, S14, S23, S30, S44], indicating altered FOXO3 activity following dexamethasone treatment. The effects of dexamethasone on myotube FOXO expression and phosphorylation are presented in Table S4. As a special note, a study worthy of further mention in the context of dexamethasone‐mediated regulation of FOXO signalling is that by Cid‐Diaz et al., who explored the expression and phosphorylation patterns of FOXO1, FOXO3 and FOXO4 across multiple concentrations of dexamethasone treatment [61]. The report also complemented these efforts by assessing several other indicators of myotube formation, atrophy‐related signalling and protein synthesis [61].

### 3.7 Effect of Dexamethasone on Anabolic Signalling

We next examined the effect of dexamethasone on myotube anabolic signalling, analysing key signalling proteins and markers of protein synthesis. The most common finding of reduced anabolic signalling following dexamethasone treatment was a decrease in phosphorylated Akt, a central component of insulin signalling (Table S5). A reduction (total experimental average 58.4% ± 18.1% vs. control for 24‐h treatment) in pAkt was observed in 13 of 21 experiments across 13 studies at 10 μM dexamethasone [13, 14, 18, 26, 28, 51, 89, 90, 91, 92, 94, 96] [S28] and in 18 of 21 experiments from 21 studies at 100 μM (total experimental average 64.2% ± 22.5% vs. control for 24‐h treatment) (Table S5). The consistent observation of reduced Akt phosphorylation suggests disruption in anabolic signalling. Similarly, phosphorylated mTOR levels were reduced (total experimental average 67.6% ± 32.3% vs. control for 24‐h treatment) in four of five experiments from four studies at 10 μM [13, 14, 26, 89] and in 15 of 16 experiments from 16 studies at 100 μM (total experimental average 59.8% ± 19.8% vs. control for 24‐h treatment) [29, 35, 37, 39, 42, 45, 119] [S1, S3, S5, S8, S12–S14, S23, S44], suggesting impaired protein synthesis and growth. This phenotype is further corroborated by reports of a reduction in phosphorylated P70s6k, a protein kinase involved in translation initiation and protein synthesis. At 10 μM, all five experiments reported a decrease (total experimental average 67.9% ± 25.2% vs. control for 24‐h treatment) [13, 14, 26, 51, 95], as did 9 of 10 experiments at 100 μM (total experimental average 59.3% ± 46.1% vs. control for 24‐h treatment) [29, 37, 39, 40, 44, 45, 119] [S1, S8, S12]. Furthermore, following treatment with dexamethasone at 100 μM, six of eight experiments reported a reduction (total experimental average 74.1% ± 28.8% vs. control for 24‐h treatment) in phosphorylated eukaryotic translation initiation factor 4E‐binding protein 1 (p‐4EBP1) [29, 37, 39, 40, 44, 45] [S1, S8], reinforcing indications of inhibited protein translation. Additionally, the both studies at 100 μM that assessed phosphorylated ribosomal protein S6 (p‐RPS6), a ribosomal component crucial to protein synthesis, reported a decrease [37] [S1]. Lastly, puromycin incorporation, a direct marker of protein synthesis, was decreased in experiments conducted at 10 [91] and 100 μM [S1]. Collectively, these observations demonstrate that dexamethasone treatment consistently reduces anabolic signalling in cultured C2C12 myotubes (Table S5).

### 3.8 Effect of Dexamethasone‐Mediated Atrophy on Myotube Mitochondrial Content and Function

Lastly, we examined the effect of dexamethasone‐mediated atrophy on myotube mitochondrial content and function given mitochondrial dysfunction is a key feature of muscle atrophy [10]. We began by first examining the effect of dexamethasone‐mediated atrophy on signalling of mitochondrial biogenesis by reporting on findings at both gene and protein expressional levels. The reviewed studies used dexamethasone concentrations ranging from 1 to 200 μM, with the most common concentration being 100 μM. Five of eight studies reported an increase in phosphorylated AMP‐activated protein kinase (AMPK) [12, 34, 35, 42, 49, 101] [S1, S45], suggesting stress‐induced activation of energy pathways. However, despite this activation, additional downstream or synergistic regulators of metabolism were largely impaired, suggesting reduced mitochondrial biogenesis. Specifically, all studies that assessed the effect of dexamethasone on myotube sirtuin 1 (SIRT1) mRNA and/or protein expression showed a reduction in SIRT1 [28, 33, 48, 92] [S8], a deacetylase that is a known positive regulator of peroxisome proliferator‐activated receptor‐gamma coactivator 1 alpha (*Ppargc1a*/PGC‐1α) through deacetylation [S46]. The reduction in SIRT1 was paralleled by reductions in PGC‐1α with six of seven studies observing a reduction in PGC‐1α protein levels [28, 35, 48, 87, 92] [S1, S8]. Not surprisingly, the reduction in PGC‐1α was associated with decreased levels of nuclear respiratory factor 1 (*Nrf1*/NRF1) and mitochondrial transcription factor A (*Tfam*/TFAM), both of which are normally activated by PGC‐1α to regulate mitochondrial function. Collectively, these findings indicate that despite AMPK activation, dexamethasone treatment disrupts mitochondrial biogenesis (Table S6).

Given the previous results indicating reduced mitochondrial biogenesis in dexamethasone‐treated myotubes, we examined mitochondrial content and function. Again, dexamethasone was associated with reduced mitochondrial content with seven of seven studies reporting reduced mitochondrial staining [26, 28, 32, 34, 40, 44, 92]. Reduced mitochondrial content was also associated with reduced mitochondrial function including reduced ATP production/content in eight of eight studies [12, 26, 28, 32, 34, 40, 44, 92] and reduced oxygen consumption in five of five studies [12, 21, 28, 34, 80]. Taken together, these results indicate that dexamethasone treatment inhibits mitochondrial function (Table S7) as well as the biogenesis of new mitochondria. Like measures of reduced protein synthesis and increased protein breakdown, indicators of consistently reduced mitochondrial content and function are of special importance because mitochondrial dysfunction is a common characteristic of muscle‐related pathology. Moreover, in the context of dexamethasone‐mediated atrophy, it has been shown in vivo that mice treated with dexamethasone display reductions in mitochondrial function before loss of muscle mass [12]. In fact, Liu et al. concluded that reductions in mitochondrial function and ATP abundance promote atrophic signalling in a positive feedback manner in a way that precedes muscle loss [12]. Such observations implicate mitochondrial dysfunction as a potentially causal agent in the development of atrophy and are worthy of further exploration.

## 4 Discussion and Limitations

Dexamethasone‐treated C2C12 myotubes is a commonly used method for studying various aspects of muscle pathology, frequently used as models of atrophy, sarcopenia and myopathy. This review highlights many of the features of the dexamethasone‐treated C2C12 myotube model including several consistent features. For example, the reduction of myotube viability, size and fusion index in myotubes treated with dexamethasone was consistent. Similarly, reduced anabolic and differentiation signalling was also consistently observed in dexamethasone‐treated C2C12 myotubes, while increased atrophy‐related signalling was also consistently observed. Additionally, most of these findings appeared to be dose‐dependent (exaggerated within increasing dexamethasone concentration) and time‐dependent (exaggerated within increasing treatment duration); however, a limitation of this work was the lack of meta‐analysis due to the high degree of variability of experimental reporting for sample size/replicates and so forth. Additionally, it should be noted that the stage of differentiation at which cells receive dexamethasone is likely important, as at least one report demonstrated that the administration of dexamethasone at the early myoblast state improved differentiation [69]. Moreover, the type of vehicle used for the delivery of dexamethasone might also contribute to the effect of dexamethasone within each experiment.

Another interesting result that emerged is the reduction of mitochondrial function, which appears to be a consistent feature of atrophy in the dexamethasone‐treated C2C12 myotube model; however, the exact causal mechanism remains unclear. Liu et al. noted that reductions in ATP levels and mitochondrial function may precede atrophic signalling in a positive feedback manner [12], though the cascade of events that lead to reduced mitochondrial content remain unclear. It could be that dexamethasone treatment directly affects mitochondrial function by increasing levels of oxidative stress [13], reduced mitochondrial membrane potential [14] or a combination thereof could result in reduced mitochondrial function. Conversely, it could be that dexamethasone treatment works indirectly by reducing anabolic signalling such as mTORC1 (which was a consistent finding within this report), which is known to collaborate with YY1 to regulate mitochondrial content and function [16]. In fact, Li et al. showed that dexamethasone treatment reduced YY1 expression in mouse skeletal muscle [15], though the dependence of dexamethasone‐mediated atrophy on reduced YY1 levels is unclear and speculative.

Next, the most inconsistent finding of the present report is the influence of dexamethasone treatment on FOXO signalling. It is well recognized that FOXO signalling is highly involved in mediating skeletal muscle atrophy (many of the details are described elsewhere [S47]). However, even the latest review on the subject acknowledges a shortcoming of current observations in describing the full mechanisms [S47]. One contributing factor may be the inconsistent measurements carried out across related experiments. For example, changes in mRNA expression are not always consistent with levels of the translated protein, nor does it account for posttranslational modifications that influence activity. Additionally, some reports explored cytosolic versus nuclear fractions, which are also likely to contribute different information than the aforementioned mRNA and protein data. Of the most exhaustive reports we identified on dexamethasone‐treated C2C12 atrophy, Cid‐Diaz et al. measured the expressional and phosphorylation patterns of FOXO1, FOXO3 and FOXO4 across multiple concentrations of dexamethasone treatment [61]. Indeed, the effect of dexamethasone on FOXO signalling is likely to be dependent on not only dexamethasone concentration but also the location of the posttranslational modifications [61]. That said, the report does show consistent reductions in the phosphorylation of FOXO1 at both T24 and S256, which would correspond to lower Akt signalling and increased FOXO1 translation to the nucleus (with greater activity promoting atrophy‐related signalling) [61]. Additionally, the effect of dexamethasone on FOXO3 activity also seems to be dependent on both concentration and treatment duration, though it appears lower phosphorylation of FOXO3 is consistently observed for higher concentrations of dexamethasone (i.e., 100–200 μM for 24–48 h).

In addition to the limitations and considerations mentioned above in dexamethasone‐treated C2C12 myotubes, several other limitations exist. For instance, we did not exhaustively describe every potential outcome associated with atrophy. For example, dexamethasone likely alters other contributing pathways such as autophagic signalling or the E3 ubiquitin‐proteasome system. Additionally, the present report only described the effect of dexamethasone on myotube atrophy, yet numerous other stimuli can be used to induce atrophy in C2C12 myotubes. For example, treatment with inflammatory cytokines such as IL6 or TNFα, which mechanistically function through several additional molecular targets such as NFκB, or treatment with proteins within the TGF‐β superfamily, which function via activin receptors, are also commonly used and result in decreased myotube size and protein aggregation. Similarly, it is acknowledged that several other cell models including L6 rat myocytes, primary human skeletal muscle cells (HSkMC), induced pluripotent stem cells (iPSC) and, of course, primary cells isolated from sources such as rodents or human subjects. Thus, it should be acknowledged that C2C12 cells are not the only viable in model to mimic skeletal muscle pathology. Lastly, while it is anticipated that observations using the dexamethasone‐mediated atrophy in C2C12 map to disease in vivo, it should be acknowledged that important differences between in vitro systems may exist. For example, differences in media composition and nutrient availability during the summarized experiments (i.e., high vs. low glucose media content, amino acid availability and type and quantity of serum) and what is present during disease in vivo is likely quite different. Additionally, sex and age differences are characteristics of in vivo systems that likely influence outcomes associate with atrophy, but are not reliably explorable using in vitro models like the C2C12 cell line. Thus, these limitations should be considered when generalizing findings to in vivo systems.

## 5 Concluding Remarks

Skeletal muscle is a highly consequential tissue in the context of well‐being, and it plays important roles in the pathophysiology of many diseases. In vitro models of skeletal muscle physiology are useful tools for the study of muscle‐related pathologies. In this report, we summarized many of the characteristics common to the dexamethasone‐mediated C2C12 model of skeletal muscle atrophy. In general, C2C12 myotubes treated with dexamethasone displayed reduced myotube size and formation, which is accompanied by reduced signalling associated with myotube differentiation and protein synthesis. Moreover, dexamethasone‐treated myotubes consistently displayed increased proteolytic expressional profiles (especially Atrogin‐1 and MuRF‐1). Finally, dexamethasone also consistently reduced mitochondrial function and content, which was supported by reduced signalling associated with mitochondrial biogenesis. Together, these findings demonstrate a striking consistency of the effect of dexamethasone treatment on changes in myotube physiology across a wide range of treatment concentrations and durations, changes that have been previously associated with several muscle pathologies. However, given that the development of pathologies such as atrophy and sarcopenia are dynamic processes, the generalizability of in vitro findings should be interpreted with caution. Similarly, primary research utilizing in vitro models such as the dexamethasone‐mediated C2C12 models should highlight key differences between the clinical features of the disease and the in vitro model.

## Funding

This work was supported by the Department of Health and Human Performance within the Congdon School of Health Sciences. Additional support was provided by the Congdon School of Health Sciences Summer Undergraduate Research Fellowship (SURF).

## Ethics Statement

This research falls outside human or animal studies, and institutional ethical approval was not required.

## Consent

The authors have nothing to report.

## Conflicts of Interest

The authors declare no conflicts of interest.

## Supporting information


**Table S1:** **Description of assessed outcomes per PRISMA guidelines.** Abbreviations: AMPK, AMP‐activated protein kinase; p‐EBP1, eukaryotic translation initiation factor 4E‐binding protein 1; Foxo1, Forkhead box protein 01 (mRNA); FOXO1, Forkhead box protein 01 (protein); Foxo3, Forkhead box protein 03 (mRNA); FOXO3, Forkhead box protein 01 (protein); Mstn, myostatin (mRNA); Mafbx or Fbx32, muscle atrophy x box (mRNA); Murf1 or Trim63, Muscle RING‐finger protein‐1 (mRNA); MURF1, Muscle RING‐finger protein‐1 (protein); Myh, myosin heavy chain (mRNA); MHC, myosin heavy chain (protein); Myod, myoblast determination protein (mRNA); MYOD, myoblast determination protein (protein); Myog, myogenin (mRNA); MYOG, myogenin (protein); p‐mTOR, mechanistic/mammalian target of rapamycin; Nrf1, nuclear respiratory factor 1 (mRNA); NRF1, nuclear respiratory factor 1 (protein); p‐P70s6k, phosphorylated ribosomal protein S6; Ppargc1a, peroxisome proliferator‐activated receptor gamma coactivator 1 alpha (mRNA); PGC‐1α, peroxisome proliferator‐activated receptor gamma coactivator 1 alpha (protein); Sirt1, Sirtuin 1 (mRNA); SIRT1, Sirtuin 1 (protein); Tfam, mitochondrial transcription factor A (mRNA); TFAM, mitochondrial transcription factor A (protein).
**Table S2: Effect of dexamethasone on myotube differentiation.** Note: Column are reported as raw values (if available) or as estimates (indicated by ≈) of treatment group expressed as a percent of control ± the variability for the treated group. Variability is listed as SD unless noted with another reporting value (such as SEM).? indicates the type of variability presented was unclear. “VC” indicates visual confirmation was used to describe the effect of dexamethasone. “Varied MT” indicates that multiple targets were assessed (transcript or isotope variants). **Abbreviations**: Myh, myosin heavy chain (mRNA); MHC, myosin heavy chain (protein); Myod, myoblast determination protein (mRNA); MYOD, myoblast determination protein (protein); Myog, myogenin (mRNA); MYOG, myogenin (protein).
**Table S3: Effect of dexamethasone on atrophic signalling.** Note: Column are reported as raw values (if available) or as estimates (indicated by ≈) of treatment group expressed as a percent of control ± the variability for the treated group. Variability is listed as SD unless noted with another reporting value (such as SEM).? indicates the type of variability presented was unclear. “VC” indicates visual confirmation was used to describe the effect of dexamethasone. “Varied MT” indicates that multiple targets were assessed (transcript or isotope variants). “NM” indicates that relative assessments were not measurable. **Abbreviations**: Mstn, myostatin (mRNA); Mafbx or Fbx32, muscle atrophy x box (mRNA); Murf1 or Trim63, Muscle RING‐finger protein‐1 (mRNA); MURF1, Muscle RING‐finger protein‐1 (protein).
**Table S4: Effect of dexamethasone on Foxo1/3 signalling.** Note: Column are reported as raw values (if available) or as estimates (indicated by ≈) of treatment group expressed as a percent of control ± the variability for the treated group. Variability is listed as SD unless noted with another reporting value (such as SEM).? indicates the type of variability presented was unclear. “VC” indicates visual confirmation was used to describe the effect of dexamethasone. “Varied MT” indicates that multiple targets were assessed (transcript or isotope variants). “NM” indicates that relative assessments were *not measurable*. **Abbreviations**: Cyt, cytosolic; Foxo1, Forkhead box protein 01 (mRNA); FOXO1, Forkhead box protein 01 (protein); Foxo3, Forkhead box protein 03 (mRNA); FOXO3, Forkhead box protein 01 (protein); Nuc, nuclear.
**Table S5: Effect of dexamethasone on anabolic signalling.** Note: Column are reported as raw values (if available) or as estimates (indicated by ≈) of treatment group expressed as a percent of control ± the variability for the treated group. Variability is listed as SD unless noted with another reporting value (such as SEM).? indicates the type of variability presented was unclear. “VC” indicates visual confirmation was used to describe the effect of dexamethasone. “Varied MT” indicates that multiple targets were assessed (transcript or isotope variants). “NM” indicates that relative assessments were not measurable. **Abbreviations**: p‐EBP1, eukaryotic translation initiation factor 4E‐binding protein 1; p‐mTOR, mechanistic/mammalian target of rapamycin; phosphorylated ribosomal protein S6.
**Table S6: Effect of dexamethasone‐mediated atrophy on myotube mitochondrial biogenesis.** Note: Column are reported as raw values (if available) or as estimates (indicated by ≈) of treatment group expressed as a percent of control ± the variability for the treated group. Variability is listed as SD unless noted with another reporting value (such as SEM).? indicates the type of variability presented was unclear. “VC” indicates visual confirmation was used to describe the effect of dexamethasone. “Varied MT” indicates that multiple targets were assessed (transcript or isotope variants). “NM” indicates that relative assessments were *not measurable*. **Abbreviations**: AMPK, AMP‐activated protein kinase; Nrf1, nuclear respiratory factor 1 (mRNA); NRF1, nuclear respiratory factor 1 (protein); Ppargc1a, peroxisome proliferator‐activated receptor gamma coactivator 1 alpha (mRNA); PGC‐1α, peroxisome proliferator‐activated receptor gamma coactivator 1 alpha (protein); Sirt1, Sirtuin 1 (mRNA); SIRT1, Sirtuin 1 (protein); Tfam, mitochondrial transcription factor A (mRNA); TFAM, mitochondrial transcription factor A (protein).
**Table S7: Effect of dexamethasone‐mediated atrophy on myotube mitochondrial content and function.** Note: Column are reported as raw values (if available) or as estimates (indicated by ≈) of treatment group expressed as a percent of control ± the variability for the treated group. Variability is listed as SD unless noted with another reporting value (such as SEM). ? indicates the type of variability presented was unclear. “VC” indicates visual confirmation was used to describe the effect of dexamethasone. “Varied MT” indicates that multiple targets were assessed (transcript or isotope variants). “NM” indicates that relative assessments were *not measurable*. **Abbreviations**: ATP, Adenosine triphosphate; Ox Phos, oxidative phosphorylation.

**Data S1:** Supporting Information.

## Data Availability

This article is a review of literature and did not report any original data.
