# Supplementary material for: A Systematic Review and User Reference of Phenotypic and Molecular Characteristics of Dexamethasone‐Mediated C2C12 Muscle Atrophy
Source: J Cachexia Sarcopenia Muscle. 2026 Mar 13;17(2):e70127. doi: 10.1002/jcsm.70127 (PMC13140628; doi:10.1002/jcsm.70127)
Supplement: Supplementary file 2 — Data S1: Supporting Information. [file JCSM-17-e70127-s002.docx]

**A systematic review and user reference of phenotypic and molecular characteristics of dexamethasone-mediated C2C12 muscle atrophy**

**SUPPLEMENTAL MATERIALS**

Alexa J. Klein^1^ and Roger A. Vaughan^1^*

*Author Correspondence:

Email: [rvaughan@highpoint.edu](mailto:rvaughan@highpoint.edu)

Address: One University Parkway, High Point, NC 27262-3598

Phone: 336-841-9688

ORIC ID: 0000-0002-4593-8807

*^1^ Department of Health and Human Performance, High Point University, High Point, NC*

**Supplemental Tables**

***Table s1 Description of assessed outcomes per PRISMA guidelines.***

| Outcome | Type | Table Reported |
| --- | --- | --- |
| Viability | Viability | 1 |
| Diameter/Size | Phenotype | 2 |
| Fusion Index | Phenotype | 2 |
| Assessed Outcomes | Assessed Outcomes | s1 |
| Myh | mRNA | s2 |
| MHC | Protein | s2 |
| Myog (RNA) | mRNA | s2 |
| MYOG | Protein | s2 |
| Myod (RNA) | mRNA | s2 |
| MYOD | Protein | s2 |
| Mstn | mRNA | s3 |
| Myostatin | Protein | s3 |
| Murf1 (Trim63) | mRNA | s3 |
| MURF1 | Protein | s3 |
| Mafbx | mRNA | s3 |
| Atrogin-1 | Protein | s3 |
| Foxo1 (mRNA) | mRNA | s4 |
| FOXO1 (protein) | Protein | s4 |
| p-FOXO1 | Protein (inactive) | s4 |
| Foxo3 (mRNA) | mRNA | s4 |
| FOXO3 (protein) | Protein | s4 |
| p-FOXO3 | Protein (inactive) | s4 |
| p-Akt | Protein (active) | s5 |
| p-mTOR | Protein (active) | s5 |
| p-P70s6k | Protein (active) | s5 |
| p-4EBP1 | Protein (active) | s5 |
| p-RPS6 | Protein (active) | s5 |
| puromycin incorporation | Metabolic Process | s5 |
| pAMPK | Protein (active) | s6 |
| Ppargc1a | mRNA | s6 |
| PGC1α | Protein | s6 |
| Nrf1 | mRNA | s6 |
| NRF1 | Protein | s6 |
| Tfam | mRNA | s6 |
| TFAM | Protein | s6 |
| Sirt1 | mRNA | s6 |
| SIRT1 | Protein | s6 |
| Mitochondrial Staining | Mitochondrial Content | s7 |
| ATP | Mitochondrial Function | s7 |
| O2 Consumption | Mitochondrial Function | s7 |
| Ox Phos | Mitochondrial Function | s7 |

***Table s2 Effect of dexamethasone on myotube differentiation***

| Concentration | Duration | *Myh* | MHC | *Myog* (RNA) | MYOG | *Myod* (RNA) | MYOD | Reference |
| --- | --- | --- | --- | --- | --- | --- | --- | --- |
| 0.01µM | 48hr |  | ↔ (≈92.6 ±5%) |  | ↔ (≈93.0 ±5%) |  |  | Ma et al. 2014 [23] |
| 0.01µM | 192hr |  |  | **↓** (≈87.1 ±5%) SEM |  | **↓** (≈79.4 ±5%) SEM |  | Kim et al. 2016 [54] |
| 0.05µM | 24hr |  | ↓ (≈59.3 ±5%) |  | ↔ (≈91.5 ±5%) |  |  | Cid-Diaz et al. 2017 [61] |
| 0.1µM | 24hr |  |  |  |  |  | ↓ VC | Son et al. 2015 [58] |
| 0.1µM | 48hr |  | ↔ (≈85.3 ±5%) |  | ↔ (≈83.7 ±5%) |  |  | Ma et al. 2014 [23] |
| 0.1µM | 192hr |  |  | **↓** (≈38.4 ±5%) SEM |  | **↓** (≈17.9 ±5%) SEM |  | Kim et al. 2016 [54] |
| 0.5µM | 24hr |  | ↓ (≈61.0 ±5%) |  | ↓ (≈72.8 ±5%) |  |  | Cid-Diaz et al. 2017 [61] |
| 1µM | 6hr |  |  |  |  |  | ↓ VC | Son et al. 2015 [58] |
| 1µM | 6hr |  |  | ↓ (≈42.4 ±5%) SEM |  | ↓ (≈33.3 ±5%) SEM |  | Hong et al. 2019 [s48] |
| 1µM | 12hr |  |  |  |  |  | ↓ VC | Son et al. 2015 [58] |
| 1µM | 12hr | ↔ Varied (MT) | ↓ (≈41.8 ±<5%) |  |  |  |  | Yeon et al. 2020 [s49] |
| 1µM | 24hr |  |  |  |  |  | ↓ VC | Son et al. 2015 [58] |
| 1µM | 24hr |  | ↓ (≈42.3 ±5%) |  | ↓ (≈66.1 ±5%) |  |  | Cid-Diaz et al. 2017 [61] |
| 1µM | 24hr |  | ↓ (≈65.6 ±>10%) |  |  |  |  | Yoshioka et al. 2023 [s42] |
| 1µM | 24hr |  | ↓ (≈45.7 ±>10%) | ` |  |  |  | Yoshioka et al. 2023 [66] |
| 1µM | 24hr |  | ↓ (≈65.5 ±>10%) |  |  |  |  | Hsieh et al. 2019 [68] |
| 1µM | 48hr |  |  |  |  | ↔ (≈127.2 ±5%) SEM |  | Singh et al. 2017 [70] |
| 1µM | 48hr |  | ↓ (≈58.5 ±5%) |  | ↓ (≈58.5 ±5%) |  |  | Ma et al. 2014 [23] |
| 1µM | 72hr | ↔ Varied (MT) |  |  |  |  |  | Archer-Lahlou et al. 2018 [76] |
| 1µM | 48hr |  |  |  |  |  | ↓ VC | Son et al. 2015 [58] |
| 1µM | 48hr |  |  | **↓** (≈25.0 ±<5%) | **↓** (≈25.8 ±<5%) |  |  | Adhikary et al. 2019 [s50] |
| 1µM | 48hr |  | **↓** (50 ±NR%) |  |  |  |  | Han et al. 2020 [71] |
| 1µM | 48hr |  | ↓ (≈59.1 ±5%) |  |  |  | ↓ (≈56.8 ±5%) | Salvadori et al. 2024 [s51] |
| 1µM | 192hr |  |  | **↓** (≈28.2 ±5%) SEM |  | **↓** (≈10.2 ±5%) SEM |  | Kim et al. 2016 [54] |
| 5µM | 24hr |  | ↓ VC | ↓ (≈57.8 ±5%) |  | ↓ (≈57.8 ±5%) |  | Lee et al. 2022 [21] |
| 5µM | 24hr |  | ↓ (≈89.4 ±5%) |  | ↓ (≈92.9 ±5%) |  |  | Kim et al. 2022 [22] |
| 5µM | 24hr |  | ↔ Varied (MT) |  |  |  |  | Kim et al. 2022 [79] |
| 5µM | 24hr |  | ↓ VC | ↓ (≈66.6 ±>10%) |  | ↓ (≈66.6 ±>10%) |  | Lee et al. 2021 [80] |
| 5µM | 24hr |  |  |  |  | ↓ (≈66.6 ±5%) |  | Son et al. 2017 [81] |
| 5µM | 48hr | ↓ Varied (MT) | ↓ VC |  |  |  |  | Gan et al. 2022 [83] |
| 10µM | 12hr |  | ↔ (≈93.2 ±>10%) |  |  |  |  | Tsuchida et al. 2017 [51] |
| 10µM | 12hr |  |  |  | ↓ (≈20 ±?%) |  | ↓ (≈68.4 ±>10%) | Ma et al. 2014 [23] |
| 10µM | 24hr |  | ↓ (≈30.7 ±5%) SEM |  | ↓ (≈78.9 ±5%) SEM |  |  | Sinam et al 2022 [112] |
| 10µM | 24hr |  |  | ↓ (≈16.6 ±>10%) |  | ↓ (≈50.0 ±>10%) |  | Lee et al. 2022 [84] |
| 10µM | 24hr |  |  |  | ↓ (≈33.3 ±>10%) |  | ↓ (≈65.6 ±>10%) | Ma et al. 2014 [23] |
| 10µM | 24hr |  |  |  |  |  | ↓ VC | Son et al. 2015 [58] |
| 10µM | 24hr |  | ↓ (≈70.7 ±>10%) |  |  |  |  | Tsuchida et al. 2017 [51] |
| 10µM | 24hr |  | ↓ (≈33.8 ±>10%) |  |  |  |  | Ulla et al. 2021 [87] |
| 10µM | 24hr |  |  | ↓ (≈80.9 ±>10%) |  | ↓ (≈65.8 ±>10%) |  | Kimira et al. 2023 [89] |
| 10µM | 24hr |  | ↓ (≈37.5 ±<5%) SEM |  | ↓ (≈45.0 ±<5%) SEM |  |  | Amarasiri et al. 2024 [24] |
| 10µM | 24hr |  | ↓ (≈45.6 ±5%) | ↓ (≈44.0 ±5%) | ↓ (≈32.5 ±5%) | ↓ (≈40.0 ±5%) | ↓ (≈58.0 ±5%) | Kurera et al. 2024 [25] |
| 10µM | 24hr |  | ↓ (≈76.0 ±5%) |  |  |  |  | Son et al. 2024 [26] |
| 10µM | 24hr |  | ↓ (≈41.8 ±>10%) |  |  |  |  | Rahman et al. 2024 [90] |
| 10µM | 24hr |  | ↓ VC |  | ↓ VC |  |  | Kim et al. 2021 [14] |
| 10µM | 24hr |  | ↓ (≈45.1 ±>10%) |  |  |  |  | Zhiyin et al. 2021 [92] |
| 10µM | 24hr |  | ↓ (≈69.6 ±5%) |  |  |  |  | Kim et al 2023 [13] |
| 10µM | 48hr | ↑ (≈270.0 ±>10%) SEM | ↑ (≈152.3 ±>10%) SEM |  |  |  |  | Yang et al. 2023 [93] |
| 10µM | 48hr |  | ↓ (≈44.4-45.0 ±10%) |  | ↓ (≈50.0 ±10%) |  |  | Chen et al. 2021 [31] |
| 10µM | 48hr |  | ↓ (≈59.0 ±10%) |  |  |  |  | Kim et al. 2020 [94] |
| 10µM | 48hr | Varied (MT) | ↓ (≈30.0 ±5%) |  |  |  |  | Pansters et al. 2013 [95] |
| 10µM | 48hr |  |  |  |  |  | **↓** (≈45.4 ±5%) | Gurjar et al. 2020 [96] |
| 10µM | 48hr |  | ↓ VC |  | ↓ VC |  |  | Hyun et al. 2023 [98] |
| 10µM | 48hr |  | ↓ (≈39.0 ±5%) |  | ↓ (≈27.9 ±5%) |  | ↓ (≈66.6 ±>10%) | Ma et al. 2014 [23] |
| 10µM | 96hr |  | ↓ (≈43.3 ±5%) |  |  |  |  | Ma et al. 2014 [23] |
| 10µM | 128hr |  | ↓ (≈50.0 ±5%) |  |  |  |  | Ma et al. 2014 [23] |
| 20µM | 72hr |  | ↓ VC |  |  |  |  | Kweon et al. 2019 [s52] |
| 25µM | 48hr |  | ↓ (≈10.3-55.1 ±>10%) |  |  |  |  | Verhees et al. 2011 [s33] |
| 25µM | 96hr | ↔ Varied (MT) | **↓** VC |  |  |  |  | Chen et al. 2024 [s45] |
| 50µM | 24hr |  | ↓ (≈7.6 ±5%) SEM |  | ↓ (≈21.0 ±5%) SEM |  |  | Sinam et al 2022 [112] |
| 50µM | 24hr |  | ↓ (≈45.7 ±5%) |  | ↓ (≈32.2 ±5%) |  |  | Cid-Diaz et al. 2017 [61] |
| 50µM | 24hr | ↔ Varied (MT) | **↓** VC |  |  |  |  | Li et al. 2023 [101] |
| 50µM | 24hr |  | ↓ VC |  |  |  |  | Yoo et al. 2024 [102] |
| 50µM | 24hr |  | ↓ (≈65.1 ±5%) |  |  |  |  | Geng et al. 2020 [s53] |
| 50µM | 24hr |  | ↓ (≈32.3 ±5%) | ↓ (≈45.1 ±5%) | ↓ (≈39.1 ±5%) |  |  | Li et al. 2021 [103] |
| 50µM | 30hr |  | ↓ (≈31.5 ±5%) |  |  |  | ↓ (≈36.5 ±5%) | Cai et al. 2021 [s54] |
| 50µM | 48hr |  | ↓ (≈61.5 ±>10%) |  |  |  |  | Liang et al. 2024 [108] |
| 100µM | 24hr |  | **↓** VC |  |  |  |  | You et al. 2023 [111] |
| 100µM | 24hr |  | ↓ (≈<5 ±5%) SEM |  | ↓ (≈13.1 ±5%) SEM |  |  | Sinam et al 2022 [112] |
| 100µM | 24hr |  |  | **↓** (≈77.2 ±>10%) |  |  |  | Lee et al. 2018 [38] |
| 100µM | 24hr |  | ↓ (≈45.7 ±5%) |  | ↓ (≈20.3 ±5%) |  |  | Cid-Diaz et al. 2017 [61] |
| 100µM | 24hr |  |  |  |  |  | ↓ VC | Son et al. 2015 [58] |
| 100µM | 24hr | **↓** (≈52.9 ±>10%) | **↓** (≈67.6 ±5%) |  |  |  |  | Han et al. 2021 [s22] |
| 100µM | 24hr |  |  |  |  | ↓ (≈77.7 ±5%) |  | Lee et al. 2023 [118] |
| 100µM | 24hr |  | ↓ (≈68.8 ±5%) |  | ↓ (≈65.2 ±5%) |  |  | Lee et al. 2024 [s23] |
| 100µM | 24hr |  |  | ↑ (≈125.0 ±5%) SEM |  | ↑ (≈266.6 ±>10%) SEM |  | Nguyen et al. 2024 [40] |
| 100µM | 24hr |  |  |  |  | ↑ (≈161.5 ±>10%) SEM | ↔ (≈68.4 ±>10%) SEM | Ko et al. 2024 [44] |
| 100µM | 24hr |  |  |  |  | ↓ (≈76.1±5%) |  | Kang et al. 2024 [s4] |
| 100µM | 24hr |  | ↓ (≈45.2 ±>10%) |  |  |  |  | Go et al. 2024 [s6] |
| 100µM | 24hr |  | ↓ (≈75.8 ±>10%) |  |  |  |  | Wang et al. 2022 [s3] |
| 100µM | 24hr | Varied (MT) | ↓ (≈63.6-80.7 ±>10%) |  |  |  |  | Clarke et al. 2007 [s24] |
| 100µM | 32hr | Varied (MT) | ↓ (≈60.4-66.6 ±>10%) |  |  |  |  | Clarke et al. 2007 [s24] |
| 100µM | 48hr |  | ↓ VC |  | ↓ VC |  | ↓ VC | Cheon et al. 2024 [s8] |
| 100µM | 48hr |  | ↓ (≈66.6 ±5%) SEM |  |  |  |  | Han et al. 2017 [69] |
| 100µM | 48hr |  | ↓ (≈19.5 ±5%) |  | ↓ (≈13.9 ±5%) |  |  | Ma et al. 2014 [23] |
| 100µM | 48hr | ↓ (≈35.4 ±>10%) | ↔ (≈107.6 ±>10%) |  |  |  |  | Kang et al. 2023 [s25] |
| 100µM | 48hr |  | ↓ VC |  |  |  |  | Huang et al. 2023 [s12] |
| 100µM | 48hr | ↓ (≈57.4-63.0 ±5%) |  | ↓ (≈68.0 ±5%) |  | ↓ (≈68.0 ±5%) |  | Jang et al. 2024 [45] |
| 100µM | 72hr |  | ↓ (≈34.0 ±5%) |  |  |  | ↓ (≈29.7 ±5%) | Lee et al. 2024 [46] |
| 200µM | 24hr | **↓** (≈60.7 ±5%) |  | **↓** (≈65.0 ±5%) |  |  |  | Kwak et al. 2022 [s20] |
| 200µM | 24hr |  | ↓ (≈63.2 ±5%) |  |  |  |  | Kim et al. 2024 [32] |

Note: Column are reported as raw values (if available) or as estimates (indicated by ≈) of treatment group expressed as a percent of control ± the variability for the treated group. Variability is listed as SD unless noted with another reporting value (such as SEM). ? indicates the type of variability presented was unclear. “VC” indicates visual confirmation was used to describe the effect of dexamethasone. “Varied MT” indicates that multiple targets were assessed (transcript or isotope variants).

**Abbreviations**: Myh, myosin heavy chain (mRNA); MHC, myosin heavy chain (protein); Myod, myoblast determination protein (mRNA); MYOD, myoblast determination protein (protein); Myog, myogenin (mRNA); MYOG, myogenin (protein).

***Table s3 Effect of dexamethasone on atrophic signaling.***

| Concentration | Duration | *Mstn* | Myostatin | *Murf1* (*Trim63*) | MURF1 | *Mafbx* | Atrogin-1 | Reference |
| --- | --- | --- | --- | --- | --- | --- | --- | --- |
| 0.001µM | 96hr | ↑ VC | ↑ VC |  |  |  |  | Ma et al. 2001 [s55] |
| 0.01µM | 12hr |  |  | ↑ (≈147.0 ±5%) SEM |  | ↑ (≈247.3 ±5%) SEM | ↑ (≈146.3 ±5%) SEM | Jia et al. 2022 [52] |
| 0.01µM | 96hr | ↑ VC | ↑ VC |  |  |  |  | Ma et al. 2001 [s55] |
| 0.01µM | 96hr |  | ↔ (≈181.2 ±>10%) |  |  |  |  | Salehian et al. 2006 [s56] |
| 0.01µM | 192hr |  |  | ↑ (≈171.4 ±5%) SEM |  | ↑ (≈400.0 ±5%) SEM |  | Kim et al. 2016 [54] |
| 0.025µM | 48hr |  |  | ↑ (≈211.7 ±>10%) |  |  |  | Lee et al. 2022 [17] |
| 0.05µM | 24hr |  |  |  | ↔ (≈106.6 ±>10%) |  | ↑ (≈156.2 ±>10%) | Cid-Diaz et al. 2017 [61] |
| 0.05µM | 24hr |  |  | ↔ (≈154.8 ±>10%) |  | ↑ (≈191.6 ±>10%) |  | Menconi et al 2008 [59] |
| 0.05µM | 48hr |  |  | ↑ (≈129.2 ±>10%) | ↑ (≈266.7 ±>10%) | ↑ (≈150.0 ±>10%) | ↑ (≈150.0 ±>10%) | Chang et al 2022 [s57] |
| 0.05µM | 48hr |  |  | ↑ (≈166.6 ±>10%) | ↑ (≈557.1 ±>10%) | ↑ (≈392.8 ±>10%) | ↑ (≈275.0 ±>10%) | Lee et al. 2022 [17] |
| 0.05µM | 48hr |  |  |  | ↑ (≈162.8 ±>10%) |  | ↑ (≈127.6 ±>10%) | Li et al. 2023 [55] |
| 0.1µM | 12hr |  |  | ↑ (≈145.8 ±>10%) | ↑ (≈127.6 ±>10%) | ↑ (≈237.5 ±>10%) | ↑ (≈141.4 ±>10%) | Furukawa et al. 2022 [s58] |
| 0.1µM | 6hr | ↑ (≈355.6 ±>10%) | ↑ (≈196.0 ±>10%) |  |  |  |  | Xie et al. 2018 [s59] |
| 0.1µM | 24hr |  |  |  | ↑ VC |  | ↑ VC | Son et al. 2015 [58] |
| 0.1µM | 24hr |  |  |  |  | ↑ (≈270.8 ±>10%) |  | Sultan et al. 2006 [53] |
| 0.1µM | 24hr |  |  |  |  | ↑ (≈740.0 ±>10%) |  | Zhou et al. 2008 [s60] |
| 0.1µM | 24hr |  |  | ↑ (≈183.9 ±>10%) |  | ↑ (≈315.0 ±>10%) |  | Zhou et al. 2009 [4] |
| 0.1µM | 24hr |  |  | ↑ (≈172.7 ±5%) |  | ↑ (≈256.5 ±5%) |  | Hinds et al. 2016 [s61] |
| 0.1µM | 48hr |  |  | ↑ (≈229.4 ±>10%) |  |  |  | Lee et al. 2022 [17] |
| 0.1µM | 48hr |  |  | ↑ (≈377.8 ±>10%) | ↑ (≈422.2 ±>10%) | ↑ (≈350.0 ±>10%) | ↑ (≈400.0 ±>10%) | Sun et al. 2014 [56] |
| 0.1µM | 96hr | ↑ VC | ↑ VC |  |  |  |  | Ma et al. 2001 [s55] |
| 0.1µM | 96hr |  | ↑ (≈425.0 ±>10%) |  |  |  |  | Salehian et al. 2006 [s56] |
| 0.1µM | 192hr |  |  | ↑ (≈192.8 ±5%) SEM |  | ↑ (≈800.0 ±5%) SEM |  | Kim et al. 2016 [54] |
| 0.5µM | 24hr |  |  |  | ↑ (≈233.3 ±>10%) |  | ↑ (≈350.0 ±>10%) | Cid-Diaz et al. 2017 [61] |
| 0.75µM | 48hr |  |  | ↑ (≈241.1 ±>10%) |  |  |  | Lee et al. 2022 [17] |
| 1µM | 1hr |  |  | ↔ (≈88.9 ±5%) |  | ↔ (≈111.1 ±5%) |  | Yoshioka et al. 2023 [66] |
| 1µM | 4hr |  |  | ↑ (≈194.7 ±5%) SEM |  | ↑ (≈230.0 ±5%) SEM |  | Otsuka et al. 2019 [s62] |
| 1µM | 6hr |  |  | ↔ (≈111.1 ±5%) |  | ↑ (≈166.7 ±5%) |  | Yoshioka et al. 2023 [66] |
| 1µM | 6hr | ↑ (≈213.3 ±5%) SEM | ↑ (≈145.8 ±5%) SEM | ↑ (≈220.0±5%) SEM | ↑ (≈130.4 ±5%) SEM | ↑ (≈246.6 ±5%) SEM | ↑ (≈120.8 ±5%) SEM | Hong et al. 2019 [s48] |
| 1µM | 6hr |  |  |  | ↔ VC |  | ↑ VC | Son et al. 2015 [58] |
| 1µM | 6hr |  |  |  |  | ↑ (≈220.0 ±>10%) |  | Hudson et al. 2014 [s63] |
| 1µM | 12hr |  |  |  | ↑ VC |  | ↑ VC | Son et al. 2015 [58] |
| 1µM | 12hr | ↑ (≈733.3 ±>10%) | ↑ (≈200.0 ±>10%) | ↑ (≈511.1 ±>10%) | ↑ (≈143.3 ±>10%) | ↑ (≈455.6 ±>10%) | ↑ (≈161.9 ±>10%) | Yeon et al. 2020 [s49] |
| 1µM | 24hr |  |  | ↑ (≈248.0 ±>10%) |  | ↑ (≈200.0 ±>10%) |  | Kondo et al. 2022 [s35] |
| 1µM | 24hr |  |  |  | ↑ (≈360.0 ±>10%) |  | ↑ (≈187.8 ±>10%) | Hsieh et al. 2019 [68] |
| 1µM | 24hr |  |  | ↑ (≈191.3 ±>10%) SEM |  |  | ↑ VC | Cerquone Perpetuini et al. 2018 [s36] |
| 1µM | 24hr |  |  | ↔ (≈116.1 ±>10%) |  | ↑ (≈233.3 ±>10%) |  | Menconi et al 2008 [59] |
| 1µM | 24hr |  |  |  | ↑ (≈4400.0 ±>10%) |  | ↑ (≈443.7 ±>10%) | Cid-Diaz et al. 2017 [61] |
| 1µM | 24hr |  |  | ↑ (≈542.9 ±>10%) | ↑ (≈148.3 ±>10%) | ↑ (≈271.4 ±>10%) | ↑ (≈151.7 ±>10%) | Son et al. 2015 [58] |
| 1µM | 24hr |  |  |  | ↔ (≈83.3 ±5%) |  | ↑ (≈183.3 ±>10%) | Lee et al. 2017 [s7] |
| 1µM | 24hr |  |  | ↑ (≈253.3 ±>10%) |  | ↑ (≈346.7 ±>10%) |  | Liu et al. 2016 [12] |
| 1µM | 24hr |  |  | ↑ (≈428.6 ±>10%) | ↑ (≈182.4 ±5%) |  |  | Murata et al. 2017 [s37] |
| 1µM | 24hr |  |  | ↑ (≈190.0 ±>10%) | ↑ (≈170.8 ±5%) |  |  | Sun et al. 2017 [s38] |
| 1µM | 24hr |  |  | ↑ (≈151.5 ±>10%) |  | ↑ (≈470.0 ±>10%) |  | Yoshioka et al. 2019 [s39] |
| 1µM | 24hr |  |  | ↑ (≈179.2 ±>10%) |  | ↑ (≈233.3 ±>10%) |  | Katsuki et al. 2019 [62] |
| 1µM | 24hr |  |  |  | ↑ (≈488.9 ±>10%) |  |  | Murata et al. 2020 [s40] |
| 1µM | 24hr |  |  |  |  | ↑ (≈366.7 ±>10%) |  | Le Bacquer et al. 2020 [63] |
| 1µM | 24hr |  |  |  |  | ↑ (≈330.0 ±>10%) |  | Katsuki et al. 2021 [s41] |
| 1µM | 24hr |  |  |  | ↔ (≈84.2 ±5%) SEM |  | ↑ (≈123.8 ±5%) SEM | Sawano et al. 2021 [64] |
| 1µM | 24hr |  |  |  | ↑ (≈306.3 ±>10%) |  | ↑ (≈346.2 ±>10%) | Yoshioka et al. 2023 [s42] |
| 1µM | 24hr |  |  | ↑ (≈166.7 ±5%) | ↑ (≈216.0 ±>10%) | ↑ (≈377.8 ±5%) | ↑ (≈234.8 ±>10%) | Yoshioka et al. 2023 [66] |
| 1µM | 48hr | ↑ (≈262.5 ±5%) SEM |  |  |  | ↑ (≈168.7 ±5%) SEM |  | Han et al. 2017 [69] |
| 1µM | 48hr |  |  | ↑ (≈381.8 ±5%) SEM |  | ↑ (≈354.5 ±5%) SEM |  | Singh et al. 2017 [70] |
| 1µM | 48hr |  |  | ↑ (≈160 ±>10%) |  | ↑ (≈190 ±>10%) |  | Archer-Lahlou et al. 2018 [76] |
| 1µM | 48hr |  |  |  | ↑ VC |  | ↑ VC | Son et al. 2015 [58] |
| 1µM | 48hr |  |  | ↑ (≈170.0 ±>10%) |  | ↔ (≈196.7 ±>10%) |  | Polge et al. 2011 [s64] |
| 1µM | 48hr | ↑ (≈321.4 ±>10%) | ↑ (≈213.3 ±>10%) | ↑ (≈381.8 ±5%) | ↑ (≈235.3 ±5%) | ↑ (≈342.9 ±5%) | ↑ (≈345.5 ±5%) | Adhikary et al. 2019 [s50] |
| 1µM | 48hr |  | ↑ (150% ±NR) |  | ↑ (150% ±NR) |  | ↑ (170% ±NR) | Han et al. 2020 [71] |
| 1µM | 48hr |  |  |  | ↑ (≈260.0 ±>10%) |  |  | Di Cesare Mannelli et al. 2020 [74] |
| 1µM | 48hr |  |  | ↑ (≈272.7 ±>10%) |  |  |  | Salvadori et al. 2024 [s51] |
| 1µM | 48hr |  |  | ↑ (≈262.5 ±5%) | ↑ (≈191.7 ±>10%) | ↑ (≈620.0 ±5%) | ↑ (≈310.0 ±>10%) | Hah et al. 2023 [72] |
| 1µM | 48hr |  |  | ↑ (≈228.0 ±5%) | ↑ (≈162.5 ±>10%) | ↑ (≈712.5 ±5%) | ↑ (≈366.7 ±>10%) | Hah et al. 2022 [73] |
| 1µM | 48hr |  |  |  | ↑ (≈134.7 ±>10%) |  | ↑ (≈228.0 ±>10%) | Hah et al. 2023 [75] |
| 1µM | 72hr |  |  |  | ↑ VC |  | ↑ VC | Son et al. 2015 [58] |
| 1µM | 192hr |  |  | ↑ (≈307.4 ±5%) SEM |  | ↑ (≈900.0 ±5%) SEM |  | Kim et al. 2016 [54] |
| 2µM | 36hr | ↑ (≈212.0 ±>10%) |  | ↑ (≈363.6 ±>10%) |  | ↑ (≈562.5 ±5%) |  | Yu et al. 2017 [s65] |
| 2.5µM | 24hr |  |  | ↑ (≈628.6 ±5%) |  | ↑ (≈557.8 ±5%) |  | Norikura et al. 2023 [s66] |
| 2.5µM | 48hr |  |  | ↑ (≈242.9 ±5%) |  | ↑ (≈310.0 ±5%) |  | Norikura et al. 2023 [s66] |
| 3µM | 24hr | ↑ (≈266.7 ±>10%) |  |  |  |  |  | Sakai et al. 2018 [s26] |
| 5µM | 24hr |  |  |  | ↑ (≈129.1 ±5%) SEM |  | ↑ (≈174.2 ±5%) SEM | An et al. 2024 [20] |
| 5µM | 24hr |  |  | ↑ (≈181.8 ±>10%) | ↔ (≈129.1 ±>10%) | ↑ (≈218.1 ±>10%) | ↔ (≈132.2 ±>10%) | Choi et al. 2024 [77] |
| 5µM | 24hr |  |  | ↑ (≈170.4 ±>10%) | ↑ VC | ↑ (≈188.9 ±>10%) | ↑ VC | Lee et al. 2022 [21] |
| 5µM | 24hr |  |  | ↑ (≈164.5 ±>10%) |  | ↑ (≈206.5 ±>10%) |  | Kim et al. 2024 [78] |
| 5µM | 24hr |  |  |  | ↑ (≈110.9 ±5%) |  | ↑ (≈145.5 ±5%) | Kim et al. 2022 [22] |
| 5µM | 24hr | ↑ (≈182.1 ±>10%) |  | ↑ (≈200.0 ±>10%) |  | ↑ (≈239.3 ±>10%) |  | Kim et al. 2022 [79] |
| 5µM | 24hr | ↑ (≈166.7 ±>10%) | ↑ VC | ↑ (≈300.0 ±>10%) | ↑ VC | ↑ (≈319.0 ±>10%) | ↑ VC | Lee et al. 2021 [80] |
| 5µM | 24hr | ↑ (≈291.7 ±>10%) |  | ↑ (≈308.3 ±>10%) |  | ↑ (≈208.3 ±>10%) |  | Son et al. 2017 [81] |
| 5µM | 24hr |  |  |  | ↑ (≈278.6 ±5%) |  | ↑ (≈780.0 ±>10%) | Eo et al. 2020 [82] |
| 5µM | 48hr | ↑ (≈108.8 ±>10%) |  | ↑ (≈241.7 ±>10%) |  | ↑ (≈491.7 ±5%) |  | Gan et al. 2022 [83] |
| 10µM | 1hr |  |  | ↔ (121.1% ±NR) |  | ↔ (95.7% ±NR) |  | Tsuchida et al. 2017 [51] |
| 10µM | 1hr |  |  | ↔ (NM) |  | ↓ (NM) |  | Krawiec et al. 2007 [s31] |
| 10µM | 2hr |  |  | ↔ (NM) |  | ↓ (NM) |  | Krawiec et al. 2007 [s31] |
| 10µM | 3hr |  |  | ↑ (160.9% ±NR) |  | ↑ (173.9% ±NR) |  | Tsuchida et al. 2017 [51] |
| 10µM | 4hr |  |  | ↔ (NM) |  | ↑ (≈157.1 ±5%) |  | Krawiec et al. 2007 [s31] |
| 10µM | 6hr |  |  | ↑ (204.3% ±NR) |  | ↑ (169.6% ±NR) |  | Tsuchida et al. 2017 [51] |
| 10µM | 8hr |  |  | ↔ (NM) |  | ↑ (≈169.2 ±5%) |  | Krawiec et al. 2007 [s31] |
| 10µM | 12hr |  |  | ↔ (134.8% ±NR) |  | ↑ (182.6% ±NR) |  | Tsuchida et al. 2017 [51] |
| 10µM | 16hr |  |  | ↔ (NM) |  | ↑ (≈171.4 ±5%) |  | Krawiec et al. 2007 [s31] |
| 10µM | 24hr |  |  | ↑ (≈222.2 ±>10%) | ↑ (≈238.8 ±>10%) | ↑ (≈307.1 ±>10%) | ↑ (≈277.7 ±>10%) | Ma et al. 2024 [30] |
| 10µM | 24hr |  |  |  | ↑ (≈666.6 ±>10%) SEM |  | ↑ (≈450.0 ±>10%) SEM | Sinam et al 2022 [112] |
| 10µM | 24hr | ↑ (≈400.0 ±>10%) |  | ↑ (≈366.6 ±>10%) |  | ↑ (≈333.3 ±>10%) |  | Lee et al. 2022 [84] |
| 10µM | 24hr |  |  | ↑ VC |  |  |  | Waddell et al. 2008 [s67] |
| 10µM | 24hr |  |  |  | ↔ (≈100.0 ±>10%) |  | ↑ (≈375.0 ±>10%) | Lee et al. 2017 [s7] |
| 10µM | 24hr |  |  |  | ↑ VC |  | ↑ VC | Son et al. 2015 [58] |
| 10µM | 24hr |  |  | ↔ (NM) |  | ↑ (≈184.6 ±5%) |  | Krawiec et al. 2007 [s31] |
| 10µM | 24hr |  |  | ↑ (≈132.0 ±>10%) |  | ↑ (≈200.0 ±>10%) |  | Yamamoto et al. 2008 [s32] |
| 10µM | 24hr |  |  | ↑ (≈183.3 ±5%) |  | ↑ (≈312.5 ±5%) |  | Verhees et al. 2011 [s33] |
| 10µM | 24hr |  |  | ↔ (121.6% ±NR) |  | ↑ (173.9% ±NR) |  | Tsuchida et al. 2017 [51] |
| 10µM | 24hr |  |  | ↑ (≈196.8 ±>10%) |  |  |  | Bowen et al. 2017 [86] |
| 10µM | 24hr | ↑ (≈300.0 ±5%) |  | ↑ (≈600.0 ±5%) | ↑ (≈187.5 ±5%) | ↑ (≈1200.0 ±5%) | ↑ (≈187.5 ±5%) | Sakai et al. 2018 [s26] |
| 10µM | 24hr |  |  | ↑ (≈270.0 ±>10%) |  | ↑ (≈470.0 ±>10%) |  | Ulla et al. 2021 [87] |
| 10µM | 24hr |  |  | ↑ (≈233.3 ±5%) | ↑ (≈262.5 ±>10%) | ↑ (≈260.0 ±>10%) | ↑ (≈193.8 ±>10%) | Kimira et al. 2023 [89] |
| 10µM | 24hr |  |  |  | ↑ (162.9% ±5%) SEM |  | ↑ (124.3% ±5%) SEM | Amarasiri et al. 2024 [24] |
| 10µM | 24hr |  |  | ↑ (≈814.3 ±>10%) |  | ↑ (≈173.0 ±>10%) |  | Kurera et al. 2024 [25] |
| 10µM | 24hr | ↑ (≈533.3 ±>10%) |  | ↑ (≈316.7 ±>10%) | ↑ (≈125.0 ±>10%) | ↑ (≈1116.7 ±>10%) | ↑ (≈120.0 ±>10%) | Son et al. 2024 [26] |
| 10µM | 24hr |  |  | ↑ (≈225.0 ±>10%) |  | ↑ (≈363.6 ±>10%) |  | Rahman et al. 2024 [90] |
| 10µM | 24hr |  |  |  | ↑ VC |  | ↑ VC | Kim et al. 2021 [14] |
| 10µM | 24hr |  |  | ↑ (≈200.0 ±>10%) |  | ↑ (≈423.1 ±>10%) | ↑ (≈511.1 ±>10%) | Kim et al. 2022 [91] |
| 10µM | 24hr |  |  |  | ↑ (≈171.4 ±>10%) |  | ↑ (≈217.1 ±>10%) | Zhiyin et al. 2021 [92] |
| 10µM | 24hr |  |  | ↑ (≈380.0 ±5%) | ↑ (≈235.7 ±>10%) | ↑ (≈900.0 ±>10%) | ↑ (≈414.3 ±>10%) | Wang et al. 2021 [18] |
| 10µM | 24hr |  |  |  | ↑ (≈157.4 ±>10%) |  | ↑ (≈118.0 ±>10%) | Kim et al. 2023 [13] |
| 10µM | 24hr |  |  |  | ↑ (≈158.8 ±>10%) |  | ↑ (≈241.2 ±>10%) | Shen et al. 2019 [28] |
| 10µM | 48hr | ↑ (≈270.0 ±>10%) SEM | ↑ (≈152.3 ±>10%) SEM |  | ↑ (≈168.4 ±>10%) SEM |  | ↑ (≈142.3 ±>10%) SEM | Yang et al. 2023 [93] |
| 10µM | 48hr |  |  |  | ↑ (≈179.1-181.4 ±>10%) |  | ↑ (≈220.8-225.0 ±>10%) | Chen et al. 2021 [31] |
| 10µM | 48hr |  |  | ↑ (median≈252.1 ±?%) |  | ↑ (median≈364.7 ±?%) |  | Tomiya et al. 2019 [s34] |
| 10µM | 48hr | ↑ (≈312.5 ±5%) SEM |  |  |  | ↑ (≈306.2 ±5%) SEM |  | Han et al. 2017 [69] |
| 10µM | 48hr | ↑ (≈178.5 ±>10%) SEM |  |  |  |  |  | Allen et al. 2011 [s27] |
| 10µM | 48hr |  |  | ↑ VC |  |  |  | Waddell et al. 2008 [s67] |
| 10µM | 48hr |  |  |  |  |  | ↑ (≈900.0 ±>10%) | Kim et al. 2020 [94] |
| 10µM | 48hr | ↑ (≈164.7 ±>10%) |  | ↑ (≈205.9 ±>10%) |  | ↑ (≈441.2 ±>10%) |  | Massaccesi et al. 2016 [s28] |
| 10µM | 48hr | ↑ (≈920.0 ±>10%) |  | ↑ (≈560.0 ±>10%) | ↑ (≈320.0 ±>10%) | ↑ (≈520.0 ±>10%) | ↑ (≈310.0 ±>10%) | Gurjar et al. 2020 [96] |
| 10µM | 72hr |  |  | ↑ VC |  |  |  | Waddell et al. 2008 [s67] |
| 10µM | 72hr |  |  |  | ↑ VC |  | ↑ VC | Yoon et al. 2022 [s15] |
| 20µM | 72hr |  |  |  | ↔ VC |  | ↑ VC | Kweon et al. 2019 [s52] |
| 25µM | 24hr |  |  | ↑ (≈188.9 ±5%) |  | ↑ (≈362.5 ±5%) |  | Verhees et al. 2011 [s33] |
| 25µM | 96hr |  |  | ↑ (≈223.8 ±>10%) |  | ↑ (≈458.3 ±>10%) |  | Chen et al. 2024 [s45] |
| 30µM | 24hr |  |  | ↑ (≈222.2 ±>10%) | ↑ (≈261.1 ±>10%) | ↑ (≈307.1 ±>10%) | ↑ (≈266.6 ±>10%) | Ma et al. 2024 [30] |
| 40µM | 24hr |  |  | **↓** (≈186.6-196.4 ±10%) |  | **↓** (≈329.4-472.7 ±10%) |  | Aguilar-Agon et al. 2020 [99] |
| 50µM | 3hr |  |  | ↑ (≈190.9 ±>10%) |  | ↑ (≈228.6 ±>10%) |  | Nguyen et al. 2020 [100] |
| 50µM | 6hr |  |  | ↑ (≈190.9 ±>10%) |  | ↑ (≈264.3 ±>10%) |  | Nguyen et al. 2020 [100] |
| 50µM | 24hr |  |  |  | ↑ (≈700.0 ±>10%) SEM |  | ↑ (≈350.0 ±>10%) SEM | Sinam et al 2022 [112] |
| 50µM | 24hr |  |  | ↑ (≈244.4 ±>10%) | ↑ (≈277.7 ±>10%) | ↑ (≈321.4 ±>10%) | ↑ (≈244.4 ±>10%) | Ma et al. 2024 [30] |
| 50µM | 24hr |  |  |  | ↑ (≈413.3 ±>10%) |  | ↑ (≈443.7 ±>10%) | Cid-Diaz et al. 2017 [61] |
| 50µM | 24hr |  |  |  | ↑ (≈325.0 ±>10%) |  | ↑ (≈441.7 ±>10%) | Lee et al. 2017 [s7] |
| 50µM | 24hr |  |  | ↔ (≈168.2 ±>10%) |  | ↑ (≈285.7 ±>10%) |  | Nguyen et al. 2020 [100] |
| 50µM | 24hr |  |  | ↑ (≈144.4 ±>10%) | ↑ VC | ↑ (≈225.0 ±>10%) | ↑ VC | Yoo et al. 2024 [102] |
| 50µM | 24hr |  |  | ↑ (≈628.6 ±>10%) SEM | ↑ (≈215.8 ±5%) SEM | ↑ (≈164.3 ±>10%) SEM | ↑ (≈205.6 ±>10%) SEM | Sun et a. 2024 [36] |
| 50µM | 24hr |  |  | ↑ (≈225.0 ±>10%) |  | ↑ (≈366.7 ±>10%) |  | Geng et al. 2020 [s53] |
| 50µM | 24hr |  |  | ↑ (≈223.8 ±>10%) | ↑ (≈350.0 ±>10%) | ↑ (≈293.3 ±>10%) | ↑ (≈357.1 ±>10%) | Li et al. 2021 [103] |
| 50µM | 24hr |  |  | ↑ (≈441.7 ±>10%) | ↑ VC | ↑ (≈728.6 ±>10%) | ↑ VC | Zeng et al. 2024 [s68] |
| 50µM | 24hr |  |  | ↑ (≈233.3 ±5%) SEM |  | ↑ (≈340.0 ±5%) SEM |  | Li et al. 2017 [15] |
| 50µM | 30hr |  |  | ↑ (≈173.6-304.5 ±>10%) |  | ↑ (≈150.0-168.4 ±>10%) |  | Cai et al. 2021 [s54] |
| 50µM | 36hr |  |  | ↑ (≈140.5 ±>10%) SEM |  | ↑ (≈151.3 ±>10%) SEM |  | Li et al. 2018 [104] |
| 50µM | 48hr |  |  | ↑ (≈162.5 ±5%) |  | ↑ (≈293.7 ±5%) |  | Liu et al. 2023 [105] |
| 50µM | 48hr |  |  |  | ↑ (≈290.0 ±10%) | ↑ (≈300.0 ±10%) |  | Jeon et al. 2021 [107] |
| 50µM | 48hr |  |  | ↑ (≈357.1 ±>10%) |  |  |  | Shen et al. 2013 [s43] |
| 50µM | 48hr |  |  |  | ↑ (≈246.2 ±>10%) SEM |  | ↑ (≈203.8 ±>10%) SEM | Liang et al. 2024 [108] |
| 100µM | 3hr |  |  | ↑ (≈400.0 ±5%) SEM | ↔ (≈106.3 ±5%) SEM | ↑ (≈118.6 ±5%) SEM | ↔ (≈100.0 ±5%) SEM | Wang et al. 2017 [s44] |
| 100µM | 8hr |  |  | ↑ (≈309.0 ±5%) SEM | ↑ (≈114.8 ±5%) SEM | ↓ (≈67.4 ±5%) SEM | ↑ (≈121.8 ±5%) SEM | Wang et al. 2017 [s44] |
| 100µM | 12hr |  |  | ↑ (≈236.3 ±5%) SEM |  | ↓ (≈65.1 ±5%) SEM |  | Wang et al. 2017 [s44] |
| 100µM | 24hr |  |  | ↑ (≈288.8 ±>10%) | ↑ (≈272.2 ±>10%) | ↑ (≈342.8 ±>10%) | ↑ (≈244.4 ±>10%) | Ma et al. 2024 [30] |
| 100µM | 24hr |  |  | ↑ (≈263.6 ±5%) SEM |  | ↔ (≈90.6 ±5%) SEM |  | Wang et al. 2017 [s44] |
| 100µM | 24hr |  |  |  |  | ↑ (≈185.7-234.7 ±>10%) SEM |  | Sugiyama et al. 2012 [s69] |
| 100µM | 24hr |  |  |  | ↑ (≈154.1 ±>10%) SEM |  | ↑ (≈150.0 ±>10%) | Park et al. 2023 [s70] |
| 100µM | 24hr |  |  |  | ↑ (≈833.3 ±>10%) SEM |  | ↑ (≈1250.0 ±>10%) SEM | Sinam et al 2022 [112] |
| 100µM | 24hr |  |  | ↑ (≈338.4 ±>10%) | ↑ (≈210.0 ±>10%) | ↑ (≈219.0 ±>10%) | ↑ (≈195.0 ±>10%) | Lee et al. 2018 [38] |
| 100µM | 24hr |  |  |  | ↑ (≈137.5 ±>10%) |  | ↑ (≈381.8 ±>10%) | Hemdan et al. 2009 [s71] |
| 100µM | 24hr | ↑ (≈160.7 ±>10%) | ↑ VC | ↑ (≈160.7 ±>10%) |  | ↑ (≈164.2 ±>10%) | ↑ VC | Proserpio et al. 2013 [s29] |
| 100µM | 24hr |  |  |  | ↑ (≈413.3 ±>10%) |  | ↑ (≈456.2 ±>10%) | Cid-Diaz et al. 2017 [61] |
| 100µM | 24hr |  |  |  | ↑ VC |  | ↑ VC | Son et al. 2015 [58] |
| 100µM | 24hr |  |  |  | ↑ (≈458.3 ±>10%) |  | ↑ (≈491.7 ±>10%) | Lee et al. 2017 [s7] |
| 100µM | 24hr |  |  | ↑ (≈240.0 ±>10%) SEM |  | ↑ (≈300.0 ±>10%) SEM |  | Tobimatsu et al 2009 [s72] |
| 100µM | 24hr |  |  | ↑ (≈205.6 ±5%) |  | ↑ (≈487.5 ±5%) |  | Verhees et al. 2011 [s33] |
| 100µM | 24hr |  | ↑ VC |  | ↑ (≈136.8 ±>10%) SEM |  | ↑ (≈142.1 ±>10%) SEM | Kukreti et al. 2013 [s30] |
| 100µM | 24hr |  |  | ↑ (≈900.0 ±5%) |  |  | ↑ (≈333.3 ±5%) | Reinoso-Sánchez et al. 2020 [116] |
| 100µM | 24hr |  |  | ↑ (≈242.9 ±>10%) SEM |  | ↑ (≈384.6 ±>10%) SEM |  | Ozaki et al. 2022 [117] |
| 100µM | 24hr |  |  | ↑ (≈430.0 ±>10%) |  | ↑ (≈340.0 ±>10%) |  | Maier et al. 2023 [110] |
| 100µM | 24hr |  |  | ↑ (≈400.0 ±>10%) | ↑ (≈195.4 ±>10%) | ↑ (≈1100.0 ±>10%) |  | Lee et al. 2023 [118] |
| 100µM | 24hr |  |  | ↑ (≈293.3 ±>10%) SEM | ↑ VC | ↑ (≈500.0 ±>10%) SEM | ↑ VC | Kim et al. 2023 [119] |
| 100µM | 24hr |  |  | ↑ (≈241.2 ±5%) |  | ↑ (≈600.0 ±5%) |  | Lee et al. 2024 [s23] |
| 100µM | 24hr |  |  | ↑ (≈132.6 ±5%) |  | ↑ (≈305.0 ±5%) |  | Hur er al. 2024 [29] |
| 100µM | 24hr |  |  |  | ↑ (≈129.7 ±5%) SEM |  | ↑ (≈166.6 ±5%) SEM | Nguyen et al. 2024 [40] |
| 100µM | 24hr |  |  |  |  | ↑ (≈511.1 ±5%) SEM | ↑ (≈192.3 ±5%) SEM | Han et al. 2024 [41] |
| 100µM | 24hr | ↑ (≈612.5 ±>10%) | ↑ (≈150.0 ±>10%) | ↑ (≈445.5 ±>10%) | ↑ (≈160.7 ±>10%) | ↑ (≈628.6 ±>10%) | ↑ (≈600.0 ±>10%) | Jo et al. 2024 [42] |
| 100µM | 24hr |  | ↑ (≈164.7 ±>10%) |  | ↑ (≈254.5 ±>10%) |  | ↑ (≈335.3 ±>10%) | Go et al. 2024 [s6] |
| 100µM | 24hr |  |  |  | ↑ (≈188.9 ±>10%) SEM |  | ↑ (≈275.0 ±>10%) SEM | Edwards et al. 2022 [s1] |
| 100µM | 24hr |  |  | ↑ (≈400.0 ±>10%) | ↑ (≈311.1 ±>10%) | ↑ (≈1225.0 ±>10%) | ↑ (≈133.3 ±>10%) | Kang et al. 2024 [s4] |
| 100µM | 24hr | ↑ (≈160.7 ±>10%) SEM |  | ↑ (≈300.0 ±>10%) SEM | ↑ (≈271.4 ±NR%) SEM | ↑ (≈671.4 ±>10%) SEM | ↑ (≈284.6 ±NR%) SEM | Choi et al. 2022 [43] |
| 100µM | 24hr |  |  | ↑ (≈204.7 ±>10%) SEM | ↑ (≈262.5 ±>10%) SEM | ↑ (≈326.3 ±>10%) SEM | ↑ (≈675.0 ±>10%) SEM | Kim et al. 2018 [s2] |
| 100µM | 24hr |  |  | ↑ (≈500.0 ±>10%) SEM |  | ↑ (≈1040.0 ±>10%) SEM |  | Park et al. 2020 [35] |
| 100µM | 24hr |  |  |  | ↑ (≈121.4 ±>10%) |  | ↑ (≈200.0 ±5%) | Kim et al. 2023 [39] |
| 100µM | 24hr | ↑ (≈194.7 ±5%) SEM |  | ↑ (≈283.3 ±5%) SEM | ↑ VC |  | ↑ VC | Ko et al. 2024 [44] |
| 100µM | 24hr |  | ↑ (≈160.0 ±>10%) |  | ↑ (≈170.0 ±>10%) |  | ↑ (≈175.9 ±>10%) | Wang et al. 2022 [s3] |
| 100µM | 24hr |  |  |  | ↑ VC |  |  | Clarke et al. 2007 [s24] |
| 100µM | 32hr |  |  |  | ↑ VC |  |  | Clarke et al. 2007 [s24] |
| 100µM | 48hr |  |  | ↑ (≈246.6 ±>10%) | ↑ VC | ↑ (≈293.3 ±>10%) | ↑ VC | Cheon et al. 2024 [s8] |
| 100µM | 48hr |  |  | ↑ (≈181.8 ±5%) | ↑ VC | ↑ (≈282.3 ±5%) | ↑ VC | You et al. 2023 [111] |
| 100µM | 48hr |  |  |  |  | ↑ (≈394.1 ±>10%) SEM | ↑ (≈871.4 ±>10%) SEM | Pierucci et al. 2021 [s73] |
| 100µM | 48hr |  |  |  |  |  | ↑ (≈800.0 ±10%) SEM | Pierucci et al. 2017 [s74] |
| 100µM | 48hr | ↔ (≈143.7 ±5%) SEM |  |  |  | ↑ (≈343.7 ±5%) SEM | ↑ (≈236.8 ±5%) SEM | Han et al. 2017 [69] |
| 100µM | 48hr |  |  |  |  | ↑ VC |  | Rossi et al. 2009 [s9] |
| 100µM | 48hr |  |  |  | ↑ (≈142.5 ±>10%) SEM |  | ↑ (≈200.0 ±>10%) SEM | Chang et al. 2020 [s10] |
| 100µM | 48hr |  |  | ↑ (≈200.0 ±>10%) | ↑ VC | ↑ (≈288.2 ±5%) | ↑ VC | Li et al. 2021 [s11] |
| 100µM | 48hr |  |  |  | ↑ (≈165.2 ±>10%) | ↔ (≈150.0 ±>10%) | ↑ (≈137.1 ±>10%) | Kang et al. 2023 [s25] |
| 100µM | 48hr |  |  | ↑ (≈446.7 ±>10%) |  | ↑ (≈971.4 ±>10%) |  | Jang et al. 2024 [45] |
| 100µM | 48hr |  |  | ↑ (≈292.9 ±>10%) |  | ↑ (≈390.0 ±>10%) |  | Jeong et al. 2024 [s13] |
| 100µM | 48hr |  |  |  | ↑ (≈167.9 ±5%) |  | ↑ (≈164.0 ±5%) | Chen et al. 2019 [s14] |
| 100µM | 72hr |  |  | ↑ (≈161.1-175.9 ±>10%) |  | ↑ (≈266.7-276.2 ±>10%) |  | Nakagawara et al. 2023 [s16] |
| 100µM | 96hr |  |  | ↑ (≈182.1 ±>10%) |  | ↑ (≈477.7 ±>10%) |  | Wang et al. 2023 [s17] |
| 150µM | 24hr |  |  |  | ↑ (≈187.1 ±>10%) SEM |  |  | Gwag et al. 2013 [s18] |
| 150µM | 24hr |  |  | ↑ (≈148.7 ±>10%) |  | ↑ (≈184.0 ±5%) |  | Bae et al. 2024 [s19] |
| 200µM | 24hr |  |  |  | ↑ (≈142.0 ±5%) |  | ↑ (≈222.6 ±>10%) | Men et al. 2024 [33] |
| 200µM | 24hr |  |  |  | ↑ (≈268.4 ±>10%) |  | ↑ (≈137.8 ±>10%) | Men et al. 2024 [48] |
| 200µM | 24hr |  |  |  | ↑ (≈225.8 ±>10%) |  | ↑ (≈176.3 ±>10%) | Jiang et al. 2019 [49] |
| 200µM | 24hr |  |  |  | ↑ (≈163.0 ±5%) |  | ↑ (≈161.5 ±>10%) | Wang et al. 2020 [s21] |
| 200µM | 24hr |  |  | ↑ (≈158.6 ±>10%) | ↑ (≈171.0 ±>10%) | ↑ (≈169.0 ±>10%) | ↑ (≈159.4 ±>10%) | Wang et al. 2021 [34] |

Note: Column are reported as raw values (if available) or as estimates (indicated by ≈) of treatment group expressed as a percent of control ± the variability for the treated group. Variability is listed as SD unless noted with another reporting value (such as SEM). ? indicates the type of variability presented was unclear. “VC” indicates visual confirmation was used to describe the effect of dexamethasone. “Varied MT” indicates that multiple targets were assessed (transcript or isotope variants). “NM” indicates that relative assessments were *not measurable*.

**Abbreviations**: *Mstn*, myostatin (mRNA); *Mafbx* or *Fbx32*, muscle atrophy x box (mRNA); *Murf1* or *Trim63*, Muscle RING-finger protein-1 (mRNA); MURF1, Muscle RING-finger protein-1 (protein).

***Table s4 Effect of dexamethasone on Foxo1/3 signaling.***

| Concentration | Duration | *Foxo1* | FOXO1 | p-FOXO1 | *Foxo3* | FOXO3 | p-FOXO3 | Reference |
| --- | --- | --- | --- | --- | --- | --- | --- | --- |
| 0.01µM | 48hr | ↓ (≈85.1 ±5%) SEM |  |  | ↑ (≈328.0 ±5%) SEM |  |  | Kim et al. 2016 [54] |
| 0.05µM | 48hr |  |  |  | ↑ (≈191.3-213.5 ±5%) SEM |  | ↑ (≈209.8 ±5%) SEM | Chang et al 2022 [s57] |
| 0.05µM | 48hr |  |  |  | ↑ (≈261.3 ±5%) | ↑ (≈181.8 ±5%) |  | Lee et al. 2022 [17] |
| 0.1µM | 48hr | ↔ (≈95.9 ±5%) SEM |  |  | ↑ (≈332.0 ±5%) SEM |  |  | Kim et al. 2016 [54] |
| 1µM | 0.5hr |  |  | ↓ (≈88.8 ±5%) SEM |  |  | ↑ (≈170.6 ±10%) SEM | Yoshioka et al. 2019 [s39] |
| 1µM | 4hr |  |  |  |  |  | ↓ (≈68.4 ±10%) SEM | Otsuka et al. 2019 [s62] |
| 1µM | 6hr |  |  |  | ↑ (≈166.6 ±5%) |  |  | Hong et al. 2019 [s48] |
| 1µM | 6hr |  |  |  | ↑ (≈238.8 ±5%) SEM |  |  | Hudson et al. 2014 [s63] |
| 1µM | 12hr |  |  | ↓ (≈58.0 ±5%) |  |  |  | Yeon et al. 2020 [s49] |
| 1µM | 24hr |  | ↓ (≈82.3 ±5%) SEM | ↓ (≈46.6 ±5%) SEM |  | ↑ (≈127.2 ±5%) SEM | ↓ (≈63.4 ±5%) SEM | Cid-Diaz et al. 2017 [61] |
| 1µM | 24hr |  |  | ↑  (≈144.0 ±5%) SEM |  |  | ↑ (≈180.0±5%) SEM | Lu et al. 2013 [60] |
| 1µM | 24hr |  |  |  |  | ↑ (≈167.5 ±5%) SEM |  | Liu et al. 2016 [12] |
| 1µM | 24hr |  | ↔ (≈93.9 ±5%) SEM |  |  | ↔ (≈94.4 ±5%) SEM |  | Murata et al. 2017 [s37] |
| 1µM | 24hr |  | ↓ (≈89.1 ±5%) SEM | ↓ (≈43.2-100.0 ±5%) SEM |  | ↑ (≈125.0 ±5%) SEM | ↓ (≈57.8-100.0 ±5%) SEM | Cid-Diaz et al. 2017 [61] |
| 1µM | 24hr |  |  |  | ↑ (≈109.0 ±10%) |  |  | Katsuki et al. 2019 [62] |
| 1µM | 24hr |  |  |  |  | ↑ (≈130.5 ±5%) SEM |  | Murata et al. 2020 [s40] |
| 1µM | 24hr |  |  | ↓ (≈96.3 ±5%) |  |  | ↑ (≈274.3 ±10%) | Yoshioka et al. 2023 [s42] |
| 1µM | 24hr |  |  |  |  | ↑ (≈156.2 ±10%) | ↓  (≈74.7 ±10%) | Yoshioka et al. 2023 [66] |
| 1µM | 48hr |  | ↓ (≈76.9 ±10%) |  |  | ↑ (≈148.7 ±5%) |  | Hah et al. 2023 [72] |
| 1µM | 48hr |  | ↓ (≈67.7±5%) |  |  | ↑ (≈143.2 ±5%) |  | Hah et al. 2022 [73] |
| 1µM | 48hr | ↑ (≈110.8±5%) SEM |  |  | ↑ (≈344.0 ±5%) SEM |  |  | Kim et al. 2016 [54] |
| 1µM | 48hr |  |  |  | ↑ (≈333.3±5%) |  |  | Salvadori et al. 2024 [s51] |
| 1µM | 48hr |  |  | ↓ (≈3.84 ±5%) |  |  |  | Singh et al. 2017 [70] |
| 1µM | 48hr |  |  |  |  | ↑ (≈242.2 ±5%) |  | Hah et al. 2023 [75] |
| 2µM | 36hr |  |  | ↓ (≈53.4±5%) SEM |  |  |  | Yu et al. 2017 [s65] |
| 3µM | 24hr |  |  |  |  |  | ↓ (≈48.2 ±5%) SEM | Sakai et al. 2018 [s26] |
| 5µM | 24hr |  |  |  |  | ↑ (≈228.9±5%) SEM | ↓ (≈71.0 ±5%) SEM | An et al. 2024 [20] |
| 5µM | 24hr |  |  | ↓ (≈56.8 ±5%) |  |  |  | Choi et al. 2024 [77] |
| 5µM | 24hr |  | ↔ (≈106.8±5%) SEM | ↓ (≈51.1±5%) SEM |  | ↑ (≈125.2±5%) SEM | ↑ (≈185.4 ±5%) SEM | Cid-Diaz et al. 2017 [61] |
| 5µM | 24hr |  |  |  |  | ↑ (≈160.9±5%) |  | Kim et al. 2022 [22] |
| 5µM | 24hr |  |  |  |  | ↓ cyt (≈73.6 ±5%) SEM / ↑ nuc (≈184.2±5%) SEM |  | Kim et al. 2022 [79] |
| 5µM | 24hr |  |  |  |  | ↔ VC | ↓ VC | Lee et al. 2021 [80] |
| 5µM | 24hr |  | ↔ VC | ↓ (≈50.0 ±5%) |  |  |  | Son et al. 2017 [81] |
| 10µM | 1hr | ↑ (≈123.2 ±5%) |  |  | ↑ (≈129.0 ±5%) |  |  | Tsuchida et al. 2017 [51] |
| 10µM | 3hr | ↑ (≈110.1 ±5%) |  |  | ↑ (≈198.3 ±5%) |  |  | Tsuchida et al. 2017 [51] |
| 10µM | 6hr | ↓ (≈86.8 ±5%) |  |  | ↑ (≈174.1 ±5%) |  |  | Tsuchida et al. 2017 [51] |
| 10µM | 12hr | ↔ (≈97.9 ±5%) |  |  | ↑ (≈132.2 ±5%) |  |  | Tsuchida et al. 2017 [51] |
| 10µM | 24hr |  |  |  | ↑ (≈154.1 ±5%) | ↑ (≈144.4 ±5%) |  | Ma et al. 2024 [30] |
| 10µM | 24hr |  |  |  |  |  | ↓ (≈66.2 ±5%) | Perpetuini et al. 2018 [s36] |
| 10µM | 24hr | ↓ (≈83.8 ±5%) | ↑ (≈115.0 ±5%) | ↓ (≈51.4 ±5%) | ↑ (≈125.8 ±5%) | ↑ (≈151.3 ±5%) | ↓ (≈50.6 ±5%) | Tsuchida et al. 2017 [51] |
| 10µM | 24hr |  |  |  |  | ↑ (≈195.0 ±5%) | ↓ (≈24.0 ±5%) | Ulla et al. 2021 [87] |
| 10µM | 24hr |  |  |  |  |  | ↓ (≈22.6 ±5%) | Kimira et al. 2023 [89] |
| 10µM | 24hr |  |  |  | ↑(≈203.0 ±5%) | ↑ (≈642.8 ±5%) |  | Kurera et al. 2024 [25] |
| 10µM | 24hr |  |  |  |  | ↑ (≈386.2 ±5%) | ↓ (≈11.5 ±5%) | Rahman et al. 2024 [90] |
| 10µM | 24hr |  |  |  |  | ↑ VC |  | Kim et al. 2021 [14] |
| 10µM | 24hr |  |  |  |  | ↑ (≈200.0 ±10%) | ↓ (≈31.3 ±10%) | Kim et al. 2022 [91] |
| 10µM | 24hr |  |  |  |  | ↑ (≈145.6 ±5%) SEM | ↓ (≈56.5 ±5%) SEM | Zhiyin et al. 2021 [92] |
| 10µM | 24hr |  |  |  |  | ↑ VC | ↓ (≈40.5 ±5%) SEM | Wang et al. 2021 [18] |
| 10µM | 24hr |  |  |  |  | ↑ (≈214.6 ±10%) |  | Kim et al. 2023 [13] |
| 10µM | 24hr |  |  |  |  |  | ↓ (≈43.9±5%) | Shen et al. 2019 [28] |
| 10µM | 48hr |  |  |  |  |  | ↓ (≈57.5 ±5%) | Chen et al. 2021 [31] |
| 10µM | 48hr |  |  | ↓ (≈57.8 ±5%) |  |  |  | Gurjar et al. 2020 [96] |
| 10µM | 48hr |  |  |  |  | ↑ (≈160.0 ±5%) SEM | ↓ (≈68.5 ±5%) SEM | Yang et al. 2023 [93] |
| 30µM | 24hr |  |  |  | ↑ (≈150.0 ±5%) | ↑ (≈146.6 ±5%) |  | Ma et al. 2024 [30] |
| 50µM | 24hr |  | ↔ (≈106.8±5%) SEM | ↓ (≈55.5±5%) SEM |  | ↑ (≈117.2±5%) SEM | ↑ (≈146.7 ±5%) SEM | Cid-Diaz et al. 2017 [61] |
| 50µM | 24hr |  | ↑ (≈149.2 ±5%) |  |  |  |  | Li et al. 2021 [103] |
| 50µM | 24hr |  |  |  | ↑ (≈204.1 ±5%) | ↑ (≈153.3 ±5%) |  | Ma et al. 2024 [30] |
| 50µM | 24hr |  |  |  |  | ↓ cyt (≈59.3±5%) / ↑ nuc (≈168.5±5%) |  | Wang et al. 2021 [34] |
| 50µM | 48hr | ↑ (≈175.8 ±5%) |  |  | ↑ (≈238.8 ±5%) |  |  | Shen et al. 2013 [s43] |
| 100µM | 8hr | ↑ (≈207.6 ±5%) SEM | ↑ (≈142.5±5%) SEM | ↑ (≈157.5 ±5%) SEM | ↓ (≈62.5 ±5%) SEM |  | ↑ (≈273.1 ±5%) SEM | Wang et al. 2017 [s44] |
| 100µM | 24hr |  | ↔  (≈103.4±5%) SEM | ↓ (≈47.7±5%) SEM |  | ↑ (≈121.8±5%) SEM | ↑ (≈129.0 ±5%) SEM | Cid-Diaz et al. 2017 [61] |
| 100µM | 24hr |  |  |  |  |  | ↓ (≈73.2 ±5%) SEM | Kukreti et al. 2013 [s30] |
| 100µM | 24hr |  | ↓ cyt (≈26.6 ±5%) / ↑ nuc (≈203.3 ±5%) / ↑ total (≈238.7 ±5%) | ↓ (≈55.1 ±5%) |  | ↓ cyt (≈57.5 ±5%) / ↑ nuc (≈214.6 ±5%) / ↑ (≈262.9 ±5%) | ↓ (≈81.3 ±5%) | Lee et al. 2018 [38] |
| 100µM | 24hr |  | ↓ cyt (≈41.8 ±5%) / ↑ nuc (≈234.4 ±5%) / ↑ (≈231.0 ±5%) | ↓ (≈42.2 ±5%) |  | ↓ cyt (≈75.0 ±5%) / ↑ nuc (≈172.5 ±5%) / ↑ (≈523.0 ±5%) | ↓ (≈15.7 ±5%) | Lee et al. 2019 [37] |
| 100µM | 24hr |  |  |  |  |  | ↓ (≈53.5 ±5%) | Lee et al. 2024 [s23] |
| 100µM | 24hr |  | ↑ (≈202.0±5%) | **↓** (≈40.2±5%) |  |  |  | Hur er al. 2024 [29] |
| 100µM | 24hr |  |  |  | ↑ (≈212.5 ±5%) | ↑ (≈157.7 ±5%) |  | Ma et al. 2024 [30] |
| 100µM | 24hr |  |  |  |  |  | ↑ (≈242.8 ±5%) SEM | Nguyen et al. 2024 [40] |
| 100µM | 24hr |  |  |  |  | ↑ (≈219.1 ±5%) |  | Jo et al. 2024 [42] |
| 100µM | 24hr |  |  |  | ↑ (≈304.1 ±5%) SEM |  | ↓ (≈47.2 ±5%) SEM | Choi et al. 2022 [43] |
| 100µM | 24hr |  |  |  | ↑ (≈584.6 ±5%) SEM |  |  | Park et al. 2020 [35] |
| 100µM | 24hr |  |  |  |  |  | ↑ (≈142.8 ±5%) SEM | Ko et al. 2024 [44] |
| 100µM | 24hr |  |  |  |  |  | ↓ (≈54.5 ±5%) | Wang et al. 2022 [s3] |
| 100µM | 48hr |  |  |  |  |  | ↓ (≈26.1 ±10%) SEM | Chang et al. 2020 [s10] |
| 100µM | 48hr | ↑ (≈316.0±5%) SEM | ↑ (≈219.5±5%) SEM |  |  |  |  | Qin et al. 2014 [s75] |
| 100µM | 48hr |  |  |  |  |  | ↓ (≈32.4 ±5%) | Jang et al. 2024 [45] |
| 100µM | 48hr |  |  |  | ↑ (≈309.5±5%) SEM |  | ↓ (≈78.3±5%) SEM | Jeong et al. 2024 [s13] |
| 100µM | 48hr |  |  |  |  |  | ↓ (≈42.1±5%) | Chen et al. 2019 [s14] |
| 150µM | 24hr |  |  |  |  |  | ↓ (≈47.0±5%) SEM | Gwag et al. 2013 [s18] |
| 200µM | 24hr |  |  |  |  |  | ↓ (≈75.0±5%) SEM | Kim et al. 2024 [32] |
| 200µM | 24hr |  |  |  |  | ↑ (≈232.4-258.3±5%) |  | Men et al. 2024 [33] |
| 200µM | 24hr |  |  |  |  | ↑ (≈138.7 ±10%) |  | Men et al. 2024 [48] |

Note: Column are reported as raw values (if available) or as estimates (indicated by ≈) of treatment group expressed as a percent of control ± the variability for the treated group. Variability is listed as SD unless noted with another reporting value (such as SEM). ? indicates the type of variability presented was unclear. “VC” indicates visual confirmation was used to describe the effect of dexamethasone. “Varied MT” indicates that multiple targets were assessed (transcript or isotope variants). “NM” indicates that relative assessments were *not measurable*.

**Abbreviations**: Cyt, cytosolic; *Foxo1*, Forkhead box protein 01 (mRNA); FOXO1, Forkhead box protein 01 (protein); *Foxo3*, Forkhead box protein 03 (mRNA); FOXO3, Forkhead box protein 01 (protein); Nuc, nuclear.

***Table s5 Effect of dexamethasone on anabolic signaling.***

| Concentration | Duration | p-Akt | p-mTOR | p-P70s6k | p-4EBP1 | p-RPS6 | puromycin incorporation | Reference |
| --- | --- | --- | --- | --- | --- | --- | --- | --- |
| 0.01µM | 192hr | ↓ (≈31.1 ±5%) SEM | ↓ (≈68.6 ±5%) SEM | ↓ (≈57.3 ±5%) SEM |  |  |  | Kim et al. 2016 [54] |
| 0.05µM | 24hr | ↔ (≈96.6 ±5%) SEM |  |  |  |  |  | Cid-Diaz et al. 2017 [61] |
| 0.05µM | 48hr | ↓ (≈22.2 ±>10%) |  |  |  |  |  | Lee et al. 2022 [17] |
| 0.1µM | 3hr | ↓ (≈70.0 ±>10%) SEM |  |  |  |  |  | Xie et al. 2018 [s59] |
| 0.1µM | 6hr | ↓ (≈58.0 ±>10%) SEM |  |  |  |  |  | Xie et al. 2018 [s59] |
| 0.1µM | 12hr | ↓ (≈50.0 ±>10%) SEM |  |  |  |  |  | Xie et al. 2018 [s59] |
| 0.1µM | 24hr | ↓ (≈20.0 ±>10%) SEM |  |  |  |  |  | Xie et al. 2018 [s59] |
| 0.1µM | 192hr | ↓ (≈27.8 ±5%) SEM | ↓ (≈41.7 ±5%) SEM | ↓ (≈39.3 ±5%) SEM |  |  |  | Kim et al. 2016 [54] |
| 0.5µM | 24hr | ↔ (≈94.9 ±5%) SEM |  |  |  |  |  | Cid-Diaz et al. 2017 [61] |
| 1µM | 4hr | ↔ (≈87.8 ±5%) SEM |  |  |  |  |  | Otsuka et al. 2019 [s62] |
| 1µM | 6hr |  |  |  |  |  | ↓ (≈38.8 ±5%) SEM | Hong et al. 2019 [s48] |
| 1µM | 12hr | ↔ (≈77.7 ±>10%) |  | ↔ (≈88.8 ±>10%) |  |  | ↔ (≈74.1 ±5%) | Yeon et al. 2020 [s49] |
| 1µM | 24hr | ↓ (≈62.1 ±>10%) | ↓ (≈52.2 ±>10%) |  |  |  |  | Hsieh et al. 2019 [68] |
| 1µM | 24hr | ↓ (≈64.4 ±>10%) SEM | ↓ (≈56.2 ±>10%) SEM | ↓ (≈57.7 ±>10%) SEM |  | ↓ (≈56.8 ±>10%) SEM |  | Lu et al. 2013 [60] |
| 1µM | 24hr | ↔ (≈103.8 ±>10%) SEM |  |  |  |  |  | Murata et al. 2017 [s37] |
| 1µM | 24hr | ↓ (≈91.5 ±5%) SEM | ↓ (≈50.0 ±5%) SEM | ↓ (≈64.2 ±5%) SEM | ↓ (≈67.8 ±5%) SEM | ↓ (≈62.9 ±5%) SEM |  | Cid-Diaz et al. 2017 [61] |
| 1µM | 24hr | ↓ (≈67.3 ±>10%) |  | ↓ (≈73.0 ±>10%) |  |  |  | Yoshioka et al. 2023 [66] |
| 1µM | 24hr | ↓ (≈45.0 ±>10%) |  |  |  |  |  | Morano et al. 2018 [67] |
| 1µM | 48hr | ↓ (80% ±NR) |  |  |  |  |  | Han et al. 2020 [71] |
| 1µM | 48hr | ↓ (≈66.6 ±>10%) | ↓ (≈53.7 ±>10%) |  |  |  |  | Salvadori et al. 2024 [s51] |
| 1µM | 48hr | ↔ (≈100.0 ±>10%) |  |  |  |  |  | Archer-Lahlou et al. 2018 [76] |
| 1µM | 192hr | ↓ (≈18.0 ±5%) SEM | ↓ (≈34.3 ±5%) SEM | ↓ (≈31.1 ±5%) SEM |  |  |  | Kim et al. 2016 [54] |
| 2µM | 36hr | ↓ (≈69.6 ±5%) SEM |  |  |  |  |  | Yu et al. 2017 [s65] |
| 3µM | 24hr | ↓ (≈59.6 ±>10%) SEM |  | ↓ (≈69.0 ±>10%) SEM |  |  |  | Sakai et al. 2018 [s26] |
| 5µM | 24hr | ↓ VC |  | ↓ VC | ↓ VC |  | ↔ (≈86.0 ±>10%) | Kim et al. 2024 [78] |
| 5µM | 24hr | ↓ (≈84.7 ±>10%) | ↓ (≈70.9 ±>10%) | ↓ (≈85.1 ±>10%) |  |  |  | Kim et al. 2022 [22] |
| 5µM | 24hr | ↓ VC | ↓ VC | ↓ VC | ↓ VC |  | ↓ VC | Lee et al. 2021 [80] |
| 5µM | 24hr | ↓ (≈47.2 ±>10%) |  |  |  |  | ↓ VC | Son et al. 2017 [81] |
| 5µM | 24hr | ↔ (≈100.0 ±>10%) SEM | ↔ (≈82.7 ±>10%) SEM | ↔ (≈120.0 ±>10%) SEM | ↓ (≈86.2 ±>10%) SEM |  |  | Eo et al. 2020 [82] |
| 5µM | 24hr | ↔ (≈84.8 ±>10%) |  |  |  |  |  | Choi et al. 2024 [77] |
| 10µM | 24hr | ↓ (≈72.2 ±>10%) |  | ↓ (≈69.7 ±>10%) |  |  |  | Tsuchida et al. 2017 [51] |
| 10µM | 24hr | ↓ (≈30.3 ±>10%) | ↓ (≈30.3 ±>10%) |  |  |  |  | Kimira et al. 2023 [89] |
| 10µM | 24hr | ↓ (≈88.8 ±5%) | ↓ (≈85.0 ±5%) | ↓ (≈82.6 ±5%) |  |  |  | Son et al. 2024 [26] |
| 10µM | 24hr | ↓ (≈50.0 ±>10%) |  |  |  |  |  | Rahman et al. 2024 [90] |
| 10µM | 24hr | ↓ VC | ↓ VC | ↓ VC |  |  |  | Kim et al. 2021 [14] |
| 10µM | 24hr | ↓ (≈40.5 ±>10%) |  |  |  |  | ↓ (≈51.1 ±>10%) | Kim et al. 2022 [91] |
| 10µM | 24hr | ↓ (≈55.1 ±>10%) |  |  |  |  |  | Zhiyin et al. 2021 [92] |
| 10µM | 24hr | ↓ (≈55.5 ±5%) SEM |  |  |  |  |  | Wang et al. 2021 [18] |
| 10µM | 24hr | ↓ (≈76.1 ±5%) | ↓ (≈87.5 ±5%) | ↓ (≈87.5 ±5%) |  |  |  | Kim et al. 2023 [13] |
| 10µM | 24hr | ↓ (≈56.8 ±5%) |  |  |  |  |  | Shen et al. 2019 [28] |
| 10µM | 24hr |  |  | ↓ (≈31.8 ±5%) |  |  |  | Pansters et al. 2013 [95] |
| 10µM | 48hr | ↓ (≈68.0 ±>10%) |  |  |  |  |  | Kim et al. 2020 [94] |
| 10µM | 48hr | ↓ (≈50.0 ±>10%) |  |  |  |  |  | Massaccesi et al. 2016 [s28] |
| 10µM | 48hr | ↓ (≈81.3 ±5%) SEM |  |  |  |  |  | Gurjar et al. 2020 [96] |
| 50µM | 24hr | ↓ (≈62.2 ±5%) SEM |  |  |  |  |  | Cid-Diaz et al. 2017 [61] |
| 50µM | 24hr | ↓ (≈28.2 ±5%) SEM |  |  |  |  |  | Sun et a. 2024 [36] |
| 50µM | 48hr |  |  | **↓** (≈50.0 ±10%) | **↓** (≈73.8 ±10%) |  |  | Jeon et al. 2021 [107] |
| 100µM | 8hr | ↔ (≈93.7 ±5%) SEM | ↓ (≈66.6 ±5%) SEM |  |  |  |  | Wang et al. 2017 [s44] |
| 100µM | 24hr | ↓ (≈65.9 ±5%) | ↓ (≈56.8 ±5%) | ↓ (≈35.2 ±5%) | ↓ (≈63.6 ±5%) |  |  | Lee et al. 2019 [37] |
| 100µM | 24hr | ↓ (≈39.2-64.4 ±5%) SEM |  |  |  |  |  | Cid-Diaz et al. 2017 [61] |
| 100µM | 24hr | ↓ (≈64.5 ±5%) SEM |  |  |  |  |  | Kukreti et al. 2013 [s30] |
| 100µM | 24hr | ↑ (120.0 ±NR%) | ↓ (60.0 ±NR%) | ↓ (70.0 ±NR%) |  |  |  | Kim et al. 2023 [119] |
| 100µM | 24hr | ↓ (≈69.3 ±5%) | ↓ (≈69.3 ±5%) |  |  |  |  | Lee et al. 2024 [s23] |
| 100µM | 24hr | ↓ (≈38.3 ±5%) | ↓ (≈23.3 ±5%) | ↓ (≈43.3 ±5%) | ↓ (≈40.0 ±5%) |  |  | Hur er al. 2024 [29] |
| 100µM | 24hr | ↓ (≈64.4 ±5%) SEM |  | ↑ (≈148.0 ±5%) SEM | ↓ (≈77.0 ±5%) SEM |  |  | Nguyen et al. 2024 [40] |
| 100µM | 24hr | ↓ (≈52.0 ±5%) | ↔ (≈80.6 ±5%) |  |  |  |  | Jo et al. 2024 [42] |
| 100µM | 24hr | ↓ (≈56.0 ±5%) SEM | ↓ (≈40.0 ±>10%) SEM | ↓ (≈26.0 ±>10%) SEM | ↔ (≈104.0 ±>10%) SEM | ↓ (≈52.0 ±>10%) SEM | ↓ (≈62.9 ±>10%) SEM | Edwards et al. 2022 [s1] |
| 100µM | 24hr |  | ↓ (≈50.0 ±>10%) |  |  |  |  | Zhou et al. 2024 [s5] |
| 100µM | 24hr | ↓ (≈42.1 ±>10%) SEM | ↓ (≈84.2 ±>10%) SEM |  |  |  |  | Park et al. 2020 [35] |
| 100µM | 24hr | ↓ (≈66.6 ±NR%) |  |  |  |  |  | Choi et al. 2022 [43] |
| 100µM | 24hr | ↓ (≈76.4 ±>10%) | ↓ (≈73.5 ±>10%) | ↓ (≈61.7 ±>10%) | ↓ (≈85.2 ±>10%) |  |  | Kim et al. 2023 [39] |
| 100µM | 24hr | ↑ (≈126.0 ±NR%) |  | ↓ (≈51.4 ±NR%) | ↔ (≈100.0 ±NR%) |  |  | Ko et al. 2024 [44] |
| 100µM | 24hr | ↓ (≈74.0 ±5%) | ↓ (≈60.9 ±>10%) |  |  |  |  | Wang et al. 2022 [s3] |
| 100µM | 48hr | ↓ VC | ↓ VC | ↓ VC | ↓ VC |  |  | Cheon et al. 2024 [s8] |
| 100µM | 48hr | ↓ VC |  |  |  |  |  | You et al. 2023 [111] |
| 100µM | 48hr | ↓ (≈72.0 ±>10%) | ↓ (≈42.8 ±>10%) | ↓ (≈41.6 ±>10%) |  |  |  | Huang et al. 2023 [s12] |
| 100µM | 48hr | ↓ (≈38.7 ±5%) | ↓ (≈37.7 ±5%) | ↓ (≈48.9 ±>10%) | ↓ (≈20.8 ±5%) |  |  | Jang et al. 2024 [45] |
| 100µM | 48hr | ↓ (≈43.9 ±>10%) | ↓ (≈43.9 ±>10%) |  |  |  |  | Chen et al. 2019 [s14] |
| 100µM | 48hr | ↓ (≈44.1 ±5%) SEM | ↓ (≈74.4 ±5%) SEM |  |  |  |  | Jeong et al. 2024 [s13] |
| 150µM | 24hr | ↓ (≈63.6 ±5%) SEM |  | ↓ (≈77.7 ±5%) SEM |  |  |  | Gwag et al. 2013 [s18] |
| 200µM | 24hr | ↓ (≈63.2-68.7 ±>10%) | ↓ (≈63.6-77.5 ±>10%) |  |  |  |  | Men et al. 2024 [33] |
| 200µM | 24hr | ↓ (≈51.1 ±>10%) | ↓ (≈53.3 ±>10%) |  |  |  |  | Men et al. 2024 [48] |
| 200µM | 24hr | ↓ (≈72.0 ±>10%) SEM | ↓ (≈11.6 ±>10%) SEM |  |  |  |  | Kim et al. 2024 [32] |
| 200µM | 24hr | ↓ (≈47.2 ±5%) | ↓ (≈52.7 ±5%) |  |  |  |  | Wang et al. 2020 [s21] |
| 200µM | 24hr | ↓ (≈61.2 ±>10%) |  |  |  |  |  | Jiang et al. 2019 [49] |

Note: Column are reported as raw values (if available) or as estimates (indicated by ≈) of treatment group expressed as a percent of control ± the variability for the treated group. Variability is listed as SD unless noted with another reporting value (such as SEM). ? indicates the type of variability presented was unclear. “VC” indicates visual confirmation was used to describe the effect of dexamethasone. “Varied MT” indicates that multiple targets were assessed (transcript or isotope variants). “NM” indicates that relative assessments were *not measurable*.

**Abbreviations**: p-EBP1, eukaryotic translation initiation factor 4E-binding protein 1; p-mTOR, mechanistic/mammalian target of rapamycin; phosphorylated ribosomal protein S6.

***Table s6 Effect of dexamethasone-mediated atrophy on myotube mitochondrial biogenesis.***

| Concentration | Duration | pAMPK | Ppargc1a | PGC1α | Nrf1 | NRF1 | Tfam | TFAM | Sirt1 | SIRT1 | Reference |
| --- | --- | --- | --- | --- | --- | --- | --- | --- | --- | --- | --- |
| 1µM | 24hr | ↑ (≈150.0 ±5%) SEM |  |  |  |  |  |  |  |  | Liu et al. 2016 [12] |
| 1µM | 48hr |  | ↑ (≈270.0 ±>10%) |  |  |  |  |  |  |  | Archer-Lahlou et al. 2018 [76] |
| 10µM | 24hr |  |  | ↔ (≈106.8 ±5%) |  |  |  |  |  |  | Ulla et al. 2021 [87] |
| 10µM | 24hr |  | ↓ (≈48.9 ±5%) |  | ↓ (≈51.3 ±5%) |  | ↓ (≈43.8 ±5%) |  |  |  | Kim et al. 2021 [14] |
| 10µM | 24hr |  |  | ↓ (≈77.3 ±5%) SEM |  | ↓ (≈77.3 ±5%) SEM |  | (≈75.4 ±5%) SEM | ↓ (≈51.4 ±5%) SEM | ↓ (≈75.7 ±5%) SEM | Zhiyin et al. 2021 [92] |
| 10µM | 24hr |  |  | ↓ (≈54.5±10%) |  |  |  |  |  | ↓ (≈70.3 ±5%) | Shen et al. 2019 [28] |
| 25µM | 96hr | ↑ (≈186.0 ±10%) |  |  |  |  |  |  |  |  | Chen et al. 2024 [s33] |
| 50µM | 24hr | ↑ (≈170.0 ±5%) |  |  |  |  |  |  |  |  | Wang et al. 2021 [34] |
| 100µM | 24hr | ↑ (VC) |  |  |  |  |  |  |  |  | Li et al. 2023 [101] |
| 100µM | 24hr |  | ↑ (≈250.0 ±5%) SEM |  |  |  |  |  |  |  | Nguyen et al. 2024 [40] |
| 100µM | 24hr | ↔ (≈100.0 ±5%) SEM |  | ↓ (≈83.3 ±5%) SEM |  |  |  | ↓ (≈91.1 ±5%) SEM |  |  | Edwards et al. 2022 [s1] |
| 100µM | 24hr | ↑ (≈161.9 ±10%) |  |  |  |  |  |  |  |  | Jo et al. 2024 [42] |
| 100µM | 24hr | ↔ (≈65.6 ±5%) |  |  |  |  |  |  |  |  | Park et al. 2020 [35] |
| 100µM | 24hr |  | ↔ (≈110.0 ±10%) SEM |  |  |  | ↔ (≈88.2 ±10%) SEM |  |  |  | Ko et al. 2024 [44] |
| 100µM | 48hr |  |  | ↓ VC |  |  |  |  |  | ↓ VC | Cheon et al. 2024 [s8] |
| 200µM | 24hr |  |  | ↓ (≈73.9-78.0±10%) |  |  |  |  |  | ↓ (≈47.9-53.9 ±10%) | Men et al. 2024 [33] |
| 200µM | 24hr |  |  | ↓ (≈61.1 ±10%) SEM |  |  |  |  |  | ↓ (≈58.0 ±10%) | Men et al. 2024 [48] |
| 200µM | 24hr | ↓ (≈78.1 ±5%) |  |  |  |  |  |  |  |  | Jiang et al. 2019 [49] |

Note: Column are reported as raw values (if available) or as estimates (indicated by ≈) of treatment group expressed as a percent of control ± the variability for the treated group. Variability is listed as SD unless noted with another reporting value (such as SEM). ? indicates the type of variability presented was unclear. “VC” indicates visual confirmation was used to describe the effect of dexamethasone. “Varied MT” indicates that multiple targets were assessed (transcript or isotope variants). “NM” indicates that relative assessments were *not measurable*.

**Abbreviations**: AMPK, AMP-activated protein kinase; Nrf1, nuclear respiratory factor 1 (mRNA); NRF1, nuclear respiratory factor 1 (protein); Ppargc1a, peroxisome proliferator-activated receptor gamma coactivator 1 alpha (mRNA); PGC-1α, peroxisome proliferator-activated receptor gamma coactivator 1 alpha (protein); Sirt1, Sirtuin 1 (mRNA); SIRT1, Sirtuin 1 (protein); Tfam, mitochondrial transcription factor A (mRNA); TFAM, mitochondrial transcription factor A (protein).

***Table s7 Effect of dexamethasone-mediated atrophy on myotube mitochondrial content and function.***

| Concentration | Duration | Mitochondrial Staining | ATP | O2 Consumption | Ox Phos | Reference |
| --- | --- | --- | --- | --- | --- | --- |
| 1µM | 6hr |  | ↓ (≈62.1 ±5%) SEM |  |  | Liu et al. 2016 [12] |
| 1µM | 12hr |  | ↓ (≈46.6 ±5%) SEM |  |  | Liu et al. 2016 [12] |
| 1µM | 24hr |  | ↓ (≈46.6 ±5%) SEM | ↓ (≈57.9 ±5%) SEM |  | Liu et al. 2016 [12] |
| 5µM | 24hr |  |  | ↓ (≈63.4 ±5%) |  | Lee et al. 2021 [80] |
| 5µM | 24hr |  |  | ↓ (varied) |  | Lee et al. 2022 [21] |
| 10µM | 24hr | ↓ (≈82.0 ±5%) | ↓ (≈62.3 ±5%) |  |  | Son et al. 2024 [26] |
| 10µM | 24hr | ↓ (≈67.5 ±5%) SEM | ↓ (≈86.5 ±5%) SEM |  |  | Zhiyin et al. 2021 [92] |
| 10µM | 24hr | ↓ (≈71.4 ±5%) | ↓ (≈71.0 ±5%) | ↓ (≈79.5 ±10%) |  | Shen et al. 2019 [28] |
| 25µM | 24hr |  | ↓ (≈88.5 ±5%) | ↓ (≈79.5 ±10%) |  | Wang et al. 2021 [34] |
| 50µM | 3hr |  | ↓ (≈88.9 ±5%) | ↓ (≈83.3 ±5%) |  | Wang et al. 2021 [34] |
| 50µM | 6hr |  | ↓ (≈77.0 ±5%) | ↓ (≈64.3 ±5%) |  | Wang et al. 2021 [34] |
| 50µM | 12hr |  | ↓ (≈75.2 ±5%) | ↓ (≈61.3±5%) |  | Wang et al. 2021 [34] |
| 50µM | 24hr | ↓ (≈75.0 ±5%) | ↓ (≈76.1 ±5%) | ↓ (≈64.3 ±5%) | ↓ (≈69.7±5%) | Wang et al. 2021 [34] |
| 50µM | 24hr |  | ↓ (≈80.7 ±5%) | ↓ (≈63.6 ±5%) |  | Wang et al. 2021 [34] |
| 100µM | 24hr |  | ↓ (≈73.6 ±5%) | ↓ (≈56.8 ±10%) |  | Wang et al. 2021 [34] |
| 100µM | 24hr | ↓ (≈32.6 ±10%) SEM | ↓ (≈80.6 ±5%) SEM |  |  | Nguyen et al. 2024 [40] |
| 100µM | 24hr |  |  |  | ↑ (≈122.8 ±5%) SEM | Edwards et al. 2022 [s1] |
| 100µM | 24hr | ↓ (≈30.0 ±5%) SEM | ↓ (≈80.7 ±5%) SEM |  |  | Ko et al. 2024 [44] |
| 200µM | 24hr |  | ↓ (≈71.9 ±5%) | ↓ (≈63.6 ±5%) |  | Wang et al. 2021 [34] |
| 200µM | 24hr | ↓ VC | ↓ (≈29.7 ±5%) SEM |  |  | Kim et al. 2024 [32] |

Note: Column are reported as raw values (if available) or as estimates (indicated by ≈) of treatment group expressed as a percent of control ± the variability for the treated group. Variability is listed as SD unless noted with another reporting value (such as SEM). ? indicates the type of variability presented was unclear. “VC” indicates visual confirmation was used to describe the effect of dexamethasone. “Varied MT” indicates that multiple targets were assessed (transcript or isotope variants). “NM” indicates that relative assessments were *not measurable*.

**Abbreviations**: ATP, Adenosine triphosphate; Ox Phos, oxidative phosphorylation.

**Primary References (Main Text)**

1. Yaffe D, Saxel O. Serial passaging and differentiation of myogenic cells isolated from dystrophic mouse muscle. Nature. 1977;270:725–7. doi:10.1038/270725a0

2. Heitzer MD, Wolf IM, Sanchez ER, Witchel SF, DeFranco DB. Glucocorticoid receptor physiology. Rev Endocr Metab Disord. 2007;8:321–30. doi:10.1007/s11154-007-9059-8

3. Oakley RH, Cidlowski JA. The biology of the glucocorticoid receptor: new signaling mechanisms in health and disease. J Allergy Clin Immunol. 2013;132:1033–44. doi:10.1016/j.jaci.2013.09.007

4. Zhao W, Qin W, Pan J, Wu Y, Bauman WA, Cardozo C. Dependence of dexamethasone-induced Akt/FOXO1 signaling, upregulation of MAFbx, and protein catabolism upon the glucocorticoid receptor. Biochem Biophys Res Commun. 2009;378:668–72. doi:10.1016/j.bbrc.2008.11.123

5. Giorgino F, Pedrini MT, Matera L, Smith RJ. Specific increase in p85alpha expression in response to dexamethasone is associated with inhibition of insulin-like growth factor-I stimulated phosphatidylinositol 3-kinase activity in cultured muscle cells. J Biol Chem. 1997;272:7455–63. doi:10.1074/jbc.272.11.7455

6. Wang X, Hu J, Price SR. Inhibition of PI3-kinase signaling by glucocorticoids results in increased branched-chain amino acid degradation in renal epithelial cells. Am J Physiol Cell Physiol. 2007;292:C1874–9. doi:10.1152/ajpcell.00617.2006

7. Lee MK, Jeong HH, Kim MJ, Ryu H, Baek J, Lee B. Nutrients against Glucocorticoid-Induced Muscle Atrophy. Foods. 2022;11:687. doi:10.3390/foods11050687

8. Ma K, Mallidis C, Bhasin S, Mahabadi V, Artaza J, Gonzalez-Cadavid N, et al. Glucocorticoid-induced skeletal muscle atrophy is associated with upregulation of myostatin gene expression. Am J Physiol Endocrinol Metab. 2003;285:E363–71. doi:10.1152/ajpendo.00487.2002

9. Allen DL, Unterman TG. Regulation of myostatin expression and myoblast differentiation by FoxO and SMAD transcription factors. Am J Physiol Cell Physiol. 2007;292:C188–99. doi:10.1152/ajpcell.00542.2005

10. Xiao Q, Sun CC, Tang CF. Heme oxygenase-1: A potential therapeutic target for improving skeletal muscle atrophy. Exp Gerontol. 2023;184:112335. doi:10.1016/j.exger.2023.112335

11. Romanello V, Sandri M. The connection between the dynamic remodeling of the mitochondrial network and the regulation of muscle mass. Cell Mol Life Sci. 2021;78:1305–28. doi:10.1007/s00018-020-03662-0

12. Liu J, Peng Y, Wang X, Fan Y, Qin C, Shi L, et al. Mitochondrial Dysfunction Launches Dexamethasone-Induced Skeletal Muscle Atrophy via AMPK/FOXO3 Signaling. Mol Pharm. 2016;13:73–84. doi:10.1021/acs.molpharmaceut.5b00516

13. Kim JY, Kim HM, Kim JH, Guo S, Lee DH, Lim GM, et al. R.Br. and Rosmarinic Acid Attenuate Dexamethasone-Induced Muscle Atrophy in C2C12 Myotubes. Int J Mol Sci. 2023;24:doi:10.3390/ijms24031876

14. Kim R, Kim H, Im M, Park SK, Han HJ, An S, et al. BST204 Protects Dexamethasone-Induced Myotube Atrophy through the Upregulation of Myotube Formation and Mitochondrial Function. Int J Environ Res Public Health. 2021;18:2367. doi:10.3390/ijerph18052367

15. Li J, Chan MC, Yu Y, Bei Y, Chen P, Zhou Q, et al. miR-29b contributes to multiple types of muscle atrophy. Nat Commun. 2017;8:15201. doi:10.1038/ncomms15201

16. Cunningham JT, Rodgers JT, Arlow DH, Vazquez F, Mootha VK, Puigserver P. mTOR controls mitochondrial oxidative function through a YY1-PGC-1alpha transcriptional complex. Nature. 2007;450:736–40. doi:10.1038/nature06322

17. Lee CW, Chang YB, Park CW, Han SH, Suh HJ, Ahn Y. Protein Hydrolysate from Spirulina platensis Prevents Dexamethasone-Induced Atrophy via Akt/Foxo3 Signaling in C2C12 Myotubes. Mar Drugs. 2022;20:365. doi:10.3390/md20060365

18. Wang L, Jiao XF, Wu C, Li XQ, Sun HX, Shen XY, et al. Trimetazidine attenuates dexamethasone-induced muscle atrophy via inhibiting NLRP3/GSDMD pathway-mediated pyroptosis. Cell Death Discov. 2021;7:251. doi:10.1038/s41420-021-00648-0

19. Micheli L, Mitidieri E, Turnaturi C, Vanacore D, Ciampi C, Lucarini E, et al. Beneficial Effect of H2S-Releasing Molecules in an In Vitro Model of Sarcopenia: Relevance of Glucoraphanin. Int J Mol Sci. 2022;23:5955. doi:10.3390/ijms23115955

20. An DH, Lee CH, Kwon Y, Kim TH, Kim EJ, Jung JI, et al. Effects of Alnus japonica Hot Water Extract and Oregonin on Muscle Loss and Muscle Atrophy in C2C12 Murine Skeletal Muscle Cells. Pharmaceuticals (Basel). 2024;17:1661. doi:10.3390/ph17121661

21. Lee H, Kim YI, Kim MJ, Hahm JH, Seo HD, Ha TY, et al. Castor Oil Plant (Front Pharmacol. 2022;13:891762. doi:10.3389/fphar.2022.891762

22. Kim JY, Kim HM, Kim JH, Lee JH, Zhang K, Guo S, et al. Preventive effects of the butanol fraction of butanol fraction of Justicia produmbens L. against dexamethasone-induced muscle atrophy in C2C12 myotubes. Heliyon. 2022;8:e11597. doi:10.1016/j.heliyon.2022.e11597

23. Ma Z, Zhong Z, Zheng Z, Shi XM, Zhang W. Inhibition of glycogen synthase kinase-3β attenuates glucocorticoid-induced suppression of myogenic differentiation in vitro. PLoS One. 2014;9:e105528. doi:10.1371/journal.pone.0105528

24. Amarasiri RPGS, Hyun J, Lee SW, Kim J, Jeon YJ, Lee JS. Alcalase-Assisted Mytilus edulis Hydrolysate: A Nutritional Approach for Recovery from Muscle Atrophy. Mar Drugs. 2023;21:doi:10.3390/md21120623

25. Kurera MJMS, Nagahawatta DP, Liyanage NM, Jayawardhana HHAC, Dissanayake DS, Lee HG, et al. Exploring the Potential of Crassostrea nippona Hydrolysates as Dietary Supplements for Mitigating Dexamethasone-Induced Muscle Atrophy in C2C12 Cells. Mar Drugs. 2024;22:113. doi:10.3390/md22030113

26. Son RH, Kim MI, Kim HM, Guo S, Lee DH, Lim GM, et al. Potential of Lycii Radicis Cortex as an Ameliorative Agent for Skeletal Muscle Atrophy. Pharmaceuticals (Basel). 2024;17:462. doi:10.3390/ph17040462

27. Salucci S, Burattini S, Versari I, Bavelloni A, Bavelloni F, Curzi D, et al. Morpho-Functional Analyses Demonstrate That Tyrosol Rescues Dexamethasone-Induced Muscle Atrophy. J Funct Morphol Kinesiol. 2024;9:124. doi:10.3390/jfmk9030124

28. Shen S, Liao Q, Liu J, Pan R, Lee SM, Lin L. Myricanol rescues dexamethasone-induced muscle dysfunction via a sirtuin 1-dependent mechanism. J Cachexia Sarcopenia Muscle. 2019;10:429–44. doi:10.1002/jcsm.12393

29. Hur H, Kim HJ, Lee D, Jo C. Beef peptides mitigate skeletal muscle atrophy in C2C12 myotubes through protein degradation, protein synthesis, and the oxidative stress pathway. Food Funct. 2024;15:4564–74. doi:10.1039/d3fo03911k

30. Ma H, Jing Y, Zeng J, Ge J, Sun S, Cui R, et al. Human umbilical cord mesenchymal stem cell-derived exosomes ameliorate muscle atrophy via the miR-132-3p/FoxO3 axis. J Orthop Translat. 2024;49:23–36. doi:10.1016/j.jot.2024.08.005

31. Chen H, Ma J, Ma X. Administration of tauroursodeoxycholic acid attenuates dexamethasone-induced skeletal muscle atrophy. Biochem Biophys Res Commun. 2021;570:96–102. doi:10.1016/j.bbrc.2021.06.102

32. Kim A, Kim YR, Park SM, Lee H, Park M, Yi JM, et al. Jakyak-gamcho-tang, a decoction of Paeoniae Radix and Glycyrrhizae Radix et Rhizoma, ameliorates dexamethasone-induced muscle atrophy and muscle dysfunction. Phytomedicine. 2024;123:155057. doi:10.1016/j.phymed.2023.155057

33. Men X, Han X, Lee SJ, Oh G, Im JH, Bae KS, et al. Ginsenosides Rh1, Rg2, and Rg3 ameliorate dexamethasone-induced muscle atrophy in C2C12 myotubes. Food Sci Biotechnol. 2024;33:1233–43. doi:10.1007/s10068-023-01407-w

34. Wang M, Jiang R, Liu J, Xu X, Sun G, Zhao D, et al. 20(s)‑ginseonside‑Rg3 modulation of AMPK/FoxO3 signaling to attenuate mitochondrial dysfunction in a dexamethasone‑injured C2C12 myotube‑based model of skeletal atrophy. Mol Med Rep. 2021;23:doi:10.3892/mmr.2021.11945

35. Park SH, Oh J, Jo M, Kim JK, Kim DS, Kim HG, et al. Water Extract of Lotus Leaf Alleviates Dexamethasone-Induced Muscle Atrophy via Regulating Protein Metabolism-Related Pathways in Mice. Molecules. 2020;25:4592. doi:10.3390/molecules25204592

36. Sun Y, Wei X, Zhao T, Shi H, Hao X, Wang Y, et al. Oleanolic acid alleviates obesity-induced skeletal muscle atrophy via the PI3K/Akt signaling pathway. FEBS Open Bio. 2024;14:584–97. doi:10.1002/2211-5463.13780

37. Lee MK, Choi JW, Choi YH, Nam TJ. Protective Effect of Pyropia yezoensis Peptide on Dexamethasone-Induced Myotube Atrophy in C2C12 Myotubes. Mar Drugs. 2019;17:284. doi:10.3390/md17050284

38. Lee MK, Choi JW, Choi YH, Nam TJ. Protein Prevents Dexamethasone-Induced Myotube Atrophy in C2C12 Myotubes. Mar Drugs. 2018;16:doi:10.3390/md16120497

39. Kim NH, Lee JY, Kim CY. Protective Role of Ethanol Extract of Cibotium barometz (Cibotium Rhizome) against Dexamethasone-Induced Muscle Atrophy in C2C12 Myotubes. Int J Mol Sci. 2023;24: 14789. doi:10.3390/ijms241914798

40. Nguyen NB, Le TT, Kang SW, Cha KH, Choi S, Youn HY, et al. Cornflower Extract and Its Active Components Alleviate Dexamethasone-Induced Muscle Wasting by Targeting Cannabinoid Receptors and Modulating Gut Microbiota. Nutrients. 2024;16:1130. doi:10.3390/nu16081130

41. Han J, Choi SY, Choi RY, Park KW, Kang KY, Lee MK. Anti-muscle atrophy effect of fermented Tenebrio molitor larvae extract by modulating the PI3K-Akt-mTOR/FoxO3α pathway in mice treated with dexamethasone. Biomed Pharmacother. 2024;178:117266. doi:10.1016/j.biopha.2024.117266

42. Jo HS, Kim MJ, Amaya-Quiroz L, Yoon H, Han BK, Hong JY, et al. Suppressive Effects of Arriheuk Wheat Sprout Extract on Muscle Atrophy in Dexamethasone-Induced C2C12 Myotubes and a Mouse Model. J Med Food. 2024;27:1201–9. doi:10.1089/jmf.2024.k.0104

43. Choi RY, Kim BS, Ban EJ, Seo M, Lee JH, Kim IW. Mealworm Ethanol Extract Enhances Myogenic Differentiation and Alleviates Dexamethasone-Induced Muscle Atrophy in C2C12 Cells. Life (Basel). 2022;13:58. doi:10.3390/life13010058

44. Ko H, Bekele TT, Le TT, Cha KH, Kim S, Youn H-Y, et al. Identification of components from Aralia elata and their effects on muscle health and gut microbiota. Journal of Functional Foods. 2024;121:106384.

45. Jang JH, Joung JY, Pack SP, Oh NS. Preventive effect of fermented whey protein mediated by Lactobacillus gasseri IM13 via the PI3K/AKT/FOXO pathway in muscle atrophy. J Dairy Sci. 2024;107:2606–19. doi:10.3168/jds.2023-24027

46. Lee HY, Lee J, Lim H, Kim HY, Koo YS, Lim JS, et al. BNR17 Ameliorates Dexamethasone-Induced Muscle Loss in BALB/c Mice and C2C12 Myotubes. J Med Food. 2024;27:385–95. doi:10.1089/jmf.2023.K.0278

47. Chen C, Yang JS, Lu CC, Chiu YJ, Chen HC, Chung MI, et al. Effect of Quercetin on Dexamethasone-Induced C2C12 Skeletal Muscle Cell Injury. Molecules. 2020;25:3267. doi:10.3390/molecules25143267

48. Men X, Han X, La IJ, Lee SJ, Oh G, Im JH, et al. Ameliorative Effects of Fermented Red Ginseng Extract on Muscle Atrophy in Dexamethasone-Induced C2C12 Cell And Hind Limb-Immobilized C57BL/6J Mice. J Med Food. 2024;27:951–60. doi:10.1089/jmf.2024.k.0168

49. Jiang R, Wang M, Shi L, Zhou J, Ma R, Feng K, et al. Total Protein Facilitates Recovery from Dexamethasone-Induced Muscle Atrophy through the Activation of Glucose Consumption in C2C12 Myotubes. Biomed Res Int. 2019;2019:3719643. doi:10.1155/2019/3719643

50. Kim A, Kim J, Kim BY, Seo CS, Kim YR, Song KH, et al. Aquo-ethanolic extract of Lilii Bulbus attenuates dexamethasone-induced muscle loss and enhances muscle strength in experimental mice. Biomed Pharmacother. 2024;181:117658. doi:10.1016/j.biopha.2024.117658

51. Tsuchida W, Iwata M, Akimoto T, Matsuo S, Asai Y, Suzuki S. Heat Stress Modulates Both Anabolic and Catabolic Signaling Pathways Preventing Dexamethasone-Induced Muscle Atrophy In Vitro. J Cell Physiol. 2017;232:650–64. doi:10.1002/jcp.25609

52. Jia H, Yamashita T, Li X, Kato H. Laurel Attenuates Dexamethasone-Induced Skeletal Muscle Atrophy In Vitro and in a Rat Model. Nutrients. 2022;14:2029. doi:10.3390/nu14102029

53. Sultan KR, Henkel B, Terlou M, Haagsman HP. Quantification of hormone-induced atrophy of large myotubes from C2C12 and L6 cells: atrophy-inducible and atrophy-resistant C2C12 myotubes. Am J Physiol Cell Physiol. 2006;290:C650–9. doi:10.1152/ajpcell.00163.2005

54. Kim J, Park MY, Kim HK, Park Y, Whang KY. Cortisone and dexamethasone inhibit myogenesis by modulating the AKT/mTOR signaling pathway in C2C12. Biosci Biotechnol Biochem. 2016;80:2093–9. doi:10.1080/09168451.2016.1210502

55. Li Q, Kong ZD, Wang H, Gu HH, Chen Z, Li SG, et al. Jianpi Decoction Combined with Medroxyprogesterone Acetate Alleviates Cancer Cachexia and Prevents Muscle Atrophy by Directly Inhibiting E3 Ubiquitin Ligase. Chin J Integr Med. 2024;30:499–506. doi:10.1007/s11655-023-3702-4

56. Sun H, Gong Y, Qiu J, Chen Y, Ding F, Zhao Q. TRAF6 inhibition rescues dexamethasone-induced muscle atrophy. Int J Mol Sci. 2014;15:11126–41. doi:10.3390/ijms150611126

57. Kim J, Yang Y, Choi E, Lee S, Choi J. Effects of C-Peptide on Dexamethasone-Induced In Vitro and In Vivo Models as a Potential Therapeutic Agent for Muscle Atrophy. Int J Mol Sci. 2023;24:15433. doi:10.3390/ijms242015433

58. Son YH, Lee SJ, Lee KB, Lee JH, Jeong EM, Chung SG, et al. Dexamethasone downregulates caveolin-1 causing muscle atrophy via inhibited insulin signaling. J Endocrinol. 2015;225:27–37. doi:10.1530/JOE-14-0490

59. Menconi M, Gonnella P, Petkova V, Lecker S, Hasselgren PO. Dexamethasone and corticosterone induce similar, but not identical, muscle wasting responses in cultured L6 and C2C12 myotubes. J Cell Biochem. 2008;105:353–64. doi:10.1002/jcb.21833

60. Lu L, Wang DT, Shi Y, Yin Y, Wei LB, Zou YC, et al. Astragalus polysaccharide improves muscle atrophy from dexamethasone- and peroxide-induced injury in vitro. Int J Biol Macromol. 2013;61:7–16. doi:10.1016/j.ijbiomac.2013.06.027

61. Cid-Díaz T, Santos-Zas I, González-Sánchez J, Gurriarán-Rodríguez U, Mosteiro CS, Casabiell X, et al. Obestatin controls the ubiquitin-proteasome and autophagy-lysosome systems in glucocorticoid-induced muscle cell atrophy. J Cachexia Sarcopenia Muscle. 2017;8:974–90. doi:10.1002/jcsm.12222

62. Katsuki R, Sakata S, Nakao R, Oishi K, Nakamura Y. Lactobacillus curvatus CP2998 Prevents Dexamethasone-Induced Muscle Atrophy in C2C12 Myotubes. J Nutr Sci Vitaminol (Tokyo). 2019;65:455–8. doi:10.3177/jnsv.65.455

63. Le Bacquer O, Lanchais K, Combe K, Van Den Berghe L, Walrand S. Acute rimonabant treatment promotes protein synthesis in C2C12 myotubes through a CB1-independent mechanism. J Cell Physiol. 2021;236:2669–83. doi:10.1002/jcp.30034

64. Sawano S, Kobayashi Y, Maesawa S, Mizunoya W. Egg components reverse the atrophy induced by injury in skeletal muscles. Genes Cells. 2022;27:138–44. doi:10.1111/gtc.12915

65. Habibian JS, Bolino M, Qian A, Woolsey R, Quilici D, Petereit J, et al. Class I HDAC inhibitors attenuate dexamethasone-induced muscle atrophy via increased protein kinase C (PKC) delta phosphorylation. Cell Signal. 2023;110:110815. doi:10.1016/j.cellsig.2023.110815

66. Yoshioka Y, Oishi S, Onoda K, Shibata K, Miyoshi N. Diosgenin prevents dexamethasone-induced myotube atrophy in C2C12 cells. Arch Biochem Biophys. 2023;747:109759. doi:10.1016/j.abb.2023.109759

67. Morano M, Ronchi G, Nicolò V, Fornasari BE, Crosio A, Perroteau I, et al. Modulation of the Neuregulin 1/ErbB system after skeletal muscle denervation and reinnervation. Sci Rep. 2018;8:5047. doi:10.1038/s41598-018-23454-8

68. Hsieh SK, Lin HY, Chen CJ, Jhuo CF, Liao KY, Chen WY, et al. Promotion of myotube differentiation and attenuation of muscle atrophy in murine C2C12 myoblast cells treated with teaghrelin. Chem Biol Interact. 2020;315:108893. doi:10.1016/j.cbi.2019.108893

69. Han DS, Yang WS, Kao TW. Dexamethasone Treatment at the Myoblast Stage Enhanced C2C12 Myocyte Differentiation. Int J Med Sci. 2017;14:434–43. doi:10.7150/ijms.18427

70. Singh AK, Shree S, Chattopadhyay S, Kumar S, Gurjar A, Kushwaha S, et al. Small molecule adiponectin receptor agonist GTDF protects against skeletal muscle atrophy. Mol Cell Endocrinol. 2017;439:273–85. doi:10.1016/j.mce.2016.09.013

71. Han Y, Lee H, Li H, Ryu JH. Corylifol A from Psoralea corylifolia L. Enhnaces Myogenesis and Alleviates Muscle Atrophy. Int J Mol Sci. 2020;21:1571. doi:10.3390/ijms21051571

72. Hah Y-s, Lee WK, Lee S, Seo J-H, Kim JE, Choe Y-i, et al. Coumestrol attenuates dexamethasone-induced muscle atrophy via AMPK-FOXO1/3 signaling. Journal of Functional Foods. 2023;100: 105387

73. Hah YS, Lee WK, Lee S, Kim EJ, Lee JH, Lee SJ, et al. β-Sitosterol Attenuates Dexamethasone-Induced Muscle Atrophy via Regulating FoxO1-Dependent Signaling in C2C12 Cell and Mice Model. Nutrients. 2022;14:2894. doi:10.3390/nu14142894

74. Di Cesare Mannelli L, Micheli L, Lucarini E, Parisio C, Toti A, Tenci B, et al. Effects of the Combination of β-Hydroxy-β-Methyl Butyrate and R(+) Lipoic Acid in a Cellular Model of Sarcopenia. Molecules. 2020;25:2117. doi:10.3390/molecules25092117

75. Hah YS, Lee WK, Lee SJ, Lee SY, Seo JH, Kim EJ, et al. Rutin Prevents Dexamethasone-Induced Muscle Loss in C2C12 Myotube and Mouse Model by Controlling FOXO3-Dependent Signaling. Antioxidants (Basel). 2023;12:639. doi:10.3390/antiox12030639

76. Archer-Lahlou E, Lan C, Jagoe RT. Physiological culture conditions alter myotube morphology and responses to atrophy treatments: implications for in vitro research on muscle wasting. Physiol Rep. 2018;6:e13726. doi:10.14814/phy2.13726

77. Choi PG, Park SH, Jeong HY, Kim HS, Hahm JH, Seo HD, et al. Geniposide attenuates muscle atrophy via the inhibition of FoxO1 in senescence-accelerated mouse prone-8. Phytomedicine. 2024;123:155281. doi:10.1016/j.phymed.2023.155281

78. Kim YI, Lee H, Kim MJ, Jung CH, Kim YS, Ahn J. Identification of Peucedanum japonicum Thunb. extract components and their protective effects against dexamethasone-induced muscle atrophy. Phytomedicine. 2024;128:155449. doi:10.1016/j.phymed.2024.155449

79. Kim YI, Lee H, Nirmala FS, Seo HD, Ha TY, Jung CH, et al. Antioxidant Activity of Valeriana fauriei Protects against Dexamethasone-Induced Muscle Atrophy. Oxid Med Cell Longev. 2022;2022:3645431. doi:10.1155/2022/3645431

80. Lee H, Kim YI, Nirmala FS, Jeong HY, Seo HD, Ha TY, et al. Chrysanthemum zawadskil Herbich attenuates dexamethasone-induced muscle atrophy through the regulation of proteostasis and mitochondrial function. Biomed Pharmacother. 2021;136:111226. doi:10.1016/j.biopha.2021.111226

81. Son YH, Jang EJ, Kim YW, Lee JH. Sulforaphane prevents dexamethasone-induced muscle atrophy via regulation of the Akt/Foxo1 axis in C2C12 myotubes. Biomed Pharmacother. 2017;95:1486–92. doi:10.1016/j.biopha.2017.09.002

82. Eo H, Reed CH, Valentine RJ. Imoxin prevents dexamethasone-induced promotion of muscle-specific E3 ubiquitin ligases and stimulates anabolic signaling in C2C12 myotubes. Biomed Pharmacother. 2020;128:110238. doi:10.1016/j.biopha.2020.110238

83. Gan M, Ma J, Chen J, Chen L, Zhang S, Zhao Y, et al. miR-222 Is Involved in the Amelioration Effect of Genistein on Dexamethasone-Induced Skeletal Muscle Atrophy. Nutrients. 2022;14:1861. doi:10.3390/nu14091861

84. Lee JY, Lee M, Lee DH, Lee YH, Lee BW, Kang ES, et al. Protective Effect of Delta-Like 1 Homolog Against Muscular Atrophy in a Mouse Model. Endocrinol Metab (Seoul). 2022;37:684–97. doi:10.3803/EnM.2022.1446

85. McClung JM, Judge AR, Powers SK, Yan Z. p38 MAPK links oxidative stress to autophagy-related gene expression in cachectic muscle wasting. Am J Physiol Cell Physiol. 2010;298:C542–9. doi:10.1152/ajpcell.00192.2009

86. Bowen TS, Adams V, Werner S, Fischer T, Vinke P, Brogger MN, et al. Small-molecule inhibition of MuRF1 attenuates skeletal muscle atrophy and dysfunction in cardiac cachexia. J Cachexia Sarcopenia Muscle. 2017;8:939–53. doi:10.1002/jcsm.12233

87. Ulla A, Uchida T, Miki Y, Sugiura K, Higashitani A, Kobayashi T, et al. Morin attenuates dexamethasone-mediated oxidative stress and atrophy in mouse C2C12 skeletal myotubes. Arch Biochem Biophys. 2021;704:108873. doi:10.1016/j.abb.2021.108873

88. Lee Y, Kim HM, Kim JH, Lee JH, Zhang KX, Gao EM, et al. Chemical constituents of the Ajuga multiflora bunge and their protective effects on dexamethasone-induced muscle atrophy in C2C12 myotubes. Nat Prod Res. 2023;37:1978–85. doi:10.1080/14786419.2022.2115491

89. Kimira Y, Osawa K, Osawa Y, Mano H. Preventive Effects of Collagen-Derived Dipeptide Prolyl-Hydroxyproline against Dexamethasone-Induced Muscle Atrophy in Mouse C2C12 Skeletal Myotubes. Biomolecules. 2023;13:1617. doi:10.3390/biom13111617

90. Rahman MM, Ulla A, Moriwaki H, Yasukawa Y, Uchida T, Nikawa T. Muscle-Protective Effect of Carnosine against Dexamethasone-Induced Muscle Atrophy in C2C12 Myotube. J Nutr Sci Vitaminol (Tokyo). 2024;70:219–27. doi:10.3177/jnsv.70.219

91. Kim HJ, Kim SW, Lee SH, Jung DW, Williams DR. Inhibiting 5-lipoxygenase prevents skeletal muscle atrophy by targeting organogenesis signalling and insulin-like growth factor-1. J Cachexia Sarcopenia Muscle. 2022;13:3062–77. doi:10.1002/jcsm.13092

92. Zhiyin L, Jinliang C, Qiunan C, Yunfei Y, Qian X. Fucoxanthin rescues dexamethasone induced C2C12 myotubes atrophy. Biomed Pharmacother. 2021;139:111590. doi:10.1016/j.biopha.2021.111590

93. Yang Y, Yang X, Huang Y, Liu S, Niu Y, Fu L. Resistance exercise alleviates dexamethasone-induced muscle atrophy via Sestrin2/MSTN pathway in C57BL/6J mice. Exp Cell Res. 2023;432:113779. doi:10.1016/j.yexcr.2023.113779

94. Kim H, Cho SC, Jeong HJ, Lee HY, Jeong MH, Pyun JH, et al. Indoprofen prevents muscle wasting in aged mice through activation of PDK1/AKT pathway. J Cachexia Sarcopenia Muscle. 2020;11:1070–88. doi:10.1002/jcsm.12558

95. Pansters NA, Langen RC, Wouters EF, Schols AM. Synergistic stimulation of myogenesis by glucocorticoid and IGF-I signaling. J Appl Physiol (1985). 2013;114:1329–39. doi:10.1152/japplphysiol.00503.2012

96. Gurjar AA, Kushwaha S, Chattopadhyay S, Das N, Pal S, China SP, et al. Long acting GLP-1 analog liraglutide ameliorates skeletal muscle atrophy in rodents. Metabolism. 2020;103:154044. doi:10.1016/j.metabol.2019.154044

97. Park SY, Liu S, Carbajal EP, Wosczyna M, Costa M, Sun H. Hexavalent chromium inhibits myogenic differentiation and induces myotube atrophy. Toxicol Appl Pharmacol. 2023;477:116693. doi:10.1016/j.taap.2023.116693

98. Hyun J, Kang SI, Lee SW, Amarasiri RPGS, Nagahawatta DP, Roh Y, et al. Exploring the Potential of Olive Flounder Processing By-Products as a Source of Functional Ingredients for Muscle Enhancement. Antioxidants (Basel). 2023;12:1755. doi:10.3390/antiox12091755

99. Aguilar-Agon KW, Capel AJ, Fleming JW, Player DJ, Martin NRW, Lewis MP. Mechanical loading of tissue engineered skeletal muscle prevents dexamethasone induced myotube atrophy. J Muscle Res Cell Motil. 2021;42:149–59. doi:10.1007/s10974-020-09589-0

100. Nguyen KH, Ito S, Maeyama S, Schaffer SW, Murakami S, Ito T. In Vivo and In Vitro Study of N-Methyltaurine on Pharmacokinetics and Antimuscle Atrophic Effects in Mice. ACS Omega. 2020;5:11241–6. doi:10.1021/acsomega.0c01588

101. Li IC, Lu TY, Lin TW, Chen AY, Chu HT, Chen YL, et al. Hispidin-enriched Sanghuangporus sanghuang mycelia SS-MN4 ameliorate disuse atrophy while improving muscle endurance. J Cachexia Sarcopenia Muscle. 2023;14:2226–38. doi:10.1002/jcsm.13307

102. Yoo A, Kim JI, Lee H, Nirmala FS, Hahm JH, Seo HD, et al. Gromwell ameliorates glucocorticoid-induced muscle atrophy through the regulation of Akt/mTOR pathway. Chin Med. 2024;19:20. doi:10.1186/s13020-024-00890-5

103. Li Z, Liu C, Li S, Li T, Li Y, Wang N, et al. BMSC-Derived Exosomes Inhibit Dexamethasone-Induced Muscle Atrophy. Front Endocrinol (Lausanne). 2021;12:681267. doi:10.3389/fendo.2021.681267

104. Li X, Zhu Y, Zhang H, Ma G, Wu G, Xiang A, et al. MicroRNA-106a-5p Inhibited C2C12 Myogenesis via Targeting PIK3R1 and Modulating the PI3K/AKT Signaling. Genes (Basel). 2018;9:333. doi:10.3390/genes9070333

105. Liu Q, Yuan W, Yan Y, Jin B, You M, Liu T, et al. Identification of a novel small-molecule inhibitor of miR-29b attenuates muscle atrophy. Mol Ther Nucleic Acids. 2023;31:527–40. doi:10.1016/j.omtn.2023.02.003

106. Kim TY, Park KT, Choung SY. Codonopsis lanceolata and its active component Tangshenoside I ameliorate skeletal muscle atrophy via regulating the PI3K/Akt and SIRT1/PGC-1α pathways. Phytomedicine. 2022;100:154058. doi:10.1016/j.phymed.2022.154058

107. Jeon SH, Choung SY. Oyster Hydrolysates Attenuate Muscle Atrophy via Regulating Protein Turnover and Mitochondria Biogenesis in C2C12 Cell and Immobilized Mice. Nutrients. 2021;13:4385. doi:10.3390/nu13124385

108. Liang D, Wang D, Zheng X, Xiang H, Liu S, Yu C, et al. Aerobic plus resistance exercise attenuates skeletal muscle atrophy induced by dexamethasone through the HDAC4/FoxO3a pathway. Cell Signal. 2024;111581. doi:10.1016/j.cellsig.2024.111581

109. Oelkrug C, Horn K, Makert GR, Schubert A. Novel in vitro platform to investigate myotube atrophy. Anticancer Res. 2015;35:2085–91.

110. Maier MC, Nankervis S, Wallace ME, Develyn T, Myers MA. Dexamethasone leads to Zn2+ accumulation and increased unbound Zn2+ in C2C12 muscle and 3T3-L1 adipose cells. J Cell Biochem. 2023;124:409–20. doi:10.1002/jcb.30376

111. You CL, Lee SJ, Lee J, Vuong TA, Lee HY, Jeong SY, et al. upregulates muscle regeneration and augments function through muscle oxidative metabolism. Int J Biol Sci. 2023;19:4898–914. doi:10.7150/ijbs.84970

112. Sinam IS, Chanda D, Thoudam T, Kim MJ, Kim BG, Kang HJ, et al. Pyruvate dehydrogenase kinase 4 promotes ubiquitin-proteasome system-dependent muscle atrophy. J Cachexia Sarcopenia Muscle. 2022;13:3122–36. doi:10.1002/jcsm.13100

113. Uozumi Y, Ito T, Hoshino Y, Mohri T, Maeda M, Takahashi K, et al. Myogenic differentiation induces taurine transporter in association with taurine-mediated cytoprotection in skeletal muscles. Biochem J. 2006;394:699–706. doi:10.1042/BJ20051303

114. Van Pelt DW, Confides AL, Judge AR, Vanderklish PW, Dupont-Versteegden EE. Cold shock protein RBM3 attenuates atrophy and induces hypertrophy in skeletal muscle. J Muscle Res Cell Motil. 2018;39:35–40. doi:10.1007/s10974-018-9496-x

115. Qiu J, Wang L, Wang Y, Zhang Q, Ma W, Fang Q, et al. MicroRNA351 targeting TRAF6 alleviates dexamethasone-induced myotube atrophy. J Thorac Dis. 2018;10:6238–46. doi:10.21037/jtd.2018.10.88

116. Reinoso-Sánchez JF, Baroli G, Duranti G, Scaricamazza S, Sabatini S, Valle C, et al. Emerging Role for Linear and Circular Spermine Oxidase RNAs in Skeletal Muscle Physiopathology. Int J Mol Sci. 2020;21:doi:10.3390/ijms21218227

117. Ozaki Y, Ohashi K, Otaka N, Ogawa H, Kawanishi H, Takikawa T, et al. Neuron-derived neurotrophic factor protects against dexamethasone-induced skeletal muscle atrophy. Biochem Biophys Res Commun. 2022;593:5–12. doi:10.1016/j.bbrc.2022.01.028

118. Lee J, Kang M, Yoo J, Lee S, Yun B, Kim JN, et al. JY02 Ameliorates Sarcopenia by Anti-Atrophic Effects in a Dexamethasone-Induced Cellular and Murine Model. J Microbiol Biotechnol. 2023;33:915–25. doi:10.4014/jmb.2303.03001

119. Kim R, Kim JW, Choi H, Oh JE, Kim TH, Go GY, et al. Ginsenoside Rg5 promotes muscle regeneration via p38MAPK and Akt/mTOR signaling. J Ginseng Res. 2023;47:726–34. doi:10.1016/j.jgr.2023.06.004

120. Koo GB, Kwon HO, Kim JH, Lee SH, Shim SL, Jang KH. Protective Effects of Cervus elaphus and Eucommia ulmoides Mixture (KGC01CE) on Muscle Loss and Function in Aged Rats. Curr Issues Mol Biol. 2024;46:11190–206. doi:10.3390/cimb46100664

**Supplemental References**

s1. Edwards SJ, Carter S, Nicholson T, Allen SL, Morgan PT, Jones SW, et al. (-)-Epicatechin and its colonic metabolite hippuric acid protect against dexamethasone-induced atrophy in skeletal muscle cells. J Nutr Biochem. 2022;110:109150. doi:10.1016/j.jnutbio.2022.109150

s2. Kim H, Jang M, Park R, Jo D, Choi I, Choe J, et al. Conessine Treatment Reduces Dexamethasone-Induced Muscle Atrophy by Regulating MuRF1 and Atrogin-1 Expression. J Microbiol Biotechnol. 2018;28:520–6. doi:10.4014/jmb.1711.11009

s3. Wang P, Kang SY, Kim SJ, Park YK, Jung HW. Monotropein Improves Dexamethasone-Induced Muscle Atrophy via the AKT/mTOR/FOXO3a Signaling Pathways. Nutrients. 2022;14:1859. doi:10.3390/nu14091859

s4. Kang M, Yoo J, Lee J, Lee S, Yun B, Song M, et al. Dietary supplementation with Lacticaseibacillus rhamnosus IDCC3201 alleviates sarcopenia by modulating the gut microbiota and metabolites in dexamethasone-induced models. Food Funct. 2024;15:4936–53. doi:10.1039/d3fo05420a

s5. Zhou X, Xu S, Zhang Z, Tang M, Meng Z, Peng Z, et al. Gouqi-derived nanovesicles (GqDNVs) inhibited dexamethasone-induced muscle atrophy associating with AMPK/SIRT1/PGC1α signaling pathway. J Nanobiotechnology. 2024;22:276. doi:10.1186/s12951-024-02563-9

s6. Go H, Sung NJ, Choi J, Kim L, Park EJ. 6'-sialyllactose prevents dexamethasone-induced muscle atrophy by controlling the muscle protein degradation pathway. Biochem Biophys Res Commun. 2024;736:150892. doi:10.1016/j.bbrc.2024.150892

s7. Lee MK, Kim YM, Kim IH, Choi YH, Nam TJ. Pyropia yezoensis peptide PYP1‑5 protects against dexamethasone‑induced muscle atrophy through the downregulation of atrogin1/MAFbx and MuRF1 in mouse C2C12 myotubes. Mol Med Rep. 2017;15:3507–14. doi:10.3892/mmr.2017.6443

s8. Cheon YH, Lee CH, Chung CH, Kim JY, Lee MS. Vigeo Promotes Myotube Differentiation and Protects Dexamethasone-Induced Skeletal Muscle Atrophy via Regulating the Protein Degradation, AKT/mTOR, and AMPK/Sirt-1/PGC1α Signaling Pathway In Vitro and In Vivo. Nutrients. 2024;16:2687. doi:10.3390/nu16162687

s9. Rossi S, Stoppani E, Martinet W, Bonetto A, Costelli P, Giuliani R, et al. The cytosolic sialidase Neu2 is degraded by autophagy during myoblast atrophy. Biochim Biophys Acta. 2009;1790:817–28. doi:10.1016/j.bbagen.2009.04.006

s10. Chang JS, Kong ID. Irisin prevents dexamethasone-induced atrophy in C2C12 myotubes. Pflugers Arch. 2020;472:495–502. doi:10.1007/s00424-020-02367-4

s11. Li Y, Shi H, Chen R, Zhou S, Lei S, She Y. Role of miRNAs and lncRNAs in dexamethasone-induced myotube atrophy. Exp Ther Med. 2021;21:146. doi:10.3892/etm.2020.9577

s12. Huang M, Yan Y, Deng Z, Zhou L, She M, Yang Y, et al. Saikosaponin A and D attenuate skeletal muscle atrophy in chronic kidney disease by reducing oxidative stress through activation of PI3K/AKT/Nrf2 pathway. Phytomedicine. 2023;114:154766. doi:10.1016/j.phymed.2023.154766

s13. Jeong YJ, Kim JH, Jung YJ, Kwak MS, Sung MH, Imm JY. KL-Biome (Postbiotic Formulation of Lactiplantibacills plantarum KM2) Improves Dexamethasone-Induced Muscle Atrophy in Mice. Int J Mol Sci. 2024;25:7499. doi:10.3390/ijms25137499

s14. Chen L, Wan L, Huo Y, Huang J, Li J, Lu J, et al. Matrine improves skeletal muscle atrophy by inhibiting E3 ubiquitin ligases and activating the Akt/mTOR/FoxO3α signaling pathway in C2C12 myotubes and mice. Oncol Rep. 2019;42:479–94. doi:10.3892/or.2019.7205

s15. Yoon JH, Lee SM, Lee Y, Kim MJ, Yang JW, Choi JY, et al. Alverine citrate promotes myogenic differentiation and ameliorates muscle atrophy. Biochem Biophys Res Commun. 2022;586:157–62. doi:10.1016/j.bbrc.2021.11.076

s16. Nakagawara K, Takeuchi C, Ishige K. 5'-CMP and 5'-UMP alleviate dexamethasone-induced muscular atrophy in C2C12 myotubes. Biochem Biophys Rep. 2023;34:101460. doi:10.1016/j.bbrep.2023.101460

s17. Wang BY, Hsiao AW, Shiu HT, Wong N, Wang AY, Lee CW, et al. Mesenchymal stem cells alleviate dexamethasone-induced muscle atrophy in mice and the involvement of ERK1/2 signalling pathway. Stem Cell Res Ther. 2023;14:195. doi:10.1186/s13287-023-03418-0

s18. Gwag T, Park K, Kim E, Son C, Park J, Nikawa T, et al. Inhibition of C2C12 myotube atrophy by a novel HSP70 inducer, celastrol, via activation of Akt1 and ERK1/2 pathways. Arch Biochem Biophys. 2013;537:21–30. doi:10.1016/j.abb.2013.06.006

s19. Bae S, Mai VH, Mun S, Dong D, Han K, Park S, et al. Lonafarnib Protects Against Muscle Atrophy Induced by Dexamethasone. J Cachexia Sarcopenia Muscle. 2025;16:e13665. doi:10.1002/jcsm.13665

s20. Kwak MK, Ha ES, Lee J, Choi YM, Kim BJ, Hong EG. C-C motif chemokine ligand 2 promotes myogenesis of myoblasts via the AKT-mTOR pathway. Aging (Albany NY). 2022;14:9860–76. doi:10.18632/aging.204451

s21. Wang M, Ren J, Chen X, Liu J, Xu X, Li X, et al. 20(S)-ginsenoside Rg3 promotes myoblast differentiation and protects against myotube atrophy via regulation of the Akt/mTOR/FoxO3 pathway. Biochem Pharmacol. 2020;180:114145. doi:10.1016/j.bcp.2020.114145

s22. Han Z, Chang C, Zhu W, Zhang Y, Zheng J, Kang X, et al. Role of SIRT2 in regulating the dexamethasone-activated autophagy pathway in skeletal muscle atrophy. Biochem Cell Biol. 2021;99:562–9. doi:10.1139/bcb-2020-0445

s23. Lee HJ, Kim D, Do K, Yang CB, Jeon SW, Jang A. Effects of Horse Meat Hydrolysate on Oxidative Stress, Proinflammatory Cytokines, and the Ubiquitin-Proteasomal System of C2C12 Cells. Food Sci Anim Resour. 2024;44:132–45. doi:10.5851/kosfa.2023.e65

s24. Clarke BA, Drujan D, Willis MS, Murphy LO, Corpina RA, Burova E, et al. The E3 Ligase MuRF1 degrades myosin heavy chain protein in dexamethasone-treated skeletal muscle. Cell Metab. 2007;6:376–85. doi:10.1016/j.cmet.2007.09.009

s25. Kang J, Kim S, Lee Y, Oh J, Yoon Y. Effects on Goat Meat Extracts on α-Glucosidase Inhibitory Activity, Expression of Bcl-2-Associated X (BAX), p53, and p21 in Cell Line and Expression of Atrogin-1, Muscle Atrophy F-Box (MAFbx), Muscle RING-Finger Protein-1 (MuRF-1), and Myosin Heavy Chain-7 (MYH-7) in C2C12 Myoblsts. Food Sci Anim Resour. 2023;43:359–73. doi:10.5851/kosfa.2023.e6

s26. Sakai H, Kimura M, Tsukimura Y, Yabe S, Isa Y, Kai Y, et al. Dexamethasone exacerbates cisplatin-induced muscle atrophy. Clin Exp Pharmacol Physiol. 2019;46:19–28. doi:10.1111/1440-1681.13024

s27. Allen DL, Loh AS. Posttranscriptional mechanisms involving microRNA-27a and b contribute to fast-specific and glucocorticoid-mediated myostatin expression in skeletal muscle. Am J Physiol Cell Physiol. 2011;300:C124–37. doi:10.1152/ajpcell.00142.2010

s28. Massaccesi L, Goi G, Tringali C, Barassi A, Venerando B, Papini N. Dexamethasone-Induced Skeletal Muscle Atrophy Increases O-GlcNAcylation in C2C12 Cells. J Cell Biochem. 2016;117:1833–42. doi:10.1002/jcb.25483

s29. Proserpio V, Fittipaldi R, Ryall JG, Sartorelli V, Caretti G. The methyltransferase SMYD3 mediates the recruitment of transcriptional cofactors at the myostatin and c-Met genes and regulates skeletal muscle atrophy. Genes Dev. 2013;27:1299–312. doi:10.1101/gad.217240.113

s30. Kukreti H, Amuthavalli K, Harikumar A, Sathiyamoorthy S, Feng PZ, Anantharaj R, et al. Muscle-specific microRNA1 (miR1) targets heat shock protein 70 (HSP70) during dexamethasone-mediated atrophy. J Biol Chem. 2013;288:6663–78. doi:10.1074/jbc.M112.390369

s31. Krawiec BJ, Nystrom GJ, Frost RA, Jefferson LS, Lang CH. AMP-activated protein kinase agonists increase mRNA content of the muscle-specific ubiquitin ligases MAFbx and MuRF1 in C2C12 cells. Am J Physiol Endocrinol Metab. 2007;292:E1555–67. doi:10.1152/ajpendo.00622.2006

s32. Yamamoto D, Ikeshita N, Matsubara T, Tasaki H, Herningtyas EH, Toda K, et al. GHRP-2, a GHS-R agonist, directly acts on myocytes to attenuate the dexamethasone-induced expressions of muscle-specific ubiquitin ligases, Atrogin-1 and MuRF1. Life Sci. 2008;82:460–6. doi:10.1016/j.lfs.2007.11.019

s33. Verhees KJ, Schols AM, Kelders MC, Op den Kamp CM, van der Velden JL, Langen RC. Glycogen synthase kinase-3β is required for the induction of skeletal muscle atrophy. Am J Physiol Cell Physiol. 2011;301:C995–C1007. doi:10.1152/ajpcell.00520.2010

s34. Tomiya S, Tamura Y, Kouzaki K, Kotani T, Wakabayashi Y, Noda M, et al. Cast immobilization of hindlimb upregulates sarcolipin expression in atrophied skeletal muscles and increases thermogenesis in C57BL/6J mice. Am J Physiol Regul Integr Comp Physiol. 2019;317:R649–R61. doi:10.1152/ajpregu.00118.2019

s35. Kondo T, Ishida T, Ye K, Muraguchi M, Tanimura Y, Yoshida M, et al. Suppressive effects of processed aconite root on dexamethasone-induced muscle ring finger protein-1 expression and its active ingredients. J Nat Med. 2022;76:594–604. doi:10.1007/s11418-022-01606-5

s36. Cerquone Perpetuini A, Re Cecconi AD, Chiappa M, Martinelli GB, Fuoco C, Desiderio G, et al. Group I Paks support muscle regeneration and counteract cancer-associated muscle atrophy. J Cachexia Sarcopenia Muscle. 2018;9:727–46. doi:10.1002/jcsm.12303

s37. Murata M, Nonaka H, Komatsu S, Goto M, Morozumi M, Yamada S, et al. Delphinidin Prevents Muscle Atrophy and Upregulates miR-23a Expression. J Agric Food Chem. 2017;65:45–50. doi:10.1021/acs.jafc.6b03661

s38. Sun LJ, Sun YN, Chen SJ, Liu S, Jiang GR. Resveratrol attenuates skeletal muscle atrophy induced by chronic kidney disease via MuRF1 signaling pathway. Biochem Biophys Res Commun. 2017;487:83–9. doi:10.1016/j.bbrc.2017.04.022

s39. Yoshioka Y, Kubota Y, Samukawa Y, Yamashita Y, Ashida H. Glabridin inhibits dexamethasone-induced muscle atrophy. Arch Biochem Biophys. 2019;664:157–66. doi:10.1016/j.abb.2019.02.006

s40. Murata M, Shimizu Y, Marugame Y, Nezu A, Fujino K, Yamada S, et al. EGCG down-regulates MuRF1 expression through 67-kDa laminin receptor and the receptor signaling is amplified by eriodictyol. J Nat Med. 2020;74:673–9. doi:10.1007/s11418-020-01417-6

s41. Katsuki R, Shiraishi T, Sakata S, Hirota T, Nakamura Y, Yokota SI. Inhibitory Effect of the Glycerophosphate Moiety of Lipoteichoic Acid from Lactic Acid Bacteria on Dexamethasone-Induced Atrogin-1 Expression in C2C12 Myotubes. J Nutr Sci Vitaminol (Tokyo). 2021;67:351–7. doi:10.3177/jnsv.67.351

s42. Yoshioka Y, Imi Y, Kawabata K, Shibata K, Terao J, Miyoshi N. Theophylline Prevents Dexamethasone-Induced Atrophy in C2C12 Myotubes. J Nutr Sci Vitaminol (Tokyo). 2023;69:284–91. doi:10.3177/jnsv.69.284

s43. Shen H, Liu T, Fu L, Zhao S, Fan B, Cao J, et al. Identification of microRNAs involved in dexamethasone-induced muscle atrophy. Mol Cell Biochem. 2013;381:105–13. doi:10.1007/s11010-013-1692-9

s44. Wang XJ, Xiao JJ, Liu L, Jiao HC, Lin H. Excessive glucocorticoid-induced muscle MuRF1 overexpression is independent of Akt/FoXO1 pathway. Biosci Rep. 2017;37:BSR20171056. doi:10.1042/BSR20171056

s45. Chen YW, Li TJ, Wang LC, Yang BH, Chen YL, Chen CC, et al. Prevention of Muscle Atrophy by Low-Molecular-Weight Fraction from Hirsutella sinesnsis Mycelium. Curr Issues Mol Biol. 2024;46:14033–44. doi:10.3390/cimb46120839

s46. Brenmoehl J, Hoeflich A. Dual control of mitochondrial biogenesis by sirtuin 1 and sirtuin 3. Mitochondrion. 2013;13:755–61. doi:10.1016/j.mito.2013.04.002

s47. Chen K, Gao P, Li Z, Dai A, Yang M, Chen S, et al. Forkhead Box O Signaling Pathway in Skeletal Muscle Atrophy. Am J Pathol. 2022;192:1648–57. doi:10.1016/j.ajpath.2022.09.003

s48. Hong Y, Lee JH, Jeong KW, Choi CS, Jun HS. Amelioration of muscle wasting by glucagon-like peptide-1 receptor agonist in muscle atrophy. J Cachexia Sarcopenia Muscle. 2019;10:903–18. doi:10.1002/jcsm.12434

s49. Yeon M, Choi H, Jun HS. Preventive Effects of Schisandrin A, A Bioactive Component of Schisandrin A, A Bioactive Component of Schisandra chinensis, on Dexamethasone-Induced Muscle Atrophy. Nutrients. 2020;12:1255. doi:10.3390/nu12051255

s50. Adhikary S, Choudhary D, Tripathi AK, Karvande A, Ahmad N, Kothari P, et al. FGF-2 targets sclerostin in bone and myostatin in skeletal muscle to mitigate the deleterious effects of glucocorticoid on musculoskeletal degradation. Life Sci. 2019;229:261–76. doi:10.1016/j.lfs.2019.05.022

s51. Salvadori L, Paiella M, Castiglioni B, Belladonna ML, Manenti T, Ercolani C, et al. Equisetum arvense standardized dried extract hinders age-related osteosarcopenia. Biomed Pharmacother. 2024;174:116517. doi:10.1016/j.biopha.2024.116517

s52. Kweon M, Lee H, Park C, Choi YH, Ryu JH. A Chalcone from Ashitaba (Angelica keiskei) Stimulates Myoblast Differentiation and Inhibits Dexamethasone-Induced Muscle Atrophy. Nutrients. 2019;11:2419. doi:10.3390/nu11102419

s53. Geng H, Song Q, Cheng Y, Li H, Yang R, Liu S, et al. MicroRNA 322 Aggravates Dexamethasone-Induced Muscle Atrophy by Targeting IGF1R and INSR. Int J Mol Sci. 2020;21:111. doi:10.3390/ijms21031111

s54. Cai R, Zhang Q, Wang Y, Yong W, Zhao R, Pang W. Lnc-ORA interacts with microRNA-532-3p and IGF2BP2 to inhibit skeletal muscle myogenesis. J Biol Chem. 2021;296:100376. doi:10.1016/j.jbc.2021.100376

s55. Ma K, Mallidis C, Artaza J, Taylor W, Gonzalez-Cadavid N, Bhasin S. Characterization of 5'-regulatory region of human myostatin gene: regulation by dexamethasone in vitro. Am J Physiol Endocrinol Metab. 2001;281:E1128–36. doi:10.1152/ajpendo.2001.281.6.E1128

s56. Salehian B, Mahabadi V, Bilas J, Taylor WE, Ma K. The effect of glutamine on prevention of glucocorticoid-induced skeletal muscle atrophy is associated with myostatin suppression. Metabolism. 2006;55:1239–47. doi:10.1016/j.metabol.2006.05.009

s57. Chang YB, Ahn Y, Suh HJ, Jo K. Yeast hydrolysate ameliorates dexamethasone-induced muscle atrophy by suppressing MuRF-1 expression in C2C12 cells and C57BL/6 mice. Journal of Functional Foods. 2022;90:104985.

s58. Furukawa K, Kousaka M, Jia H, Kato H. Suppressive Effects of Turmeric Extract on Muscle Atrophy in Dexamethasone-Treated Mice and Myotubes. Nutrients. 2022;14:3979. doi:10.3390/nu14193979

s59. Xie Y, Perry BD, Espinoza D, Zhang P, Price SR. Glucocorticoid-induced CREB activation and myostatin expression in C2C12 myotubes involves phosphodiesterase-3/4 signaling. Biochem Biophys Res Commun. 2018;503:1409–14. doi:10.1016/j.bbrc.2018.07.056

s60. Zhao W, Pan J, Zhao Z, Wu Y, Bauman WA, Cardozo CP. Testosterone protects against dexamethasone-induced muscle atrophy, protein degradation and MAFbx upregulation. J Steroid Biochem Mol Biol. 2008;110:125–9. doi:10.1016/j.jsbmb.2008.03.024

s61. Hinds TD, Peck B, Shek E, Stroup S, Hinson J, Arthur S, et al. Overexpression of Glucocorticoid Receptor β Enhances Myogenesis and Reduces Catabolic Gene Expression. Int J Mol Sci. 2016;17:232. doi:10.3390/ijms17020232

s62. Otsuka Y, Egawa K, Kanzaki N, Izumo T, Rogi T, Shibata H. Quercetin glycosides prevent dexamethasone-induced muscle atrophy in mice. Biochem Biophys Rep. 2019;18:100618. doi:10.1016/j.bbrep.2019.100618

s63. Hudson MB, Rahnert JA, Zheng B, Woodworth-Hobbs ME, Franch HA, Price SR. miR-182 attenuates atrophy-related gene expression by targeting FoxO3 in skeletal muscle. Am J Physiol Cell Physiol. 2014;307:C314–9. doi:10.1152/ajpcell.00395.2013

s64. Polge C, Heng AE, Jarzaguet M, Ventadour S, Claustre A, Combaret L, et al. Muscle actin is polyubiquitinylated in vitro and in vivo and targeted for breakdown by the E3 ligase MuRF1. FASEB J. 2011;25:3790–802. doi:10.1096/fj.11-180968

s65. Yu R, Chen JA, Xu J, Cao J, Wang Y, Thomas SS, et al. Suppression of muscle wasting by the plant-derived compound ursolic acid in a model of chronic kidney disease. J Cachexia Sarcopenia Muscle. 2017;8:327–41. doi:10.1002/jcsm.12162

s66. Norikura T, Sasaki Y, Kojima-Yuasa A, Kon A. Glyoxylic Acid, an α-Keto Acid Metabolite Derived from Glycine, Promotes Myogenesis in C2C12 Cells. Nutrients. 2023;15:1763. doi:10.3390/nu15071763

s67. Waddell DS, Baehr LM, van den Brandt J, Johnsen SA, Reichardt HM, Furlow JD, et al. The glucocorticoid receptor and FOXO1 synergistically activate the skeletal muscle atrophy-associated MuRF1 gene. Am J Physiol Endocrinol Metab. 2008;295:E785–97. doi:10.1152/ajpendo.00646.2007

s68. Zeng R, Xu H, Wu M, Zhou X, Lei P, Yu J, et al. Entacapone alleviates muscle atrophy by modulating oxidative stress, proteolysis, and lipid aggregation in multiple mice models. Front Physiol. 2024;15:1483594. doi:10.3389/fphys.2024.1483594

s69. Sugiyama M, Yamaki A, Furuya M, Inomata N, Minamitake Y, Ohsuye K, et al. Ghrelin improves body weight loss and skeletal muscle catabolism associated with angiotensin II-induced cachexia in mice. Regul Pept. 2012;178:21–8. doi:10.1016/j.regpep.2012.06.003

s70. Park E, Choi H, Truong CS, Jun HS. The Inhibition of Autophagy and Pyroptosis by an Ethanol Extract of Nelumbo nucifera Leaf Contributes to the Amelioration of Dexamethasone-Induced Muscle Atrophy. Nutrients. 2023;15:804. doi:10.3390/nu15040804

s71. Hemdan DI, Hirasaka K, Nakao R, Kohno S, Kagawa S, Abe T, et al. Polyphenols prevent clinorotation-induced expression of atrogenes in mouse C2C12 skeletal myotubes. J Med Invest. 2009;56:26–32. doi:10.2152/jmi.56.26

s72. Tobimatsu K, Noguchi T, Hosooka T, Sakai M, Inagaki K, Matsuki Y, et al. Overexpression of the transcriptional coregulator Cited2 protects against glucocorticoid-induced atrophy of C2C12 myotubes. Biochem Biophys Res Commun. 2009;378:399–403. doi:10.1016/j.bbrc.2008.11.062

s73. Pierucci F, Frati A, Battistini C, Penna F, Costelli P, Meacci E. Control of Skeletal Muscle Atrophy Associated to Cancer or Corticosteroids by Ceramide Kinase. Cancers (Basel). 2021;13:3285. doi:10.3390/cancers13133285

s74. Pierucci F, Frati A, Battistini C, Matteini F, Iachini MC, Vestri A, et al. Involvement of released sphingosine 1-phosphate/sphingosine 1-phosphate receptor axis in skeletal muscle atrophy. Biochim Biophys Acta Mol Basis Dis. 2018;1864:3598–614. doi:10.1016/j.bbadis.2018.08.040

s75. Qin W, Pan J, Qin Y, Lee DN, Bauman WA, Cardozo C. Identification of functional glucocorticoid response elements in the mouse FoxO1 promoter. Biochem Biophys Res Commun. 2014;450:979–83. doi:10.1016/j.bbrc.2014.06.080
